# Gut Microbiota as Well as Metabolomes of Wistar Rats Recover within Two Weeks after Doripenem Antibiotic Treatment

**DOI:** 10.3390/microorganisms11020533

**Published:** 2023-02-20

**Authors:** Aishwarya Murali, Franziska Maria Zickgraf, Philipp Ternes, Varun Giri, Hunter James Cameron, Saskia Sperber, Volker Haake, Peter Driemert, Hennicke Kamp, Dorothee Funk Weyer, Shana J. Sturla, Ivonne M. G. M. Rietjens, Bennard van Ravenzwaay

**Affiliations:** 1 BASF SE, 67056 Ludwigshafen, Germany; 2 BASF Metabolome Solutions GmbH, 10589 Berlin, Germany; 3 BASF Plant Science LP, Research Triangle Park, Raleigh, NC 27709, USA; 4 ETH Zürich, Department of Health Sciences and Technology, Schmelzbergstrasse 9, 8092 Zurich, Switzerland; 5 Department of Toxicology, Wageningen University & Research, 6703 HE Wageningen, The Netherlands

**Keywords:** metabolomics, gut microbiome, carbapenems, gut microbiota and metabolome recovery, repeated dose oral study

## Abstract

An understanding of the changes in gut microbiome composition and its associated metabolic functions is important to assess the potential implications thereof on host health. Thus, to elucidate the connection between the gut microbiome and the fecal and plasma metabolomes, two poorly bioavailable carbapenem antibiotics (doripenem and meropenem), were administered in a 28-day oral study to male and female Wistar rats. Additionally, the recovery of the gut microbiome and metabolomes in doripenem-exposed rats were studied one and two weeks after antibiotic treatment (i.e., doripenem-recovery groups). The 16S bacterial community analysis revealed an altered microbial population in all antibiotic treatments and a recovery of bacterial diversity in the doripenem-recovery groups. A similar pattern was observed in the fecal metabolomes of treated animals. In the recovery group, particularly after one week, an over-compensation was observed in fecal metabolites, as they were significantly changed in the opposite direction compared to previously changed metabolites upon 28 days of antibiotic exposure. Key plasma metabolites known to be diagnostic of antibiotic-induced microbial shifts, including indole derivatives, hippuric acid, and bile acids were also affected by the two carbapenems. Moreover, a unique increase in the levels of indole-3-acetic acid in plasma following meropenem treatment was observed. As was observed for the fecal metabolome, an overcompensation of plasma metabolites was observed in the recovery group. The data from this study provides insights into the connectivity of the microbiome and fecal and plasma metabolomes and demonstrates restoration post-antibiotic treatment not only for the microbiome but also for the metabolomes. The importance of overcompensation reactions for health needs further studies.

## 1. Introduction

The human gut harbors thousands of different species of bacteria, fungi, archaea, and viruses, where bacteria exceed eukaryotes and archaea by 2–3 orders of magnitude, forming a complex community known as the gut microbiota [1,2]. While intestinal microbiota performs several vital metabolic functions crucial to the host [3,4], its composition is highly variable and susceptible to dysbiosis, which results from pressures such as diet, immune function, environmental chemical exposures, and the use of drugs. Furthermore, microbial dysbiosis is associated with human diseases such as Crohn’s disease, colon cancer, diabetes, allergies, Alzheimer’s and Parkinson’s disease, depression, and stroke [5,6,7]. Moreover, the ability of the gut microbiota to (bio)transform molecules is relevant for pharmacology and toxicology [8].

Antibiotics, in particular, are known to compositionally as well as functionally (metabolism-wise) affect the gut microbiota [9,10]. This makes antibiotics ideal compounds to induce a temporary shift in the gut composition and to determine the changes in metabolic output. In previous studies, we have shown how different classes of antibiotics, including aminoglycosides, fluoroquinolones, lincosamides, and polymyxin E, induce selective changes in the gut microbiome of experimental animals and how these are reflected by antibiotic class-specific changes in the fecal and plasma metabolomes [1,11,12,13]. Several antibiotics belonging to classes including aminoglycosides, fluoroquinolones, lincosamides, and polymyxin E have been studied to understand their effects on the gut microbiota composition as well as the related metabolic outputs [1,14,15].

Two antibiotics belonging to the carbapenem class have been selected for the present study as this class has not been investigated before. Both meropenem and doripenem are broad-spectrum carbapenem antibiotics that are active against Gram-positive and Gram-negative bacteria, and these antibiotics have poor oral bioavailability from the gastrointestinal tract [16,17,18]. To selectively identify metabolome changes in the plasma because of changes in the microbiome, antibiotics with low bioavailability are preferred to avoid plasma metabolome changes related to the potential systemic toxicity of the antibiotics. Several clinical trials have previously demonstrated that carbapenems have low oral bioavailability and hence must be administered intravenously because they cannot cross gastrointestinal membranes readily [17,19]. The shift in the gut microbiota composition induced by antibiotics can be determined using 16S marker gene sequencing of fecal samples and the corresponding changes in metabolites can be assessed using metabolomics. Highly sensitive mass spectrometry-based (MS) techniques allow for the detection of a broad range of metabolites, such as amino acids, bile acids, carbohydrates, lipids, vitamins, energy metabolites, hormones, and their derivatives. In the current study, we have used an optimized procedure to determine the fecal and plasma metabolome upon antibiotic-induced microbiome changes.

Currently, the existing knowledge on the ability of the gut bacterial communities to spontaneously recover after antibiotic treatment is stopped is not very well defined [20,21]. In both humans and mice, Suez et al. 2018 observed faster spontaneous recovery of the indigenous stool/mucosal microbiome when administering a probiotic. Legrand et al. 2020, also observed strain-specific recovery of the human gastrointestinal tract after antibiotic treatment is stopped [21,22]. It is important to not only understand this recovery pattern of gut bacterial strains following antibiotic treatment but also to characterize the associated metabolite recoveries as they may be an important factor for long-term host health. Understanding the restoration abilities of intestinal and metabolic homeostasis are necessary to be understood in the context of dysbiosis resulting from broad-spectrum antibiotics, as it lays the groundwork for potential strategies that could reduce the long-term adverse effects of antibiotic treatment on the gut health [23,24]. Furthermore, there is a lack of knowledge in the direction of metabolite recovery, and the consequences of microbiome recovery on fecal and plasma metabolomes are also not known, hence they are essential to be understood.

In previously published works, the antibiotic classes aminoglycosides, fluoroquinolones, lincosamides, and macrolides and their effects on the gut microbiome were studied [1,11,12,15]. Here, we expand the previous work by investigating the effects of the carbapenem class. The main objective of this study is to understand the contribution of gut bacterial communities to metabolite production by addressing large-scale quantification of dynamic changes in microbial metabolites as a functional component to community profiling (or taxonomics) analysis. Antibiotics were selected to induce a shift in the gut composition and functions, and the criteria for selection included their ability to induce perturbation in the gut microbiota, low or no systemic bioavailability, and a low/no systemic toxicity. The latter two points were important to avoid systemic toxicity influencing the plasma metabolome profiles.

We assessed perturbations in the gut composition by orally administering meropenem trihydrate and doripenem hydrate to young adult male and female rats in a 28-day study and its consequences on fecal and plasma metabolomes. Young adult Wistar rats have been selected for this study because rodent models are widely used in the assessment of chemical safety assessments for humans and have significantly contributed to delineating correlations between the intestinal microbiome and associated diseases [25]. Finally, we characterized the capacity of the microbiome and its associated plasma and fecal metabolomes to spontaneously recovery after one or two weeks following the cessation of doripenem exposure. These findings show that not only the gut microbiota but also the metabolomes can be restored post-antibiotic treatment, evidencing the interconnection between the gut microbiome and metabolome.

## 2. Materials and Methods

A 28-day oral study using Wistar rats with two further weeks of recovery for the doripenem-exposed animals was carried out based on the OECD 407 guidelines under the principles of Good Laboratory Practice of the German Chemicals Act.

### 2.1. Ethics Statement

This animal study was performed in an AAALAC-approved (Association for Assessment and Accreditation of Laboratory Animal Care International) laboratory in compliance with the German Animal Welfare Act and the effective European Council Directive. This study was approved by the BASF Animal Welfare Body, with the permission of the local authority, the Landesuntersuchungsamt Koblenz, Germany (approval number 23 177-07/G 18-3-098) [13].

### 2.2. Animals and Maintenance Conditions

Wistar rats of both sexes (CrI:WI(Han)) of the age group 70 ± 1 days were purchased from Charles River, Germany, prior to the start of the planned study. The handling of the animals, acclimatization, conditions of the animal test facility, and other related information regarding the study have been presented and published by Murali et al. 2022 [13].

### 2.3. Treatment of Animals with Drugs

**Figure 1:**
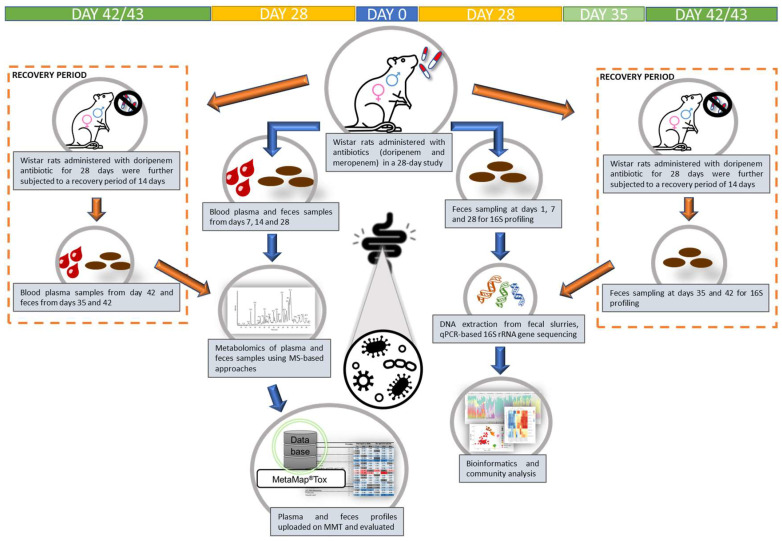
Schematic representation of the study design for plasma and fecal metabolome analyses and the corresponding gut (fecal) 16S bacterial profiling in a 28-day oral gavage study using meropenem- and doripenem- (administered at high and low doses) treated male and female young adult Wistar rats. Note: For doripenem, an additional one- and two-week recovery period, as indicated by the orange dashed boxes, was included to analyze the reversibility. The top row indicates sampling timeline.

In this study, five rats per treatment group per sex were exposed (orally, with gavage) to the two antibiotics (meropenem trihydrate and doripenem hydrate) on a daily basis (see Table 1 and Figure 1). Meropenem was prepared for oral administration by resuspending it in deionized water at doses of 100 mg/kg body weight/day and 300 mg/kg body weight/day. Doripenem was also prepared in deionized water for oral administration at doses of 100 mg/kg body weight/day and 1000 mg/kg body/day weight. Antibiotic dose levels were selected, based on the available literature, to alter the targeted microbiome population without causing systemic toxicity. An antibiotic suspension of 10 mL/kg bw/day was administered orally to five rats per treatment group per dose group per sex each with a corresponding control group of ten rats per sex, to allow direct comparisons.

### 2.4. Clinical Examination

All the animals were checked daily for any signs of clinical toxicity, abnormal macroscopic findings, and mortalities. Standard operating procedures following the Organization for Economic Co-operation and Development (OECD) 407 guidelines were used for all clinical and pathological examinations. Details related to regular examinations have been followed based on our previous publication, Murali et al. 2022 [13].

### 2.5. Blood and Fecal Sampling for Metabolite Profiling

On days 7, 14, and 28, and additionally on days 42/43 for the doripenem groups only, after a fasting period of 16–20 h, collection of blood samples was performed between 7:30 and 10:30 h from the retro-orbital sinus of all the rats (including controls and all treatment groups) under isoflurane anesthesia (1.0 mL K-EDTA blood). After centrifugation (10 °C, 20,000× *g*, 2 min), EDTA plasma was separated from the collected blood samples. After snap-freezing the EDTA plasma samples with N_2_, they were stored at 80 °C for metabolite profiling. Following gentle massaging of the rectum, feces were collected from rats exposed to different antibiotic exposures on days 7, 14, and 28; from the doripenem-recovery group, feces were collected from females on days 35 and 42 and from males on day 43. Following 16–20 h of fasting, if no feces samples could be obtained, then the samples were simply taken by scraping from the rat colons on the day of necropsy, i.e., on days 28 and 42/43. Pre-cooled (dry ice) vials were filled with these samples, which were then snap-frozen in liquid N_2_ and stored at 80 °C until processing for fecal dry weight measurements, and further to perform DNA isolation and 16S gene PCR amplification and sequencing.

### 2.6. DNA Isolation and 16S Marker Gene Sequencing

Dry weights of stored fecal samples collected on days 1, 7, 28, 35, and 42/43 from all controls and antibiotic-treated rats were measured and then transferred to labeled pre-cooled (dry-ice) DNA/RNA Shield-Lysis collection tubes (BIOZOL Diagnostica Vertrieb GmbH, Eching, Germany) avoiding excess thawing of the frozen fecal pellets. The processing of fecal slurries for further DNA isolation, quantification, sequencing, and the final report produced was based on the process previously published by Murali et al. 2022 [13].

### 2.7. Metabolomics of Plasma and Feces Matrices and Statistical Analysis

Mass spectrometry-based metabolite profiling of blood plasma and feces samples collected from the controls, antibiotic-treated, and recovery groups was performed according to the protocol published previously by Murali et al. 2022 [13,26,27].

In plasma, 272 semiquantitative metabolites were analyzed using the single peak signal of the respective metabolite and a normalization strategy according to the patent [28] resulting in fold change ratios representing the metabolite changes in treated versus control animals [1]. Of these 306 metabolites, the chemical structures of 280 were identified and 26 remained unknown. In feces, 632 semiquantitative metabolites could be analyzed, out of which the chemical structures of 348 were identified and 284 were unknown.

Furthermore, statistical analysis of the metabolome data of plasma and feces matrices, as well as the uploading of these data into our in-house database MetaMapTox^®^ (or MMT), followed by a PCA analysis were performed following the protocol published by Murali et al. 2022 [1,12,13,29].

### 2.8. The 16S Data Processing and Statistical Analysis of the Data

The data processing pipeline used was an in-house standardized algorithm using DADA2 for 16S marker gene data as published in Murali et al. 2022 [12]. The customized workflow and further steps including a quality check (QC) followed by taxonomic assignments were conducted based on the previous publication by Murali et al. 2022 [13]. The overall methods used followed the publication by Murali et al. 2022.

Furthermore, post-processing of the 16S data was performed using different packages, as presented in Murali et al. [12]. The entire raw data used for the study consisted of 5,027,882 reads belonging to 5533 taxa from a total of 251 samples. Reads with non-zero counts on at least two samples were retained, and the rest were removed as a part of the cleaning up of the raw data, which resulted in 4,429,863 reads belonging to 2053 taxa from a total of 251 samples. Furthermore, reads that were not assigned to a minimum of the family level of taxonomy were removed, resulting in 4,429,863 reads belonging to 2053 taxa from 251 samples. Further analysis including diversity, relative, and differential abundance analyses following the protocol published by Murali et al. 2022 [13,30].

## 3. Results

### 3.1. No Visible Signs of Toxicity or Change in the Body Weight or Food Consumption after Administering Carbapenems

**Figure 2:**
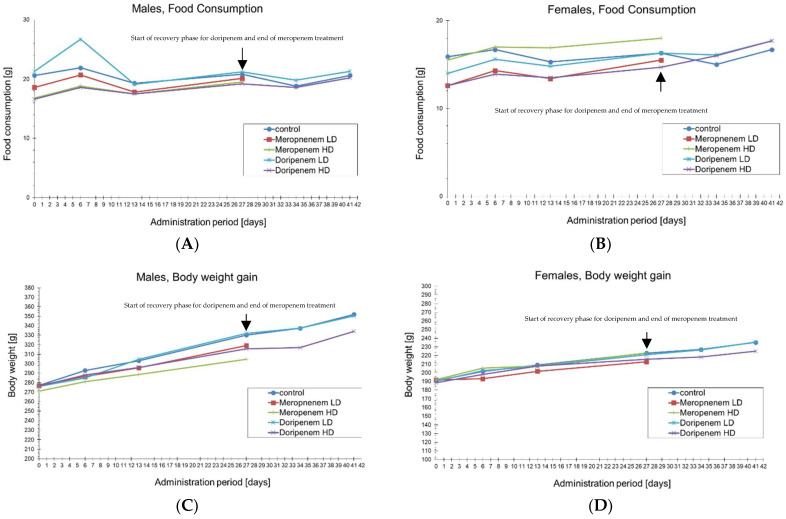
Line plots representing food consumption (in grams) and body weight (in grams), in male (**A**,**C**) and female (**B**,**D**) animals, respectively, compared to the controls and different antibiotics treatment groups on days 0, 6, 13, and 27 and additionally on days 35 and 42/43 for the doripenem-recovery groups. The end of meropenem treatment and the start of doripenem-recovery groups have been highlighted with black arrows. Changes in body weight and food consumption were compared between control and treated groups and showed no significant differences (*p*-value < 0.05).

After administering meropenem and doripenem to male and female rats for 28 days, there were no mortalities nor signs of clinical toxicity, except for one male rat (no. 55) receiving doripenem LD. This animal developed a lower jaw lesion and lost body weight; therefore, it was excluded from the study on day 7. All sampled feces had a normal consistency. Food consumption and body weight values of male (A,C) and female (B,D) animals from the two antibiotic treatment groups relative to the control animals, respectively, have been presented (Figure 2). During the 28-day study, both control and antibiotic-treated animals maintained roughly the same body weights and food consumption rates. Animals belonging to the group that was administered doripenem and then allowed a recovery period without daily antibiotic administration for a period of two weeks, i.e., the doripenem-recovery group, also consumed a similar amount of food and gained a similar amount of weight as the control animals.

### 3.2. Bacterial Diversity Was Reduced by Carbapenem Treatment and Returned to Control Levels in the Doripenem-Recovery Group

**Figure 3:**
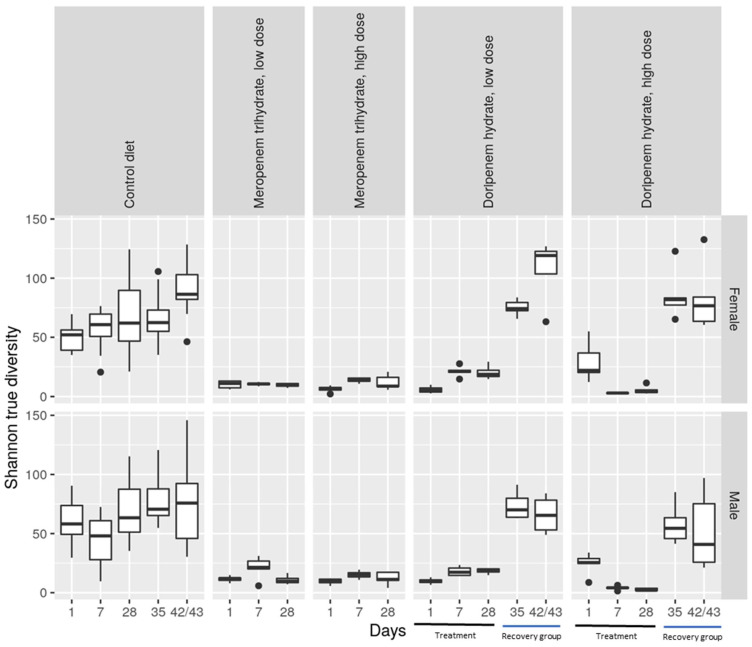
Shannon true diversity analysis of the antibiotic treatments, meropenem, doripenem, and controls for both male and female Wistar rats. The top row shows data for females (f) and the bottom row shows data for males (m). Doripenem treatment time points (days 1, 7, and 28) and recovery time points (days 35 and 42/43) are indicated with black and blue lines, respectively, on the x-axis. Whiskers denote standard deviations, and solid lines within the boxes indicate the group medians. The dots falling outside the boxes demonstrate the most extreme values.

Alpha diversity, representing the diversity of bacterial taxa present in a specific condition or treatment group, was evaluated using the Shannon true diversity algorithm (Figure 3). The size of the boxes in the plot represents the variability between the samples from individual animals of a specific group. The higher the diversity, the greater the presence of different bacterial taxa. The increase in alpha diversity of microbiota from fecal samples collected from day 1 of the study to day 42/43 in both sexes demonstrated a greater presence of differential taxa over the course of the study. As anticipated, compared to the microbiota from the controls, the alpha diversity of the microbiota from animals belonging to all the antibiotic groups from days 1 to 28 shows a clear reduction. There was no clear dose-dependent effect concerning the two dose levels of the antibiotics. Furthermore, after the animals administered with doripenem were allowed a drug-free recovery period of one or two weeks, there was a corresponding increase in alpha diversity to a level similar to that in the control animals for both sexes (Figure 3).

**Figure 4:**
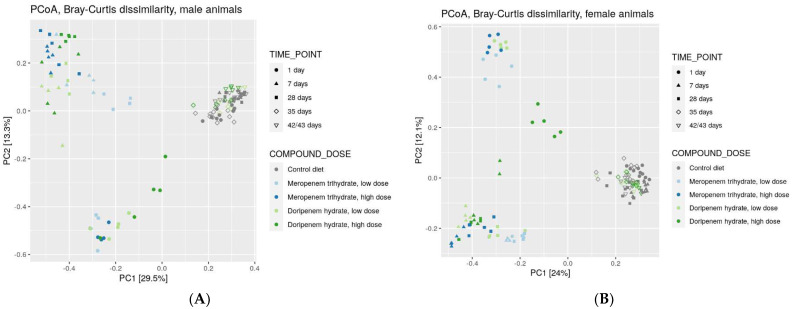
Principal Coordinate Analysis (PCoA) of bacterial families from controls, meropenem, and doripenem antibiotic-treated rats at different time points. Rank-based clustering with Bray-Curtis distance matrix of samples from (**A**) males and (**B**) females are presented.

To compare the diversity between different ecosystems, as might be present in the different treatment groups, a rank-based Principal Coordinate Analysis (PCoA) was carried out using a non-phylogenetic Bray-Curtis distance matrix. The results of this analysis were consistent with observations for alpha diversity, including the emergence of treatment-specific clusters (Figure 4), irrespective of the dose of the two antibiotics. Overall, at least three distinct clusters were apparent in the PCoA plot for males and females. All control microbiota from all time points clustered together, whereas day 1 microbiota from animals treated with antibiotics formed a separate cluster. The days 7 and 28 microbiota from animals treated with antibiotics clustered together and separated both from controls and day 1 treated animals for both sexes. These observations have several implications: (1) Doripenem and meropenem have similar effects on bacterial taxa, confirming their common mode of action. (2) Beta diversity composition on day 1 is different from days 7 and 28, suggesting that the treatment effects had not yet emerged on day 1. (3) A fourth cluster was observed, which was more distinct in females, that consisted of day 1 microbiota from the doripenem HD group and indicated a possible dose-response one day after the administration of doripenem HD. Upon prolonged exposure, this dose-dependency could no longer be observed. (4) Finally, microbiota from control animals on days 35 and 42/43 clustered together with the doripenem-recovery groups one and two weeks after the cessation of doripenem treatment.

### 3.3. Reduced Relative Bacterial Abundances Following Carbapenem Treatment Is Reversible

**Figure 5:**
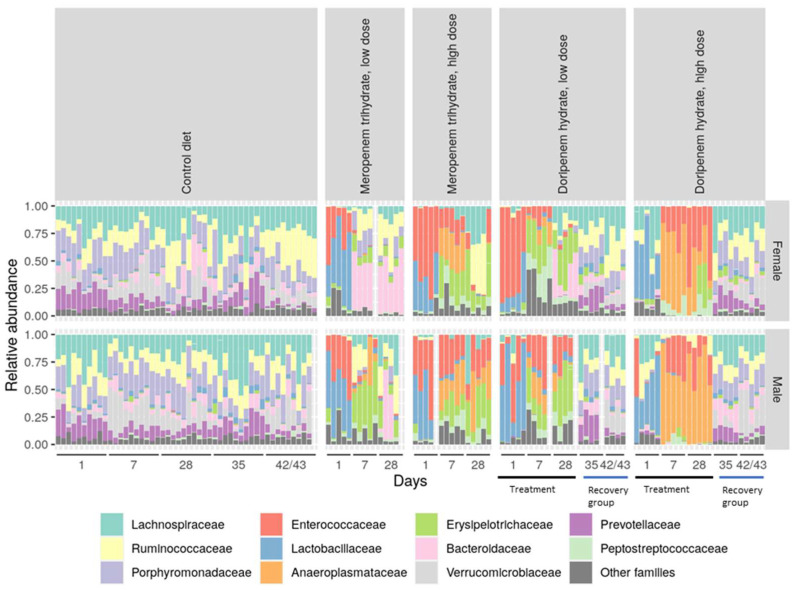
Stacked bar diagram showing relative abundances of the detected bacterial families in the different experimental groups. Inter-individual variability in the composition can be observed. Dose groups and sexes showed significant differences specifically in samples from days 1, 7, and 28 relative to the controls. Antibiotic-specific effects were observed on the intestinal community composition. For the doripenem group, treatment time points, i.e., days 1, 7, and 28, and recovery time points, i.e., 35 and 42/43, are highlighted with black and blue lines on the x-axis, respectively.

Having established a general comparison of bacterial diversity between the control and different treatment groups, the relative abundances of different dominant bacterial families were evaluated (Figure 5). This revealed a profound inter-individual variability in relative levels of different bacterial families in all treatment and control groups. Bacterial abundance was reduced after day 1 treatment with both meropenem and doripenem and increased on days 7 and 28. Bacterial families including *Lactobacillaceae*, *Porphyromonadaceae*, and *Prevotellaceae* showed a strong depletion in microbiota from days 7 and 28 for all carbapenem treatment groups. For day 1 microbiota from the meropenem LD group, the start of a dysbiosis in the bacterial communities was observed in both sexes, whereas on days 7 and 28, the microbiota of the females reached a new and stable gut composition, whereas this took longer for males. Furthermore, the day 1 doripenem LD microbiota also exhibited an intermediate dysbiosis, whereas an altered but stable bacterial composition was observed on days 7 and 28 for both sexes. Such a transition or intermediate composition of the gut microbiome was not observed for the animals administered high doses of carbapenems.

**Figure 6:**
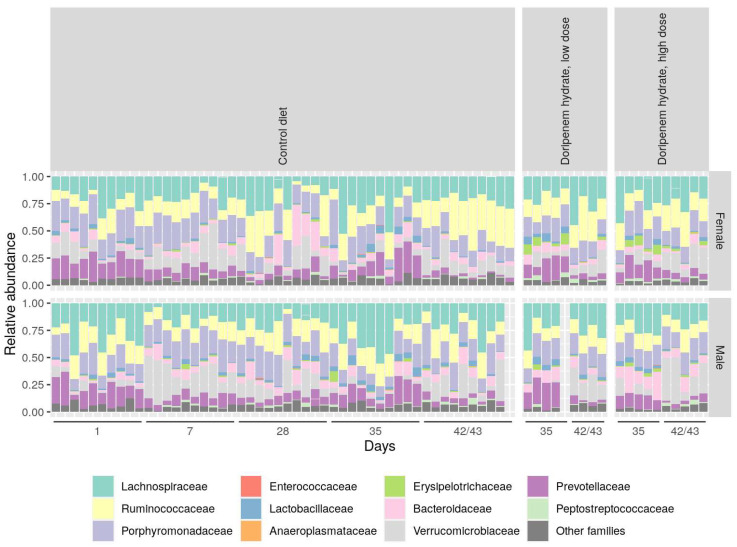
Stacked bar diagram showing relative abundances of bacterial families that were detected in the controls and doripenem-recovery groups. An indication of the recovery of abundant bacterial families one and two weeks post-doripenem treatment was observed and resulted in abundances comparable to the controls.

In the doripenem-recovery groups, bacterial abundances were comparable to the microbiota from the control group, whereas the microbiota on day 35 (one week after the stop of doripenem treatment) still showed minor differences. This observation was more pronounced in female animals (Figure 6). Overall, the analysis suggested that the bacterial abundances in microbiota after one and two weeks of the cessation of doripenem treatment in male rats may completely recover to have a 16S composition comparable to the control group. The doripenem-recovery groups also indicated a recovery two weeks after doripenem treatment was stopped in female animals.

### 3.4. Differential Abundance Analyses Confirmed Spontaneous Recovery Post-Doripenem Cessation

**Figure 7:**
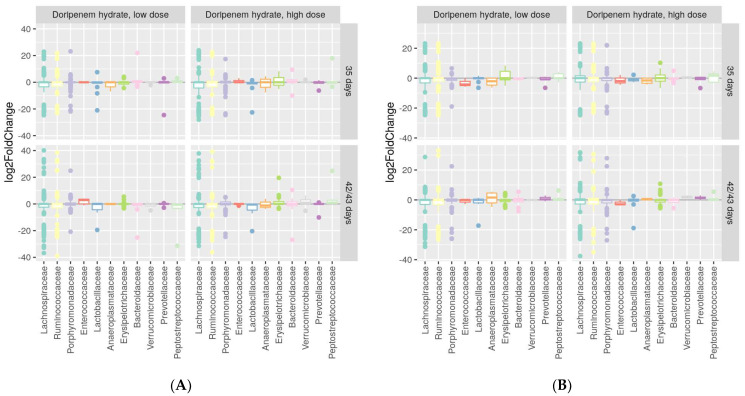
Differential abundances in dominant bacterial families one and two weeks post-doripenem-treated samples from the low- (LD) and high- (HD) dose groups relative to the controls for males (**A**) and females (**B**). Differential abundance analysis was performed using log2FC (log2 fold change) values.

Differential abundance analysis using the DESeq2 package was performed to identify whether increases or decreases in specific bacterial families were associated with specific treatment groups compared to the controls. The differential abundance analyses of both meropenem- and doripenem-treated microbiota on days 1, 7, and 28 of the study from both male and female animals can be found in the Supplementary Materials (Figure S2). The differential analyses confirmed an absence of differences compared to controls for the male rats given a recovery period, i.e., days 35 and 43 of the study (Figure 7A,B, respectively). These results indicated recovery of the previously altered bacterial families including *Lactobacillaceae*, *Porphyromonadaceae*, and *Prevotellaceae*, to their original or baseline levels after one week for males and persisting two weeks post-doripenem cessation. On the other hand, for females, after one-week post-doripenem cessation, there were still a few changes in bacterial families, including *Enterococcaceae* and *Aneroplasmataceae*, which both recovered fully two weeks post-doripenem cessation.

### 3.5. Fecal Metabolomes Are Altered by Carbapenems and Spontaneously Recover after Doripenem Treatment Is Stopped

**Figure 8:**
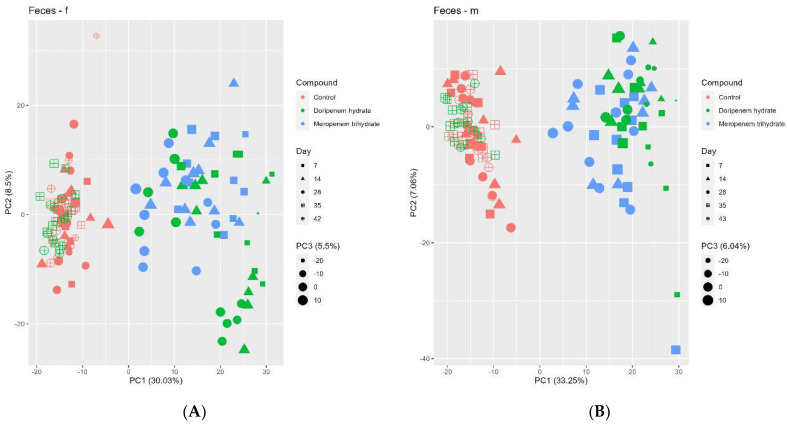
Principal component analysis (PCA) of the fecal metabolome profiles of controls (red), meropenem- (blue), and doripenem- (green) treated rats. PCA for fecal metabolome profiles of (**A**) female and (**B**) male Wistar rats on days 7, 14, and 28 of the study and days 35 and 42/43 for the doripenem-recovery group. The data points are sized by the PC3 value. Different shapes of the data points represent respective sampling time points. Dose group-specific effects were marginal, so the groups were not separated based on the dose levels.

Principal Component Analysis or PCA plots of fecal metabolome data from days 7, 14, and 28 (Figure 8) indicated a clear separation between the controls and the antibiotic-treated animals in PC1. In the doripenem HD females, a further separation (separating in PC2) could be observed particularly for fecal metabolomes on days 14 and 28. The metabolome profiles of feces from the doripenem-recovery groups clustered together with the controls indicating full recovery of the fecal metabolome within one week after the cessation of treatment. This observation is in line with the conclusions from the 16S community analyses, specifically for the male rats where a full recovery was observed one week after doripenem cessation.

#### 3.5.1. Meropenem Trihydrate Treatment Alters Fecal Bile Acids

The analysis of 620 different metabolites in feces revealed significant alterations upon antibiotic treatment. Of these, approx. 300 were significantly altered by the meropenem HD treatment in both sexes. The complete list of significantly changed fecal metabolites in meropenem-treated male and female animals is presented in the Supplementary Materials (Tables S1 and S2), where, for females in particular, the majority of the significantly changed fecal metabolites were amino acids, bile acids (BAs), lipids, energy metabolites, vitamins, and related metabolite classes. The list of significantly changed BAs in meropenem-treated female rats is presented in Table 2 using the HD group as reference for change against the controls. Of the 22 measured BAs, 11 were significantly changed compared to the control group, including the secondary (2°) BAs, deoxycholic acid (DCA), lithocholic acid (LCA), ω-muricholic acid (ωMCA), hyodeoxycholic acid (HDCA), and isoLCA. These 2° BAs were significantly reduced in the fecal metabolomes from almost all the sampling time points from both the meropenem LD and HD treatment groups. Several taurine-conjugated primary (1°) and 2° BAs were significantly increased; particularly, TCA (taurocholic acid sodium salt and taurocholic acid-3-sulfate) (TCA) was strongly and significantly increased in fecal metabolomes from all the time points from both the meropenem LD and HD treatment groups.

In males, almost 300 fecal metabolites were significantly changed, the majority of which belonged to amino acids, BAs, lipids, carbohydrates, energy metabolites, nucleobases, vitamins, and their derivatives. Similar to females, 11 out of the 22 measured fecal bile acids were observed to be significantly altered at a *p*-value < 0.05 (Table 3). In males, a significant reduction was observed in the 2° BAs, including DCA, LCA, ωMCA, HDCA, and isoLCA in both the meropenem LD and HD groups compared to the controls. A significant increase was observed in a few 1° BAs including CA, α-muricholic acid (αTMCA), and β-muricholic (βMCA) and also in some of the taurine-conjugated 1° BAs such as TCA and tauro-β-muricholic acid (TβMCA) on almost all sampling time points in both the meropenem LD and HD treatment groups.

Overall, the fecal metabolome of the meropenem-treated rats appeared to have strongly and significantly changed compared to the control group, which included significant changes in the BAs.

#### 3.5.2. Doripenem Hydrate Treatment Significantly Alters Fecal BA Metabolites, Which Recover after the Cessation of Treatment

About 620 metabolites were measured in the fecal samples from the doripenem-treated Wistar rats, out of which about 350 fecal metabolites were significantly changed at a *p*-value < 0.05 in all groups. The majority of these fecal metabolites belong to amino acids, BAs, lipids, carbohydrates, nucleobases, vitamins, and their related classes. The complete list of fold changes in fecal metabolites is presented in the Supplementary Materials (Tables S3 and S4). Out of the 22 measured fecal BAs, 14 were significantly altered at a *p*-value < 0.05 in the HD-treated female rats (Table 4). This consisted of significantly reduced 2° BAs such as DCA, LCA, HDCA, ωMCA, and isoLCA. Consistently, taurine-conjugated 1° BAs including TCA, TβMCA, and taurochenodeoxycholate (TCDCA) were all significantly increased in fecal metabolomes from almost all time points, which was more prominent in the HD-treated females. Some taurine and glycine-conjugated 2° BAs such as taurolithocholic acid (TLCA), taurodeoxycholate (TDCA), glycolithocholic acid (GLCA), and glycodeoxycholate (GDCA) were observed to be significantly reduced in both the LD and HD treatment groups.

In the female doripenem-recovery group, recovery of the formerly significantly changed fecal BAs could be observed, specifically in the LD recovery group (Table 4). However, a few of the BAs that were significantly decreased during the doripenem treatment period, i.e., LCA, isoLCA TCA, GLCA, and TCA, were still significantly altered after the recovery period and were actually increased. Likewise, there were examples in the opposite direction, i.e., some BAs that were significantly increase during the treatment period were significantly reduced compared to the controls after the recovery period. This overcompensation effect observed for the fecal metabolomes may relate to a possible metabolic feedback mechanism.

The doripenem-treated male rats showed significant changes in about 320 fecal metabolites at a *p*-value < 0.05. The majority of these metabolites belonged to amino acids, BAs, carbohydrates, lipids, energy metabolites, nucleobases, vitamins, and their derivatives. The list of significantly changed fecal BAs in samples from the doripenem LD- and HD-treated males and from the doripenem-recovery group relative to the controls at a *p*-value < 0.05 is presented in Table 5. A total of nine significantly changed BAs were observed in males which includes 2° BAs such as DCA, LCA, HDCA, ωMCA, and isoLCA, consistent with females treated with the doripenem antibiotic. Compared to females, a lower number of changes in the conjugated 1° and 2° BAs were observed in males. The 1° BA, CA, and a few taurine-conjugated 1° BAs including TCA and TβMCA were significantly increased in the doripenem LD- and HD-treated male rats compared to the controls. Other than in females, the fecal metabolome of the doripenem-recovery males showed a virtually full recovery on day 43. The observed statistically significant decrease in isoLCA is of questionably nature as such an effect was not seen on day 35 (Table 5). In addition, in the doripenem HD day 35 recovery group, two fecal BAs, HDCA and ωMCA, were significantly changed.

### 3.6. Plasma Metabolome Analysis Also Shows Some Effects of Both Carbapenems on Key Plasma Metabolites and Plasma Bile Acid Levels Followed by a Recovery in the Doripenem-Recovery Group

**Figure 9:**
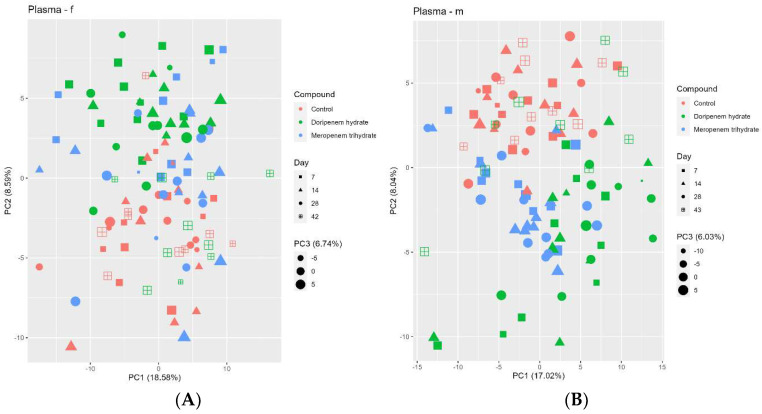
Principal component analysis (PCA) of the blood plasma metabolome profiles of controls (red), meropenem- (blue), and doripenem- (green) treated rats. PCA for plasma metabolome profiles of (**A**) female and (**B**) male Wistar rats on days 7, 14, and 28 of the study and on day 42/43 for the doripenem-recovery group. The data points are sized by the PC3 value. Different shapes of the data points represent respective sampling time points. Dose group-specific effects were marginal, so the groups were not separated based on the dose levels.

The plasma metabolome profiles of the controls and antibiotic treatment groups were analyzed using PCA (Figure 9). Although the separation between the different treatments and controls was not very strong, the plasma metabolome of the control group formed a separate cluster. For males, a good separation between both the treatments and controls (in PC2) can be observed. In addition, there is also some form of separation between the doripenem and meropenem treatment groups (PC1 / PC2). For females, a separation of both treatments from the controls can be seen (in PC2) with, however, a few exceptions (all related to meropenem). For both sexes overall, the doripenem groups are better separated from the controls indicating a possible stronger effect of doripenem on the plasma metabolome profiles of the male and female Wistar rats. There is no clear separation on PC1 for both treatments. The samples from the doripenem-recovery group (i.e., on day 42/43 of the study) clustered together with the controls, which is consistent with the results of the 16S community analysis and fecal metabolome analysis, indicating recovery from the treatment-related changes. 

#### 3.6.1. Meropenem Trihydrate Treatment Alters Key Plasma Biomarkers of Microbiota Alterations

An in-depth analysis of individual plasma metabolites, including BAs that were significantly altered in the meropenem group (*p*-value < 0.05), was performed. Of 294 measured blood plasma metabolites, 27 were significantly altered in the meropenem HD treatment groups in females and 25 in males (Table 6 and Table 7). These were determined by comparing the HD group with the controls and subsequently analyzing the values of these metabolites for the LD. Most of these altered metabolites belonged to amino acids, BAs, carbohydrates, energy metabolites, hormones, and their derivatives.

In the plasma metabolomes of female animals, some of the gut microbiome-associated key plasma biomarkers, as determined in previous studies, were observed to be significantly changed [11,14,15]. These biomarkers include indole-3-acetic acid (IAA), 3-indoxylsulfate (3IS), hippuric acid (HA), and 3-hydroxybuturate. In particular, 3IS and HA were significantly reduced in the meropenem HD- and LD-treated females, especially in plasma metabolomes on days 7 and 14. IAA showed a slight yet significant increase in the meropenem treatment groups at almost all sampling time points, while 3-hydroxybutyrate showed only a significant increase in plasma metabolomes on days 7 and 14 in the meropenem HD treatment group relative to the controls. Other essential gut microbiome-associated metabolites, such as BAs, were significantly changed in the meropenem treatment group relative to the controls. Overall, a stronger effect on plasma metabolites was seen in the HD group. The list of 12 significantly altered plasma BAs out of the 22 measured BAs in the meropenem-treated females is listed in Table 6.

The 2° BAs including DCA, LCA, HDCA, ωMCA, and isoLCA were all significantly reduced specifically in all the samples from the meropenem HD-treated females. Taurine-conjugated 2° BAs including TLCA and TDCA were significantly reduced, whereas tauroursodeoxycholic acid (TUDCA) was significantly increased in the treated females. Unlike feces, plasma did not show any significant changes in TCA. Three of the four glycine-conjugated 1° and 2° plasma BAs were increased and one was decreased, none of which were changed in the feces of the treated females.

As indicated earlier, for males treated with meropenem, 25 out of the 294 measured plasma metabolites were significantly altered. The majority of these plasma metabolites belonged to amino acids, BAs, lipids, energy metabolites, hormones, and relative classes (Table 8 and Table 9). In males, three of the plasma key metabolites were significantly changed, including 3IS, IAA, and HA, where HA and 3IS were reduced and IAA was increased in the meropenem-treated males compared to the controls. However, none of the other key biomarkers appeared on the list. In addition, 14 out of the 22 measured plasma BAs were significantly changed in the meropenem-treated males (Table 9). For males, a dose–response relationship was also noted.

Consistent with the observations for females, the 2° BAs including DCA, LCA, HDCA, ωMCA, and isoLCA were all significantly reduced specifically in the plasma metabolomes on days 7 and 14 for the meropenem HD- and day 28 for the meropenem LD-treated males. Several taurine-conjugated 1° (TCA, TβMCA, and TCDCA) and 2° plasma BAs (TCA, TDCA, and TUDCA), and one glycine-conjugated 2° BA, GDCA, were significantly changed specifically in males treated with meropenem HD relative to the controls. TLCA, TDCA, and GDCA were significantly reduced specifically in the plasma metabolomes on days 7 and 28 of the meropenem HD-treated males compared to the controls, whereas TβMCA and TCDCA were significantly increased at all time points for the meropenem HD-treated males. Compared to females, marginal changes were observed in the plasma BAs in the meropenem LD- and HD-treated males at all sampling time points.

#### 3.6.2. Doripenem Hydrate Treatment Significantly Alters Plasma Metabolites during Treatment and the Metabolites Recover after Doripenem Administration Is Stopped

Significant changes (*p* < 0.05) in plasma metabolites of doripenem-treated animals were observed on days 7, 14, and 28 of treatment (Table 10 and Table 11). Among the several measured metabolites, the majority of the significantly changed ones belonged to amino acids, BAs, carbohydrates, lipids, energy metabolites, signal substances, nucleobases, and their derivatives. These also included key plasma biomarkers associated with the perturbed gut microbiota, that have been previously reported. Among the known plasma biomarkers, indole-3-propionic acid (IPA), 3IS, and HA were observed to be significantly reduced in plasma metabolomes from days 7, 14, and 28 [11,14,15].

Plasma metabolites on day 42 doripenem-recovery group in females (two weeks of no exposure), recovered after the doripenem administration was stopped. Although the metabolites from the LD group recovered fully, in the HD group, there were two metabolites that were not fully recovered. Two further metabolites, HA and TCA, were changed in the opposite direction in the HD group two-weeks post-doripenem cessation as compared to the doripenem-treated HD groups.

Of the 22 measured plasma BAs in the HD doripenem-treated female rats, 12 were significantly altered at a *p <* 0.05 (Table 11). The 2° BAs including DCA, HDCA, ωMCA, and isoLCA were significantly changed at all time points. The list also consisted mostly of the taurine-conjugated 1° and 2° BAs, including significant increases in TCA, TβMCA, and TCDCA and a reduction in TLCA, TUDCA, and TDCA in the HD-treated female animals. Plasma BAs in the **doripenem-recovery group** also recovered. As also noted in the females, a few BAs in the males, i.e., GLCA, TLCA, HDCA, and isoLCA, were increased in the HD-recovery group, while they were reduced during the treatment phase. In the LD group, only GLCA showed this overcompensation, whereas all other BAs in the LD-recovery group were not significantly different from the controls. This dose-response observation confirmed that this overcompensation is a real effect.

In the HD males, 40 out of the 294 measured plasma metabolites were significantly changed in samples on days 7, 14, and 28 (Table 12). These included metabolites belonging to amino acids, BAs, carbohydrates, lipids, energy metabolites, signal substances, nucleobases, vitamins, and their derivatives and a dose-response was observed. Furthermore, among the significantly changed plasma metabolites, some of the key biomarkers including 3IS and HA were observed to be reduced at all time points in the doripenem HD males. Plasma metabolites on day 42 from the doripenem-LD recovery males showed complete recovery without any over-compensation. In the doripenem-HD recovery group, 3 metabolites did not fully recover (tyrosine, glucose, and xylitol), whereas two other metabolites, 3-hydroxyindole and HA, showed an over-compensation as they were significantly changed in the opposite direction (i.e., were increased in the recovery group, while decreased under treatment).

Seventeen out of the twenty-two measured plasma BAs were significantly altered in the doripenem-treated males (*p <* 0.05). The list consisted of several 2° Bas, including ursodeoxycholic acid (UDCA), DCA, HDCA, and ωMCA, that are observed to be significantly reduced specifically in the HD-treated males (Table 13). Three 1° BAs, CA, chenodeoxycholic acid (CDCA), and α-muricholic acid (αMCA), were significantly reduced in the HD group. Additionally, many conjugated BAs including taurine- and glycine-conjugated 1° and 2° BAs were also significantly altered in the doripenem-treated males, specifically in the HD group.

Taurine-conjugated 1° BAs, TCA, TβCA, and TCDCA, were significantly increased whereas taurine-conjugated 2° BAs including TLCA, TUDCA, and TDCA were significantly decreased in the HD treatment group. Glycine-conjugated 1° BAs including glycocholic acid (GCA) and glycochenodeoxycholic acid (GCDCA) and 2° BAs, GLCA and GDCA, also were all observed to be significantly reduced, indicating a sex-specific response as these were not affected in this pattern in females. BA plasma levels in both doripenem-LD and -HD males recovered completely on day 42.

### 3.7. Comparative Analysis of the Metabolome Suggests Stronger Effects of Carbapenem Antibiotics on Feces than Plasma Metabolomes

There was a stronger effect of the antibiotic treatments on the fecal metabolites (35–59% significantly changed metabolites at *p*-value < 0.05) compared to plasma (only 10–21%) (Table 14). Upon comparing the percentage changes in the plasma and fecal metabolomes in the doripenem-recovery groups to previously changed levels upon doripenem treatment, it can be concluded that the recovery of altered metabolites was faster in males than in females. Additionally, it was noted that the feces metabolome recovered faster than the plasma, as more than 50% of the previously changed fecal metabolites reversed back to normal control values on day 42/43 (two weeks after no exposure to the antibiotic). Furthermore, with the false discovery rate at a *p*-value of 0.05 being 5%, the plasma metabolomes of the doripenem-HD recovery males and the doripenem-LD recovery females also suggested complete recovery. Comparing the percentages of significantly altered plasma and feces metabolites in the meropenem-treated animals, the number of changes in the fecal metabolomes was about 4–5 times higher than that in the plasma. This observation is similar in both the carbapenem treatment groups and in both sexes, indicating consistent antibiotic-class based effects on the metabolomes, and both antibiotics show a similar strength (Table 14C,D).

## 4. Discussion

**Figure 10:**
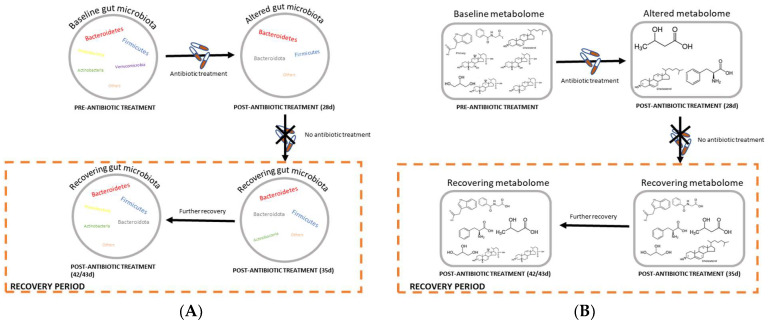
Schematic diagrams of the recovery processes of (**A**) gut or fecal microbiota and (**B**) plasma and feces metabolomes before and after doripenem antibiotic treatments. Baseline state indicates the original or ‘normal’ state of the gut microbiota or metabolomes prior to antibiotic administration, which becomes altered upon 28 days of antibiotic administration. Further, post-antibiotic treatment, a recovery or overcompensation takes place where the gut bacteria as well as the host metabolomes try to return to the ‘normal’ state.

We investigated how two carbapenem antibiotics, meropenem trihydrate and doripenem hydrate, alter the gut (fecal) microbiota composition and the derived plasma and fecal metabolites of male and female Wistar rats, as well as the recovery of these parameters one and two weeks after ending the administration of doripenem (Figure 10). The major findings of this research include the following: 1. Meropenem and doripenem induced significant changes in the gut (fecal) microbiota composition with antibiotic-specific changes. 2. There were significant changes in the fecal metabolic profiles, particularly including alterations in BA levels. 3. Additionally, there were significant changes in the levels of plasma metabolites previously identified as key markers of microbiota shifts, as well as BAs. 4. When animals were allowed a period of recovery following doripenem treatment, the changes in the 16S bacterial composition as well as fecal metabolites reversed after 2 two weeks. 5. A few plasma metabolites in the doripenem-HD recovery group showed incomplete recovery or overcompensation.

### 4.1. Microbiome Analysis

The effects of the two carbapenem treatments on the gut microbiome composition was different from other antibiotic classes, i.e., aminoglycosides, lincosamides, and fluoroquinolones, analyzed by Behr et al. 2017 and 2018, de Brujin et al. 2020, and Murali et al. 2021. As carbapenems exhibit a broad spectrum of antimicrobial activity against Gram-negative and Gram-positive bacteria, treatment groups consistently showed a reduced bacterial Shannon true diversity. Overall, in the control groups, the bacterial Shannon true or alpha diversity gradually increases with time from day 1 until day 42/43 of the study. This is consistent to Hoffmann et al. 2017, who also observed a higher Shannon diversity in older mice compared to younger ones [31]. The beta diversity analysis shows that samples of both treatment groups cluster together, with day 1 samples clearly separated from the rest of the sampling time points. This could be because of a yet incomplete elimination of less susceptible bacteria, with a subsequent further reduction in diversity at later time points which appears to be complete on day 7. Furthermore, the changes in 16S bacterial compositions on days 7 and 28 in the carbapenem groups were sex specific. A similar observation was noted in previously published studies using aminoglycosides, fluoroquinolones, lincosamides, and polymyxin E antibiotics. The factors responsible for these albeit subtle differences are still not very well established [12,13,32]. A few publications have highlighted potential reasons including the differences in hormone dynamics, host genetics, body mass index as some of the factors [33,34]. Overall, however, compared to the sex specific differences observed in rats, the species strain and environmental conditions of the animals have a more significant influence on its gut microbiota composition.

In the doripenem hydrate recovery group, the diversity analysis showed rapid recovery. The alpha diversity following one week of stopping the exposure in the doripenem-recovery LD and HD groups were comparable to the corresponding controls. Interestingly, in females of the doripenem-recovery LD group on day 42, the bacterial diversity appeared to be slightly higher than in the corresponding control group.

An inter-individual variability could be observed in the relative abundance analysis, particularly in the *Verrucomicrobiaceae*, as seen in previous investigations, where this high variability in *Verrucomicrobiaceae* in the control animals was already noted. To assess the long-term variability in controls, further studies would be helpful and needed to attain a historic range of the bacterial families for a better interpretation of results (similar to historical control data in clinical or histopathology). Consistent with the diversity analysis, the relative abundance analysis also showed a distinct bacterial composition on day 1 of the antibiotic treatment groups compared to the rest of the sampling time points. The meropenem and doripenem HD groups showed a more pronounced reduction in bacterial diversity than the LD group in both sexes, resulting in increased relative abundances of *Enterococcaceae*, *Anaeroplasmataceae*, *Erysipelotrichaceae*, and *Peptostreptococcaceae* compared to the controls indicating that these are highly likely to be resistant to carbapenem antibiotics. In both the meropenem and doripenem LD groups, the bacterial abundances changed with the time of exposure to the respective antibiotics, which indicates that the bacteria that are resistant to the activity of the respective antibiotics start to colonize and fill niches that are made available by the selected antibiotic, and hence, on day 1, this overgrowth with the above mentioned four strains was less evident than the succeeding sampling time points [35]. Furthermore, samples from recovery time points, that is, one and two weeks after the doripenem treatment was stopped, showed comparable bacterial abundances (observed in both relative and differential abundance analyses) as controls, which again demonstrates that the gut microbiota repopulation one week after antibiotic exposure was stopped. Similar observations were noted upon the cessation of other antibiotics such as ciprofloxacin and vancomycin in humans where gut bacterial communities began to return to their initial state after one and two weeks post-antibiotic cessation, respectively [36,37].

### 4.2. Metabolome Analysis

#### 4.2.1. Both Carbapenems Altered Gut-Derived BAs and Plasma Biomarkers

The PCA analysis of the fecal metabolome showed a strong effect of meropenem. From the 620 measured fecal metabolites, as many as 47% and 43% metabolites were significant in the HD males and females, while in the LD groups, these values were 33% and 32%, respectively. Fecal 1° BAs including taurine-conjugated 1° BAs significantly increased in fecal metabolomes from all the time points (days 7, 14, and 28) of all the meropenem groups. Strong increases in CA suggest the lack of utilization of the 1° BAs for further bacterial dehydroxylation reactions to produce corresponding 2° or 3° BAs. The accumulation of taurine-conjugated 1° BAs, specifically TCAs, also indicates reduced deconjugation activity of the remaining intestinal bacterial communities. This observation is in line with the antibiotics from other classes, such as the lincosamide antibiotics (clindamycin and lincomycin) and vancomycin [11,12,15]. Furthermore, TCA was observed to be actively transported from the intestine, and it was shown that TCA uptake is increased in the ileal epithelium of Germ-free rats [38]. Therefore, although meropenem trihydrate, vancomycin, lincomycin, and clindamycin are antibiotics with a different spectrum of activity, their interference with bacterial BA metabolism appears to be rather similar.

Strong reductions in 2° BAs, specifically in DCA, HDCA, ωMCA, and LCA, were observed in the meropenem treatment groups. This substantial reduction in these 2° BAs confirms the lack of an ability to convert 1° BAs into 2° BAs by gut bacteria. Gut bacteria are known to deconjugate the taurine- or glycine-conjugated 1° BAs and further dehydroxylate them to produce respective 2° BAs. The majority of gut bacteria are anaerobes, and mainly anaerobic bacteria possess a 7α- dehydroxylation activity to convert 1° BAs into 2° BAs [39]. Consequently, it can be assumed that meropenem trihydrate significantly reduced those essential gut (or fecal) bacteria that are responsible for these reactions. The PCA analyses of the plasma metabolome showed only some alterations compared to the controls. Among the 294 measured plasma metabolites, only about 15% and 13% were significantly changed in the samples from day 28 of meropenem HD-treated males and females, respectively. In the LD group, these values were 12% and 8.5% in male and female rats, respectively. These values are above the false discovery rate but far less compared to the changes in the fecal metabolites.

Some of the previously established plasma biomarkers that are known to be associated with perturbations in the gut microbiome include HA, indole derivatives (incl. IAA, IPA, and 3-indoxylsulfate), and glycerol [11]. From these, a significant reduction in 3-IS and HA was observed in both sexes of the meropenem treatment groups, confirming their usefulness as general indicators of disturbed gut microbiome composition and metabolic functionality. Surprisingly, one of the indole derivatives, IAA, which was previously found to be significantly reduced during treatments with antibiotics belonging to the aminoglycosides, lincosamides, and fluoroquinolones classes [11,12,32,32], was significantly increased in the meropenem treatment group, specifically in the HD group (Table S5). This observation suggests a meropenem-specific effect on this specific plasma key metabolite in both sexes. A potential cause of this significant increase of this otherwise reduced plasma biomarker could be the loss of essential gut bacteria that normally utilize these metabolites.

Plasma BAs at all sampling time points showed significant alterations compared to the controls, including significant reductions in 2° BAs. Plasma 2° BAs including DCA, LCA, HDC, ωMCA, and isoLCA were all very low in the HD meropenem groups, indicating either reduced deconjugation reactions by gut bacteria consistent with the fecal BAs or a reduced conversion of deconjugated 1° into 2° BAs. Similarly, almost all the conjugated 2° BAs were also significantly reduced in the plasma of the meropenem groups. Thus, meropenem is observed to not only alter the fecal 16S composition but also consequently the fecal metabolome and the plasma metabolome, hereby indicating a potential influence on gut metabolic functionality. This is in line with previous observations from other antibiotics belonging to aminoglycosides, fluoroquinolones, and lincosamides, as published by de Bruijn et al. 2020 and Murali et al. 2021 [1,12]. Finally, to evaluate the observed effects of meropenem on the different metabolites including BAs as a potential systemic effect, we investigated if any form of liver toxicity could have been involved. We used the MetaMapTox database to compare the plasma metabolome of meropenem HD-treated animals with the pre-defined patterns for liver toxicity, as previously published [40,41,42]. This comparison did not indicate any association with liver toxicity. Similar to meropenem, samples from rats treated with doripenem had significantly altered fecal metabolites consequent to the changes in the gut 16S composition. The PCA analysis showed strong changes in the treatment groups in both sexes. From the 620 measured fecal metabolites, about 50% and 54.5% were significantly changed in the HD-treated male and female rats, respectively, and about 40% and 37% in the LD, respectively.

The 1° BAs and several conjugated 1° BAs were significantly increased in feces samples from all the animals treated with doripenem, specifically in the HD group. Amongst these, TCA was strongly increased in the feces of these samples in both sexes. Such high accumulation of 1° as well as conjugated 1° BAs indicates a reduction in gut bacteria that have the ability to perform the conversion of 1° as well as conjugated 1° BAs into their respective 2° and 3° forms via dehydroxylation and deconjugation reactions, as discussed previously for meropenem. Furthermore, 2° BAs including DCA, LCA, HDCA, ωMCA, and isoLCA were significantly reduced in all the samples from the doripenem treatment group relative to the controls, confirming the lack of bacterial deconjugation and 7α-dehydroxylation activities. Furthermore, conjugated 2° BAs were also consistently reduced. This could be the result of the reduction in gut bacteria possessing special enzymes necessary for deconjugation, which also explains that further conjugation of these 2° BAs in the liver is highly unlikely to take place. Unique bacterial enzymes called the bile salt hydrolases (BSH) are known to transform 1° into 2° BAs by cleaving the peptide linkage between the amino acid and the primary bile acid. Moreover, and in contrast to males, females treated with the doripenem antibiotic showed a significant decrease in glycine-conjugated 2° BAs, GLCA, and GDCA, highlighting the sex-specific effects of the antibiotic on these fecal BAs.

Similar to meropenem, far fewer changes in the plasma metabolome were observed for the doripenem treatment. Amongst the 294 measured blood plasma metabolites, about 20% and 19% were significantly changed in HD-treated male and female rats, respectively, and about 15% and 11% in the LD groups, respectively. Key plasma biomarkers including 3IS and HA were consistently reduced in all treatment groups. In females, IPA was significantly reduced in all the plasma metabolomes from the doripenem HD treatment. Significant reduction in these biomarkers is known to be associated with gut-dysbiosis based on the observations published by Behr et al. 2018, where a temporary shift in the gut microbiota was induced as well as the associated fecal metabolites using antibiotic of different classes including aminoglycosides, lincosamides, and fluoroquinolones [11,14,15]. This observation is similar to that of the meropenem treatment, strongly indicating a carbapenem class-specific response.

Several conjugated 1° BAs were significantly increased in the plasma of the doripenem treatment group in both sexes. This substantiates the observation in the fecal metabolome upon the antibiotic treatment and indicates impairment of bacterial deconjugation activity and further accumulation of these conjugated BAs in the system. In males, additionally, 1° BAs, CA, and CDCA as well as some of the glycine-conjugated 1° BA were significantly reduced specifically in the HD group. Reduced levels of CDCA in the plasma of the HD group males in the absence of any significant change in CDCA in the feces suggests a possible increase in the conjugation activity of CDCA to TCDCA. This reduction in CA and CDCA in the plasma of antibiotic-treated rats is not unique as it was also observed upon roxithromycin treatment [12,32,32]. Furthermore, a significant reduction in 2° BAs including DCA, HDCA, and ωMCA could be consistently observed in all HD treatment groups. The 2° BAs isoLCA and UDCA were significantly reduced in females and males treated with doripenem HD, respectively. This decrease suggests an impairment in the bacterial dehydroxylation and deconjugation activity. In line with this observation, conjugated 2° BAs were also significantly reduced indicating the lack of 2° BAs to further undergo conjugation reaction in the liver.

#### 4.2.2. Doripenem Hydrate Showed Indication of Recovery of Significantly Changed Metabolites 2-Weeks Post Doripenem Cessation

In females, the fecal metabolome from one-week post-doripenem high-dose cessation still showed significant changes in almost 57% of the previously changed BAs. The majority of these BAs changed in the opposite direction compared to the antibiotic-treated group. Similarly, in the LD group, fecal doripenem recovery metabolome from one-week post-antibiotic cessation (day 35) still showed significant changes in 36% of the previously changed BAs, out of which the majority of the changes were in the opposite direction. These observations suggest that a mechanism of overcompensation occurred in the fecal metabolome one week after doripenem treatment was stopped. This could be a potential feedback mechanism of the body in order to attain metabolic homeostasis one-week post-doripenem cessation. This can be further observed in the fecal metabolome from two-weeks post-doripenem cessation, as the changes in these samples are lower than on day 35, therefore, suggesting advancement towards full recovery. In males, the recovery seems to be faster than in females. This observation again indicates subtle differences between the sexes extending beyond the microbiome to the associated metabolites. During antibiotic administration, the effects on the fecal BA levels was also stronger in females, and this may explain why recovery in females was slower than in males.

The recovery of the plasma metabolome was analyzed with samples from two-weeks post-doripenem cessation. There was full recovery of the plasma metabolome of the LD group for males and females. For one metabolite (HA) there was overcompensation in the HD group females, i.e., the direction of change was reversed in the doripenem-recovery groups compared to control group. Interestingly, there were four bile acids for which an overcompensation was noted. In the HD group males, two metabolites (3-hydroxyindol and HA) were still increased despite the two weeks of recovery time, whereas none of the BAs were significantly changed two weeks after doripenem treatment was stopped in both LD and HD males. Similarly, for the HD group females, a longer duration than two weeks would ideally be needed for a full recovery. In males, there were three metabolites, tyrosine, glucose, and xylitol, and two metabolites in females, citrate and 3-methoxytyrosine, which had not fully recovered. These metabolites that remained significantly altered two-weeks post-doripenem antibiotic cessation could either mean that they need more time to go back to their normal or baseline state or that they could be potential markers of disturbance, which may help provide therapeutic targets for microbiota recovery following antibiotic treatment [43]. Further, in contrast to females, all bile acids had returned to levels comparable to controls.

### 4.3. Associations between the Changes in Gut Microbiota and Associated Metabolites

Based on our observations, we hypothesize possible functions of gut bacteria, specifically with respect to BA metabolism. Several published studies on the species that have 7α-dehydroxylating activity (resulting in secondary bile acids) have focused *on Clostridium* sp, *Bifidobacterium* sp., *Bacteroides* sp., and *Bifidobacterium* sp. [44], whereas many other dominant facultative anaerobes of the intestinal bacteria have been less explored regarding their abilities to perform dehydroxylation and deconjugation reactions. Our research indicated other bacterial families also to be involved in BA pool regulation. Drastic reduction in two specific bacterial families, *Bacteroidaceae* and *Prevotellaceae* (belonging to Proteobacteria and Bacteroidetes phyla, respectively), were observed in all antibiotic treatment groups compared to controls for both sexes. As a significant reduction in the 2° BAs (in both plasma and feces) was also found in these antibiotic treatment groups, this suggests a role of these two bacterial families, which are normally rather abundant, in bacterial deconjugation and dehydroxylation reactions transforming 1° into 2° BAs. Moreover, increases in the relative abundances of the *Enterococcaceae* family were consistently observed in both the meropenem and doripenem treatment groups suggesting that they do not contribute to BA-related metabolism. Other rather weakly reduced bacterial families in the meropenem LD and HD treatment groups were observed to be *Lachnospiraceae, Ruminococcaceae*, and *Porphyromonadaceae* in both sexes of antibiotic treatments, also indicating a potential bile acid transformation activity of the two families [39]. Overall, all these changed bacterial families recovered to levels comparable to the controls in microbiota from one and two weeks after doripenem treatment was stopped, which was also consequently observed in the metabolomes (both blood plasma and feces), further validating these associations between the altered gut microbiota and its associated metabolites (specifically, BAs).

## 5. Conclusions

In this 28-day oral feeding study on Wister rats, we observed that broad spectrum carbapenem antibiotics induced significant changes in the gut microbiota and related fecal metabolome profiles, specifically concerning the BA pool. Additionally, there was an efficient recovery and reconstitution in the 16S bacterial composition as well as in fecal metabolites following alterations induced by doripenem. In plasma metabolomes, however, specifically for animals administered doripenem HD, the recovery was slower and there were indications of overcompensation of several metabolites that either were overly increased or decreased from their perturbation levels during the treatment itself, suggesting an ongoing recovery process. The plasma metabolites were consistent with changes in metabolites previously established as key biomarkers of microbiota shifts including BAs, but a unique observation was that one of the key metabolites, indole-acetic acid (IAA), increased following meropenem treatment but not doripenem or any other antibiotics tested under the same experimental conditions. While further studies are needed to understand whether there are any adverse or toxicological consequences to host health due to the measured changes in the metabolites, and how this might be influenced by co-administration with other drugs, the results suggest a relatively efficient recovery of the gut bacterial communities as well as their associated metabolic/functional output. The effective spontaneous recovery suggests that there may be little need for therapeutic interventions for restoration of the microbiome. Furthermore, due to apparent inter-species differences in the gut microbiota composition between rodents and humans, these results may not be directly applied or translated to human beings. This study also challenges the use/requirement for probiotics to restore gut communities. Whether overcompensated metabolites will return to their normal state, although likely, would require an extension beyond two weeks after antibiotic cessation.

## Figures and Tables

**Table 1:** Compounds used, dose levels, and form of preparation. All compounds were administered orally using gavage.

Treatment	Low Dose (mg/kg bw/d ^a^)	High Dose (mg/kg bw/d ^a^)
Meropenem trihydrate	100	300
Doripenem hydrate	100	1000

^a^ mg/kg bw/d means milligrams per kilogram body weight of rat per day.

**Table 2:** Meropenem-induced BA fold changes in feces of female (f) Wistar rats (N = 5 per group) dosed with 100 (LD) and 300 (HD) mg/kg bw/day observed on days 7, 14, and 28 (f7, f14, and f28). Statistically significant changes (Welch *t*-test; *p*-value < 0.05) are shown where red boxes mean a significant increase, yellow boxes mean a significant decrease, and white boxes indicate an insignificant change in the respective fecal BAs.

		Meropenem Trihydrate HD			Meropenem Trihydrate LD		
Metabolite	Class	f7	f14	f28	f7	f14	f28
Deoxycholic acid	2° BA *	0.01	0.01	0.06	0.09	0.16	0.17
Cholic acid	1° BA *	10.78	9.57	7.33	17.48	1.14	0.91
Lithocholic acid	2° BA	0.02	0.03	0.02	0.08	0.15	0.15
Taurocholic acid sodium salt	Taurine-conjugated 1° BA	4.70	2.04	3.85	3.41	1.87	1.14
Taurolithocholic Acid	Taurine-conjugated 2° BA	0.04	0.12	0.15	0.41	0.61	0.78
Taurocholic Acid 3-sulfate	Taurine-conjugated 1° BA	185.39	197.22	280.09	40.98	81.49	6.10
Tauro-b-muricholic Acid	Taurine-conjugated 1° BA	3.51	2.01	3.42	2.14	1.30	1.10
Taurochenodeoxycholate	Taurine-conjugated 1° BA	2.08	1.43	2.60	1.71	1.56	1.17
Hyodeoxycholic acid	2° BA	0.01	0.01	0.01	0.02	0.01	0.04
o-Muricholic Acid	2° BA	0.19	0.04	0.03	0.60	0.12	0.13
isoLCA	2° BA	0.01	0.01	0.01	0.08	0.28	0.31

* 1° BA means primary bile acids, and 2° BA means secondary bile acids.

**Table 3:** Meropenem-induced BA fold changes in feces of male Wistar rats (N = 5 per group) dosed with 100 (LD) and 300 (HD) mg/kg bw/day observed on days 7, 14, and 28 (m7, m14, and m28). Statistically significant changes (Welch *t*-test; *p*-value < 0.05) are shown where red boxes mean an increase, yellow boxes mean a significant decrease, and white boxes indicate an insignificant change in the respective fecal BAs.

		Meropenem Trihydrate HD			Meropenem Trihydrate LD		
Metabolite	Class	m7	m14	m28	m7	m14	m28
Deoxycholic acid	2° BA	0.01	0.01	0.00	0.20	0.21	0.24
Cholic acid	1° BA	13.78	22.75	33.78	4.61	5.80	7.61
Lithocholic acid	2° BA	0.02	0.03	0.02	0.13	0.10	0.08
Taurocholic acid sodium salt	Taurine-conjugated 1° BA	4.49	110.41	12.63	8.93	19.40	0.33
Taurocholic Acid 3-sulfate	Taurine-conjugated 1° BA	15.24	921.65	113.19	83.21	759.51	0.13
Tauro-b-muricholic Acid	Taurine-conjugated 1° BA	3.50	26.50	12.70	10.01	12.34	2.03
a-Muricholic Acid	1° BA	23.88	2.53	2.28	18.70	5.85	2.13
b-Muricholic Acid	1° BA	8.16	2.43	2.13	5.03	1.73	3.48
Hyodeoxycholic acid	2° BA	0.02	0.00	0.00	0.01	0.00	0.01
o-Muricholic Acid	2° BA	0.35	0.05	0.05	0.14	0.15	0.07
isoLCA	2° BA	0.01	0.01	0.01	0.14	0.19	0.14

**Table 4:** Doripenem-induced BA fold changes in feces of female Wistar rats (N = 5 per group) dosed with 100 (LD) and 1000 (HD) mg/kg bw/day observed on days 7, 14, and 28 (f7, f14, and f28) and on days 35 and 42 (f35 and f42) for the recovery group. Statistically significant changes (Welch *t*-test; *p*-value < 0.05) are shown where red boxes mean an increase, yellow boxes mean a significant decrease, and white boxes indicate an insignificant change in the respective fecal BAs.

		Doripenem HD			Doripenem HD Recovery		Doripenem LD			Doripenem LD Recovery	
Metabolite	Class	f7	f14	f28	f35	f42	f7	f14	f28	f35	f43
Deoxycholic acid	2° BA	0	0	0	0.85	0.5	0.01	0	0.03	1.04	0.68
Cholic acid	1° BA	3.44	2.81	5.6	1.15	1.08	15.5	15.76	15.61	1.23	0.88
Lithocholic acid	2° BA	0.01	0.01	0.01	2.23	1.13	0.02	0.01	0.03	1.68	1.11
Taurocholic acid sodium salt	Taurine-conjugated 1° BA	8.1	6.65	5.78	0.09	0.37	3.83	2.12	2.46	0.28	0.85
Glycolithocholic Acid	Glycine-conjugated 2° BA	0.2	0.13	0.06	6.89	5.76	0.56	0.09	0.29	5.39	0.63
Taurolithocholic Acid	Taurine-conjugated 2° BA	0.03	0.02	0.01	0.9	2.66	0.2	0.03	0.4	1.25	0.89
Taurocholic Acid 3-sulfate	Taurine-conjugated 1° BA	395.06	302.06	28.52	1.03	0.91	139.94	156.93	16.82	2.13	1.32
Tauro-b-muricholic Acid	Taurine-conjugated 1° BA	6.8	4.47	2.27	0.39	0.82	2.02	1.59	1.73	0.82	0.85
Taurochenodeoxycholate	Taurine-conjugated 1° BA	1.65	1.94	1.95	0.92	0.86	1.63	1.18	1.87	0.98	0.77
Taurodeoxycholate	Taurine-conjugated 2° BA	0.54	0.46	0.49	0.66	0.64	0.55	0.48	0.46	0.66	0.69
Glycodeoxycholate	Glycine-conjugated 2° BA	0.46	0.33	0.35	1.48	1.28	0.45	0.26	0.39	1.05	0.93
Hyodeoxycholic acid	2° BA	0	0	0	6.9	3	0.03	0	0.01	1.82	0.49
o-Muricholic Acid	2° BA	0.01	0	0.01	0.74	0.08	0.28	0.08	0.08	1.3	1.25
isoLCA	2° BA	0	0	0.01	3.18	1.5	0.01	0	0.02	1.63	1.2

**Table 5:** Doripenem-induced BA fold changes in feces of male Wistar rats (N = 5 per group) dosed with 100 (LD) and 1000 (HD) mg/kg bw/day observed on days 7, 14, 28 (m7, m14 and m28) and on days 35 and 42 (m35 and m43) for the doripenem-recovery group. Statistically significant changes (Welch *t*-test; *p*-value < 0.05) are shown where red boxes mean an increase, yellow boxes mean a significant decrease, and white boxes indicate an insignificant change in the respective fecal BAs.

		Doripenem HD			Doripenem HD Recovery		Doripenem LD			Doripenem LD Recovery	
Metabolite	Class	m7	m14	m28	m35	m43	m7	m14	m28	m35	m43
Deoxycholic acid	2° BA	0.01	0	0.01	0.77	1.15	0.01	0	0	0.91	0.78
Cholic acid	1° BA	14.31	26.92	5.47	0.98	0.87	15.34	20.97	14.38	1	0.47
Lithocholic acid	2° BA	0.02	0.02	0.02	1.09	1.07	0.02	0.02	0.02	1.07	0.74
Taurocholic acid sodium salt	Taurine-conjugated 1° BA	16.62	73.02	11.51	0.5	0.2	6.14	15.13	4.85	0.76	0.38
Taurocholic Acid 3-sulfate	Taurine-conjugated 1° BA	142.02	1738.7	129.59	2.24	0.37	20.83	85.11	43.38	1.52	0.12
Tauro-b-muricholic Acid	Taurine-conjugated 1° BA	20.19	148.81	40.8	0.7	0.67	4.03	6.14	2.65	0.64	0.45
Hyodeoxycholic acid	2° BA	0.01	0	0.01	0.23	0.84	0.01	0	0.01	0.58	0.45
o-Muricholic Acid	2° BA	0.08	0	0	2	1.32	0.14	0.03	0.02	1.05	0.6
isoLCA	2° BA	0.01	0	0	0.78	0.89	0	0	0.01	0.96	0.55

**Table 6:** Meropenem-induced plasma metabolite fold changes in female (f) Wistar rats (N = 5 per group) dosed with 100 (LD) and 300 (HD) mg/kg bw/day observed on 7, 14, and 28 days (f7, f14, and f28). Statistically significant changes (Welch *t*-test; *p*-value < 0.05) are shown where red boxes mean a significant increase in the respective plasma metabolites and yellow boxes mean a significant reduction compared to control levels.

		Meropenem Trihydrate HD			Meropenem Trihydrate LD		
Metabolite	Class	f7	f14	f28	f7	f14	f28
Threonine	Amino acids	0.87	0.80	0.84	0.93	0.99	1.05
Glycine	Amino acids	0.92	0.89	0.87	0.86	0.87	0.97
Threonine	Amino acids	0.83	0.82	0.84	0.99	0.94	1.03
Cysteine	Amino acids	0.85	1.03	0.93	0.92	1.11	1.08
3-Hydroxyindole	Amino acids-related	0.11	0.10	0.24	0.86	0.45	0.97
Indole-3-acetic acid	Amino acids-related	5.60	4.82	5.66	1.68	2.44	1.94
3-Indoxylsulfate	Amino acids-related	0.10	0.15	0.27	0.87	0.59	1.10
Deoxyribonucleic acids, total	Carbohydrates and related	0.81	0.93	1.06	0.79	0.91	0.73
Xylitol	Carbohydrates and related	1.64	1.52	1.54	0.84	0.76	1.41
3-Hydroxybutyrate	Energy metabolism and related	1.30	1.23	0.97	1.13	1.04	0.96
3-Hydroxybutyrate	Energy metabolism and related	1.41	1.32	1.15	1.29	1.12	1.02
3-Methoxytyrosine	Hormones, signal substances and related	1.43	1.44	1.44	1.18	1.20	1.24
17-Hydroxypregnenolone	Hormones, signal substances and related	0.02	0.01	0.03	0.02	0.03	0.02
Hippuric acid	Miscellaneous	0.24	0.24	0.43	0.48	0.53	0.99
Cytosine	Nucleobases and related	0.78	0.77	1.08	0.76	0.78	0.78

**Table 7:** Meropenem-induced plasma BA fold changes in female (f) Wistar rats (N = 5 per group) dosed with 100 (LD) and 300 (HD) mg/kg bw/day observed on 7, 14, and 28 days (f7, f14, and f28). Statistically significant changes (Welch *t*-test; *p*-value < 0.05) are shown where red boxes mean a significant increase in the respective plasma BAs and yellow boxes mean a significant reduction compared to control levels.

		Meropenem Trihydrate HD			Meropenem Trihydrate LD		
Metabolite	Class	f7	f14	f28	f7	f14	f28
Deoxycholic acid	2° BA	0.04	0.04	0.24	0.49	0.82	1.03
Lithocholic acid	2° BA	0.01	0.02	0.05	1.04	0.72	1.82
Taurolithocholic Acid	Taurine-conjugated 2° BA	0.01	0.03	0.07	0.07	0.19	0.26
Tauroursodeoxycholic Acid	Taurine-conjugated 2° BA	3.49	1.83	6.66	5.30	2.14	8.83
Taurodeoxycholate	Taurine-conjugated 2° BA	0.00	0.00	0.04	0.08	0.14	0.32
Glycocholic acid	Glycine-conjugated 1° BA	4.30	1.82	5.16	2.28	3.68	3.61
Glycochenodeoxycholic acid	Glycine-conjugated 1° BA	8.51	2.62	13.20	10.69	16.07	12.32
Glycodeoxycholate	Glycine-conjugated 2° BA	0.05	0.14	0.39	0.57	2.94	3.19
Glycoursodeoxycholic acid	Glycine-conjugated 2° BA	22.25	11.61	35.10	10.72	32.78	24.98
Hyodeoxycholic acid	2° BA	0.03	0.03	0.03	0.14	0.05	0.18
o-Muricholic Acid	2° BA	0.20	0.16	0.29	0.88	0.23	1.21
isoLCA	2° BA	0.06	0.48	0.34	0.07	0.35	0.72

**Table 8:** Meropenem-induced plasma metabolite fold changes in male (m) Wistar rats (N = 5 per group) dosed with 100 (LD) and 300 (HD) mg/kg bw/day observed on 7, 14, and 28 days (m7, m14, and m28). Statistically significant changes (Welch *t*-test; *p*-value < 0.05) are shown where red boxes mean a significant increase in the respective plasma metabolites and yellow boxes mean a significant reduction compared to control levels.

		Meropenem Trihydrate HD			Meropenem Trihydrate LD		
Metabolite	Class	m7	m14	m28	m7	m14	m28
Threonine	Amino acids	0.86	0.82	0.75	0.90	1.04	0.97
Threonine	Amino acids	0.93	0.78	0.75	0.89	0.98	0.92
Methionine	Amino acids	0.97	0.88	0.88	1.02	0.98	1.05
3-Indoxylsulfate	Amino acids-related	0.11	0.11	0.09	0.74	0.63	0.46
Indole-3-acetic acid	Amino acids-related	3.02	6.51	9.41	5.03	4.17	6.45
3-Hydroxyindole	Amino acids-related	0.12	0.11	0.14	1.07	0.64	0.48
Lysophosphatidylcholine (C18:2)	Complex lipids, fatty acids and related	1.07	0.96	1.14	0.97	0.96	1.17
Citrate	Energy metabolism and related	1.09	1.17	1.17	1.04	1.11	1.28
Cortisol	Hormones, signal substances and related	3.36	2.72	4.95	1.43	1.69	2.21
Corticosterone	Hormones, signal substances and related	2.68	1.64	1.65	2.90	2.15	2.34
18-Hydroxy-11-deoxycorticosterone	Hormones, signal substances and related	2.16	1.52	1.27	2.44	2.61	2.40
Hippuric acid	Miscellaneous	0.52	0.33	0.53	0.27	0.41	0.32

**Table 9:** Meropenem-induced blood plasma BA fold changes in male (m) Wistar rats. Metabolite fold changes in plasma BAs of male Wistar rats (N = 5 per group) dosed with meropenem (100 (LD) and 300 (HD) mg/kg bw/day) observed on 7, 14, and 28 days (m7, m14, and m28). Statistically significant changes (Welch *t*-test; *p*-value < 0.05) are shown where red boxes mean a significant increase in the respective plasma BAs and yellow boxes mean a significant reduction compared to control levels.

		Meropenem Trihydrate HD			Meropenem Trihydrate LD		
Metabolite	Class	m7	m14	m28	m7	m14	m28
Ursodeoxycholic acid	2° BA	1.54	8.42	3.22	2.41	4.27	2.23
Deoxycholic acid	2° BA	0.01	NA	0.02	0.46	1.84	0.29
Lithocholic acid	2° BA	0.02	0.14	0.02	0.14	0.59	0.07
Taurolithocholic Acid	Taurine-conjugated 2° BA	0.05	0.08	0.08	0.16	0.16	0.31
Taurocholic Acid 3-sulfate	Taurine-conjugated 1° BA	0.22	1.10	0.31	0.65	0.30	0.34
Tauro-b-muricholic Acid	Taurine-conjugated 1° BA	1.73	4.06	1.96	0.95	1.03	1.10
Tauroursodeoxycholic Acid	Taurine-conjugated 2° BA	1.25	7.42	5.90	1.44	1.18	0.63
b-Muricholic Acid	1° BA	1.45	3.56	2.23	1.51	1.65	1.88
Taurochenodeoxycholate	Taurine-conjugated 1° BA	2.42	3.53	2.05	1.17	0.68	1.22
Taurodeoxycholate	Taurine-conjugated 2° BA	0.02	0.00	0.03	0.10	0.21	0.10
Glycodeoxycholate	Glycine-conjugated 2° BA	0.01	NA	0.01	0.64	3.02	0.09
Hyodeoxycholic acid	2° BA	0.01	0.03	0.01	0.03	0.01	0.00
o-Muricholic Acid	2° BA	0.11	1.23	0.07	0.12	0.14	0.09
isoLCA	2° BA	0.26	2.59	0.12	0.29	0.44	0.35

**Table 10:** Doripenem-induced plasma metabolite fold changes in female (f) Wistar rats (N = 5 per group) dosed with 100 (LD) and 1000 (HD) mg/kg bw/day observed on days 7, 14, and 28 (f7, f14, and f28) and on day 42 (f42) from the doripenem-recovery group. Statistically significant changes (Welch *t*-test; *p*-value < 0.05) are shown where red boxes mean a significant increase, yellow boxes mean a significant decrease, and white boxes indicate an insignificant change in the respective plasma metabolites compared to control group.

		Doripenem HD			Doripenem HD Recovery	Doripenem LD			Doripenem LD Recovery
Metabolite	Class	f7	f14	f28	f42	f7	f14	f28	f42
Tyrosine	Amino acids	1.11	0.79	0.75	0.84	0.83	0.83	0.97	1.12
Threonine	Amino acids	0.83	0.77	0.80	1.06	0.92	0.90	0.90	1.02
Tyrosine	Amino acids	0.88	0.82	0.80	0.84	0.86	0.86	0.95	0.98
trans-4-Hydroxyproline	Amino acids-related	0.90	0.81	0.89	1.07	1.11	0.93	0.93	1.14
3-Hydroxyindole	Amino acids-related	0.09	0.09	0.06	0.79	0.31	0.55	0.63	0.72
Ketoleucine	Amino acids-related	0.67	0.76	0.71	0.79	0.81	0.74	0.88	0.96
Indole-3-propionic acid	Amino acids-related	0.85	0.87	0.66	0.77	1.74	0.93	0.93	0.90
3-Indoxylsulfate	Amino acids-related	0.06	0.04	0.03	0.96	0.24	0.37	0.66	0.93
Deoxyribonucleic acids, total	Carbohydrates and related	0.76	0.92	0.72	0.99	0.79	0.80	0.78	0.91
Ceramide (d18:1,C24:1)	Complex lipids, fatty acids and related	0.80	0.71	0.74	1.27	1.20	1.29	1.37	1.06
Ceramide (d18:1,C24:0)	Complex lipids, fatty acids and related	0.73	0.68	0.62	1.25	1.02	1.25	1.19	1.09
Citrate	Energy metabolism and related	1.09	1.26	1.26	1.09	1.05	1.10	1.12	0.98
2-Hydroxybutyrate	Energy metabolism and related	1.55	1.60	2.09	1.17	1.37	1.52	1.14	0.73
3-Methoxytyrosine	Hormones, signal substances and related	1.10	1.22	1.2	1.26	1.38	1.41	1.05	1.10
17-Hydroxypregnenolone	Hormones, signal substances and related	0.01	0.01	0.02	0.83	0.01	0.04	0.53	1.25
beta-Sitosterol, total	Miscellaneous	0.62	0.74	0.74	1.12	1.01	1.07	0.80	1.13
Hippuric acid	Miscellaneous	0.23	0.25	0.48	1.58	0.39	0.32	0.52	1.00
Cytosine	Nucleobases and related	0.86	0.77	0.71	0.88	0.89	0.64	0.83	0.75
Allantoin	Nucleobases and related	0.75	0.89	0.86	1.01	0.73	0.82	1.01	0.97
Cholesterol, total		0.84	0.99	0.69	1.32	1.04	1.30	0.83	1.15

**Table 11:** Doripenem-induced plasma BA fold changes in female (f) Wistar rats (N = 5 per group) dosed with 100 (LD) and 1000 (HD) mg/kg bw/day observed on days 7, 14, and 28 (f7, f14, and f28) and on day 42 (f42) for the doripenem-recovery group. Statistically significant changes (Welch *t*-test; *p*-value < 0.05) are shown where red boxes mean a significant increase, yellow boxes mean a significant decrease, and white boxes indicate an insignificant change in the respective plasma BAs compared to control groups.

		Doripenem HD			Doripenem HD Recovery	Doripenem LD			Doripenem LD Recovery
Metabolite	Class	f7	f14	f28	f42	f7	f14	f28	f42
Deoxycholic acid	2° BA	0.02	0.02	0.03	3.41	0.02	0.06	0.32	2.57
Taurocholic acid sodium salt	Taurine-conjugated 1° BA	0.99	3.46	1.88	0.44	0.68	0.66	0.76	0.49
Glycolithocholic Acid	Glycine-conjugated 2° BA	0.11	0.12	0.33	9.27	0.04	0.37	1.26	13.31
Taurolithocholic Acid	Taurine-conjugated 2° BA	0.01	0.01	0.04	3.43	0.01	0.03	0.18	1.32
Taurocholic Acid 3-sulfate	Taurine-conjugated 1° BA	1.68	2.09	2.41	0.98	1.26	1.83	2.24	1.62
Tauro-b-muricholic Acid	Taurine-conjugated 1° BA	1.24	6.05	2.74	0.78	1.48	1.52	1.22	1.21
Tauroursodeoxycholic Acid	Taurine-conjugated 2° BA	14.68	7.45	17.31	3.88	5.43	2.66	11.53	0.97
Taurochenodeoxycholate	Taurine-conjugated 1° BA	1.29	2.94	1.91	1.30	1.38	1.33	1.28	1.12
Taurodeoxycholate	Taurine-conjugated 2° BA	0.00	0.00	0.01	0.65	0.00	0.00	0.05	0.68
Hyodeoxycholic acid	2° BA	0.02	0.01	0.01	19.38	0.05	0.01	0.02	2.55
o-Muricholic Acid	2° BA	0.11	0.08	0.14	0.66	0.16	0.13	0.45	1.98
isoLCA	2° BA	0.08	0.27	0.23	3.90	0.07	0.10	0.13	3.06

**Table 12:** Doripenem-induced plasma metabolite fold changes in male (m) Wistar rats (N = 5 per group) dosed with 100 (LD) and 1000 (HD) mg/kg bw/day observed on days 7, 14, and 28 (m7, m14, and m28) and on day 42 (m42) for the doripenem-recovery group. Statistically significant changes (Welch *t*-test; *p*-value < 0.05) are shown where red boxes mean a significant increase, yellow boxes mean a significant decrease, and white boxes indicate an insignificant change in the respective plasma metabolites compared to control group.

		Doripenem HD			Doripenem HD Recovery	Doripenem LD			Doripenem LD Recovery
Metabolite	Class	m7	m14	m28	m43	m7	m14	m28	m43
Tyrosine	Amino acids	0.71	0.95	0.8	0.86	0.83	1.07	0.82	0.93
Alanine	Amino acids	0.76	0.9	1	0.90	0.9	0.86	0.96	0.96
Glycine	Amino acids	0.87	0.94	0.81	1.00	0.89	0.82	0.77	0.97
Threonine	Amino acids	0.77	0.99	0.81	1.05	0.84	0.83	0.78	0.89
Tyrosine	Amino acids	0.75	0.77	0.76	0.97	0.83	1	0.92	0.94
3-Hydroxyindole	Amino acids-related	0.12	0.08	0.1	1.40	0.2	0.59	0.58	1.18
3-Hydroxyisobutyrate	Amino acids-related	0.99	0.83	0.72	0.95	0.86	0.92	0.76	1.12
Ketoleucine	Amino acids-related	0.67	0.68	0.86	0.80	0.84	0.82	0.9	0.74
3-Indoxylsulfate	Amino acids-related	0.2	0.05	0.18	1.08	0.18	0.73	0.48	0.88
Deoxyribonucleic acids, total	Carbohydrates and related	0.85	0.8	0.8	1.01	1.09	0.88	0.9	1.06
Glucose	Carbohydrates and related	0.68	0.59	0.68	0.68	0.91	0.72	0.81	1.06
Xylitol	Carbohydrates and related	0.69	0.4	0.69	0.60	0.93	0.62	0.52	1.03
Taurocholic acid	Complex lipids, fatty acids and related	4.45	6.15	4.22	0.51	1.67	1.41	0.95	1.41
Docosapentaenoic acid (C22:cis [7,10,13,16,19]5)	Complex lipids, fatty acids and related	0.86	0.65	0.58	1.13	0.8	0.67	0.64	0.49
Lysophosphatidylcholine (C20:4)	Complex lipids, fatty acids and related	0.94	0.77	0.84	1.13	0.95	0.9	0.85	0.84
Pyruvate	Energy metabolism and related	1.5	2.23	1.25	1.17	2.03	2.18	1.27	1.53
Pyruvate	Energy metabolism and related	1.52	2.26	1.06	1.43	2.49	2.87	1.32	1.62
Lactate	Energy metabolism and related	0.59	0.8	0.74	0.97	0.83	0.99	0.91	1.01
3-Phosphoglycerate (3-PGA)	Energy metabolism and related	0.43	0.27	0.72	0.83	0.56	0.35	0.92	0.94
Pregnenolone	Hormones, signal substances and related	0.52	0.49	0.38	0.77	1.04	0.89	1.04	0.78
Hippuric acid	Miscellaneous	0.31	0.31	0.46	1.67	0.38	0.42	0.32	0.99
Uric acid	Nucleobases and related	0.8	0.81	0.91	0.83	0.8	0.75	0.94	0.94
Cytosine	Nucleobases and related	0.78	0.81	0.85	0.97	1.03	0.93	0.95	1.06
Uric acid	Nucleobases and related	0.71	0.59	0.76	0.74	0.72	0.72	0.89	0.88
Threonic acid	Vitamins, cofactors and related	0.5	0.58	0.71	0.84	0.68	0.68	0.84	1.11
Phosphatidylcholine (C18:0,C20:3)		0.85	0.72	0.64	1.28	0.78	0.64	0.7	0.79

**Table 13:** Doripenem-induced plasma BA fold changes in male (m) Wistar rats (N = 5 per group) dosed with 100 (LD) and 1000 (HD) mg/kg bw/day observed on days 7, 14, and 28 (m7, m14, and m28) and on day 42 (m42) for the doripenem-recovery group. Statistically significant changes (Welch *t*-test; *p*-value < 0.05) are shown where red boxes mean a significant increase, yellow boxes mean a significant decrease, and white boxes indicate an insignificant change in the respective plasma metabolites compared to the control group in the respective plasma BAs.

		Doripenem HD			Doripenem HD Recovery	Doripenem LD			Doripenem LD Recovery
Metabolite	Class	m7	m14	m28	m43	m7	m14	m28	m43
Ursodeoxycholic acid	2° BA	0.09	0.38	0.18	0.55	1.11	5.52	2.15	0.13
Deoxycholic acid	2° BA	0	0	0.01	1.28	0.01	0.02	0.01	0.32
Cholic acid	1° BA	0.03	0.06	0.03	1.13	0.77	1.5	0.6	0.09
Chenodeoxycholic acid	1° BA	0.05	0.05	0.05	0.91	0.55	1.12	0.56	3.64
Taurocholic acid sodium salt	Taurine-conjugated 1° BA	3.6	3.71	4.27	0.68	2.27	0.86	0.83	1.41
Glycolithocholic Acid	Glycine-conjugated 2° BA	0.08	0.24	0.23	0.69	0.2	0.24	0.12	0.21
Taurolithocholic Acid	Taurine-conjugated 2° BA	0.06	0.1	0.21	1.16	0.06	0.06	0.07	0.69
Tauro-b-muricholic Acid	Taurine-conjugated 1° BA	4.21	4.48	4.86	0.90	2.46	1.4	1.19	1.39
Tauroursodeoxycholic Acid	Taurine-conjugated 2° BA	5.01	2.21	4.79	0.85	1.71	1.38	1.66	0.99
a-Muricholic Acid	1° BA	0.05	0.07	0.04	2.08	0.52	2.79	0.95	0.29
Taurochenodeoxycholate	Taurine-conjugated 1° BA	3.13	3.6	3.24	0.63	2.47	1.1	1.26	0.94
Taurodeoxycholate	Taurine-conjugated 2° BA	0.02	0.02	0.03	1.39	0.02	0.02	0.02	1.15
Glycocholic acid	Glycine-conjugated 1° BA	0.37	0.16	0.31	1.63	0.65	0.74	0.72	0.28
Glycochenodeoxycholic acid	Glycine-conjugated 1° BA	0.32	0.12	0.2	0.78	0.62	0.76	0.55	0.41
Glycodeoxycholate	Glycine-conjugated 2° BA	0	0	NA	0.45	0	0	0	0.30
Hyodeoxycholic acid	2° BA	0.04	0	0	0.48	0.01	0.01	0	0.72
o-Muricholic Acid	2° BA	0.14	0.02	0.01	0.81	0.11	0.28	0.08	0.54

**Table 14:** Percentages of the total number of significantly changed plasma (in light blue) and fecal (in light orange) metabolites at a *p*-value < 0.05 for the (**A**) HD and (**B**) LD groups of doripenem treatment plus the doripenem-recovery group and (**C**) HD and (**D**) LD groups of meropenem treatment in both sexes, respectively.

**(A)**	**Doripenem HD, males**									**Doripenem HD, females**								
	**Plasma**			**Plasma Recovery**	**Feces**			**Feces Recovery**		**Plasma**			**Plasma Recovery**	**Feces**			**Feces Recovery**	
	**7d**	**14d**	**28d**	**42d**	**7d**	**14d**	**28d**	**35d**	**42d**	**7d**	**14d**	**28d**	**42d**	**7d**	**14d**	**28d**	**35d**	**42d**
**% of total sig. changed metabolites**	20.27	16.89	19.59	7.43	47.75	48.55	49.04	18	14.95	19.59	12.16	18.92	18.58	58.54	54.5	54.5	24.76	22.5
**(B)**	**Doripenem LD, males**									**Doripenem LD, females**								
	**Plasma**			**Plasma Recovery**	**Feces**			**Feces Recovery**		**Plasma**			**Plasma Recovery**	**Feces**			**Feces Recovery**	
	**7d**	**14d**	**28d**	**42d**	**7d**	**14d**	**28d**	**35d**	**42d**	**7d**	**14d**	**28d**	**42d**	**7d**	**14d**	**28d**	**35d**	**42d**
**% of total sig. changed metabolites**	14.86	14.19	15.2	18.92	42.76	46.78	40.35	12.06	6.27	16.89	14.86	10.81	7.78	54.66	49.03	36.65	18	12.54
**(C)**					**Meropenem HD, males**							**Meropenem HD, females**						
					**Plasma**				**Feces**			**Plasma**			**Feces**			
					**7d**		**14d**	**28d**	**7d**	**14d**	**28d**	**7d**	**14d**	**28d**	**7d**	**14d**	**28d**	
**% of total sig. changed metabolites**					10.54		11.9	14.97	44.69	50.48	47.27	15.31	10.2	13.6	56.91	43.25	42.93	
**(D)**					**Meropenem LD, males**							**Meropenem LD, females**						
					**Plasma**				**Feces**			**Plasma**			**Feces**			
					**7d**		**14d**	**28d**	**7d**	**14d**	**28d**	**7d**	**14d**	**28d**	**7d**	**14d**	**28d**	
**% of total sig. changed metabolites**					6.8		7.82	12.24	46.46	34.73	32.96	9.86	12.24	8.5	49.36	45.18	31.51	

## Data Availability

All data are stored under GLP or GLP-like archives at BASF SE, Ludwigshafen am Rhein, Germany. Metabolome data are stored in MetaMapTox database, BASF.

## Supplementary Materials

The following supporting information can be downloaded at: https://www.mdpi.com/article/10.3390/microorganisms11020533/s1, Figure S1: Principal Coordinate Analysis (PCoA) of bacterial families from controls, meropenem, and doripenem antibiotic-treated rats; Figure S2: Differential abundances of dominant bacterial families in carbapenem-treated samples; Table S1: Meropenem-induced feces metabolite fold changes in female Wistar rats; Table S2: Meropenem-induced feces metabolite fold changes in male Wistar rats; Table S3: Doripenem-induced feces metabolite fold changes in female Wistar rats; Table S4: Doripenem-induced feces metabolite fold changes in male Wistar rats; Table S5: Summarized table indicating the trend of significant alterations in plasma biomarkers.
