# Supplementary material for: Gut Microbiota as Well as Metabolomes of Wistar Rats Recover within Two Weeks after Doripenem Antibiotic Treatment
Source: Microorganisms. 2023 Feb 20;11(2):533. doi: 10.3390/microorganisms11020533 (PMC9959319; doi:10.3390/microorganisms11020533)
Supplement: Supplementary file 1 [file microorganisms-11-00533-s001.zip › microorganisms-2203264-supplementary.pdf]

Supplementary figures

(A) (B)

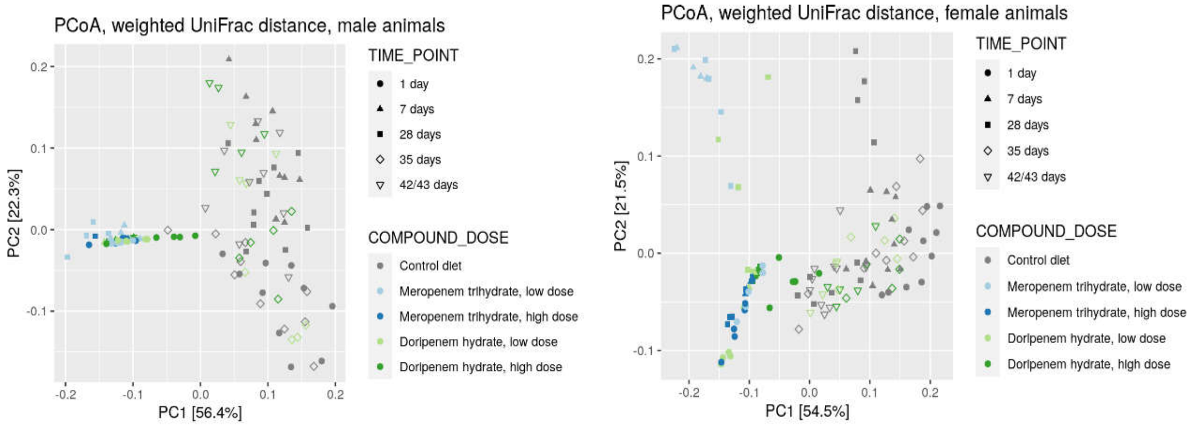

Figure S1: Principal Coordinate Analysis (PCoA) of bacterial families from controls, meropenem and doripenem antibiotic-treated rats at different time points using a phylogenetic-based distance matrix, weighted UniFrac distance, of samples from A) males and B) females are presented. Consistent to Bray-curtis based analysis, samples from day 1 clustered separately from the rest time points and the samples from animals treated with antibiotic from days 7 and 28 all cluster together and are separated both from controls and day 1 treated animals in both the sexes. Finally, samples from control animals at days 35 and 42/43 clustered together with the doripenem-recovery groups at one and two weeks after cessation of doripenem treatment indicating recovery reactions.

(A)

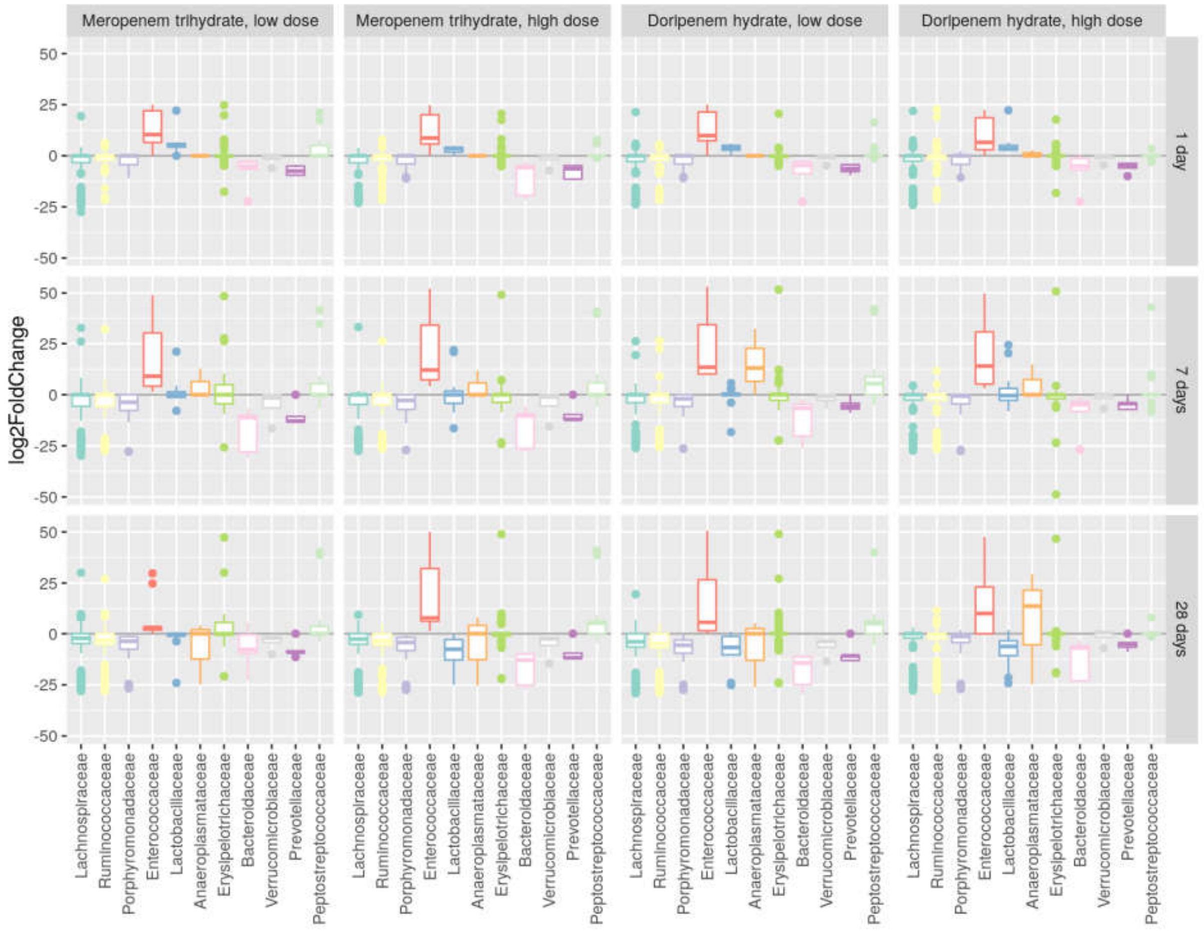

(B)

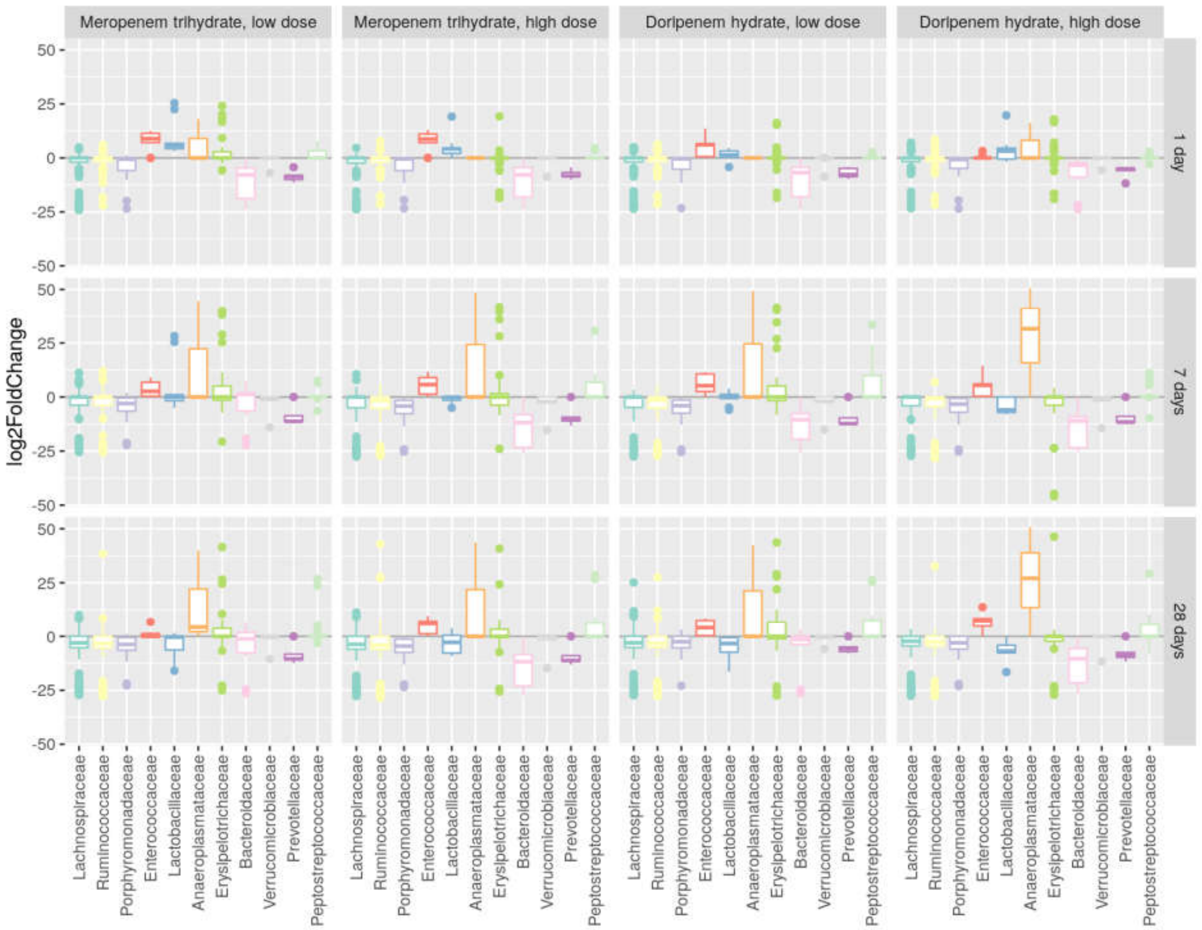

Figure S2: Differential abundances in dominant bacterial families in carabapenem treated samples from low (LD) and high (HD) dose groups relative to controls in males (A) and females (B). Differential abundance analysis was performed using log2FC (log2 fold change) values.

Supplementary tables

Table S1: Meropenem-induced feces metabolite fold changes in female (f) Wistar rats (N=5 per group) dosed with 100 (LD) and 300 (HD) mg/kg bw/day for 7, 14 and 28 days (f7, f14 and f28). Statistically significant changes (Welch-t-test; p-value < 0.05) are shown in bold numbers where red boxes mean a significant increase in the respective fecal metabolites and yellow a significant reduction compared to control levels.

		Meropenem trihydrate HD			Meropenem trihydrate LD		
**Metabolite**	**Class**	**f7**	**f14**	**f28**	**f7**	**f14**	**f28**
**1,2-Anhydroribose**	Carbohydrates and related	**4.24**	**4.94**	**5.18**	**3.20**	**2.33**	1.62
**1,4-Hydroquinone**	Miscellaneous	**2.71**	**2.98**	**1.82**	**1.53**	1.27	1.09
**14-Methylhexadecanoic acid**	Complex lipids, fatty acids and related	0.27	0.36	0.25	0.73	0.74	1.15
**15-Methylhexadecanoic acid**	Complex lipids, fatty acids and related	0.27	0.38	0.40	0.68	0.81	1.69
**2-Aminoadipic acid**	Amino acids related	**3.92**	**7.00**	**3.18**	**2.01**	**3.05**	1.32
**2-Hydroxybehenic acid (C22:0)**	Complex lipids, fatty acids and related	0.30	0.83	0.55	0.64	0.73	0.77
**2-Hydroxycerotic acid (C26:0)**	Complex lipids, fatty acids and related	0.38	0.70	0.48	0.52	0.68	0.60

**2-Hydroxynervonic acid (C24:1)**	Complex lipids, fatty acids and related	0.24	0.85	0.53	0.49	0.95	0.59
**2-Hydroxypentacosanoic acid (C25:0)**	Complex lipids, fatty acids and related	0.31	0.76	0.50	0.59	0.80	0.69
**3-Hydroxybutyrate**	Energy metabolism and related	**10.82**	**17.59**	**4.55**	**4.38**	**3.45**	2.54
**3-Hydroxyphenylacetic acid**	Amino acids related	0.09	0.05	0.07	0.09	0.04	0.15
**4-Hydroxyphenylacetic acid**	Amino acids related	**20.99**	**11.73**	**10.60**	**28.18**	1.83	0.43
**4-Hydroxysphinganine (t18:0, Phytosphingosine), total**	Complex lipids, fatty acids and related	**4.10**	**9.81**	**2.93**	**8.68**	**7.90**	**1.83**
**5-O-Methylsphingosine (d18:1)**	Complex lipids, fatty acids and related	1.71	**2.65**	**2.26**	**3.15**	**2.83**	**2.53**
**5-Oxoproline**	Amino acids related	**13.68**	**22.69**	**5.44**	**6.91**	**16.24**	**4.98**
**6-Hydroxynicotinic acid**	Vitamins, cofactors and related	**8.88**	**7.56**	**10.79**	**7.52**	**14.80**	**19.69**
**Adenine**	Nucleobases and related	0.33	0.32	0.37	0.29	0.28	0.50
**Allantoin**	Nucleobases and related	**359.95**	**207.31**	**152.90**	**118.28**	**16.54**	**12.60**
**alpha-Amyrin TMS**	Unknown	0.43	1.13	0.43	0.46	0.60	0.65
**alpha-Tocopherol**	Vitamins, cofactors and related	0.57	0.66	0.80	0.97	0.92	1.18
**alpha-Tocopherol**	Unknown	0.07	0.21	0.07	0.04	0.07	0.13
**alpha-Tocotrienol**	Unknown	0.09	0.37	0.14	0.06	0.07	0.18
**Arabinose**	Carbohydrates and related	**3.46**	**2.48**	1.62	2.23	1.92	**5.30**
**Arginine**	Amino acids	1.68	**3.24**	**2.71**	**2.85**	**2.79**	3.22
**Azelaic acid (Dicarboxylic acid; C9:0)**	Complex lipids, fatty acids and related	**3.94**	1.61	**2.04**	**3.43**	**3.60**	**1.75**
**beta-/gamma-Tocotrienol**	Unknown	0.63	0.63	0.95	0.59	0.55	1.11
**beta-Alanine**	Amino acids related	**9.72**	**5.63**	**8.30**	**25.54**	18.45	0.71
**beta-Amyrin TMS**	Unknown	0.49	1.07	0.56	0.55	0.70	0.82
**beta-Muricholic acid**	Complex lipids, fatty acids and related	**7.76**	3.95	**3.66**	**16.51**	**4.56**	2.59
**beta-Sitosterol, total**	Miscellaneous	**2.93**	**3.01**	**3.31**	**3.80**	**3.28**	**2.67**
**Bile acid No 05**	Complex lipids, fatty acids and related	**3.08**	**2.04**	**2.34**	**8.74**	**17.53**	**5.40**
**Bile acid No 07**	Complex lipids, fatty acids and related	0.40	0.27	0.50	0.66	0.46	0.60
**Biliverdin**	Unknown	0.43	0.09	0.11	0.93	0.17	0.15
**bis-Glyceryl phosphate, polar fraction**	Miscellaneous	**3.12**	**1.50**	1.30	1.03	1.16	0.66
**Campesterol, total**	Miscellaneous	**3.00**	**2.80**	**2.94**	**3.63**	**3.60**	**2.35**
**Cerebronic acid (2-OH-C24:0)**	Complex lipids, fatty acids and related	0.32	0.92	0.57	0.57	0.99	0.61
**Cholesterol, total**	Complex lipids, fatty acids and related	**3.12**	**2.15**	**2.29**	**4.83**	**2.59**	1.34
**Cholic acid**	Unknown	**10.78**	9.57	**7.33**	**17.48**	1.14	0.91
**Citrulline**	Unknown	0.31	0.48	0.37	0.76	0.59	0.97
**Coenzyme Q10**	Unknown	0.55	0.63	1.05	0.77	0.82	0.84
**Cortisol (lipid)**	Unknown	0.75	0.61	0.67	0.62	0.69	0.56
**Creatinine**	Amino acids related	**113.44**	**146.37**	**115.35**	**111.11**	**152.38**	**175.87**

**Creatinine**	Unknown	**137.36**	**138.42**	**48.09**	**88.40**	**226.47**	**71.94**
**Cysteine**	Amino acids	**7.36**	**7.68**	**7.82**	**6.07**	**5.84**	**3.41**
**Cytosine**	Nucleobases and related	**10.59**	**5.41**	**2.67**	**4.13**	**1.64**	1.29
**Dehydroalanine**	Amino acids related	**8.09**	**8.74**	**5.81**	**7.21**	**5.87**	**3.55**
**Deoxycholic acid**	Complex lipids, fatty acids and related	0.16	0.06	0.24	0.37	0.26	0.59
**Deoxycholic acid**	Unknown	0.01	0.01	0.06	0.09	0.16	0.17
**Eicoasenoic acid (C20:cis[11]1)**	Complex lipids, fatty acids and related	0.77	0.54	1.50	0.87	0.69	1.46
**Eicosadienoic acid (C20:2) No 01**	Complex lipids, fatty acids and related	0.30	0.38	0.41	0.45	0.39	0.68
**Eicosadienoic acid (C20:2) No 02**	Complex lipids, fatty acids and related	0.33	0.40	0.37	0.40	0.36	0.38
**epsilon-Acetyllysine**	Unknown	**4.29**	**5.54**	**5.55**	**4.42**	**4.08**	**5.99**
**erythro-Dihydrosphingosine (d18:0)**	Complex lipids, fatty acids and related	0.18	0.40	0.28	1.46	1.45	**2.68**
**Fucose**	Carbohydrates and related	**4.89**	3.79	**2.57**	4.06	4.90	3.88
**Fucosterol, total**	Miscellaneous	**2.74**	**2.16**	**2.70**	**2.46**	**2.32**	**2.41**
**Fumarate**	Energy metabolism and related	**3.79**	**6.63**	**3.60**	**2.40**	**3.74**	2.52
**Galactose, lipid fraction**	Complex lipids, fatty acids and related	**1.48**	**1.95**	**2.46**	**2.55**	1.39	1.55
**gamma-Aminobutyrate (GABA)**	Hormones, signal substances and related	**5.06**	2.03	**1.71**	2.70	2.96	0.78
**gamma-Tocopherol**	Vitamins, cofactors and related	0.60	0.58	0.79	0.64	0.75	0.70
**Glucose, lipid fraction**	Complex lipids, fatty acids and related	**1.85**	**2.25**	**2.10**	**3.37**	1.80	1.12
**Glucose-1-phosphate**	Carbohydrates and related	**4.38**	**7.27**	**2.88**	**4.32**	**3.95**	**3.13**
**Glucuronic acid**	Carbohydrates and related	**3.76**	**4.24**	**3.88**	**3.34**	1.70	1.63
**Glutamate**	Amino acids	**9.24**	**15.75**	**5.45**	**8.64**	**12.63**	4.78
**Glutamate**	Unknown	**1.80**	**3.30**	1.78	**1.92**	**2.64**	2.05
**Glutamic acid 2TMS ME (lipid)**	Unknown	**2.54**	**2.52**	**1.98**	**2.78**	**2.48**	1.97
**Glutamine**	Amino acids	**5.97**	**5.45**	**7.15**	**4.06**	**4.56**	**3.52**
**Glutarate**	Amino acids related	0.45	0.47	0.64	1.44	0.31	0.66
**Glycerol-3-phosphate, polar fraction**	Complex lipids, fatty acids and related	**10.34**	**4.53**	**5.65**	**7.96**	**3.79**	1.57
**Glycine, lipid fraction**	Miscellaneous	0.66	0.67	0.66	**1.58**	**1.50**	**2.18**
**Glycine, lipid fraction**	Miscellaneous	0.50	0.54	0.60	**1.67**	**1.51**	**2.49**
**Glycolate**	Miscellaneous	**4.29**	**4.11**	**2.91**	1.67	**2.22**	1.18
**Heneicosanoic acid (C21:0)**	Complex lipids, fatty acids and related	0.40	0.55	0.47	0.59	0.50	0.72
**Heptadecanoic acid (C17:0)**	Complex lipids, fatty acids and related	0.26	0.27	0.34	0.38	0.37	0.83
**Hexadecanol**	Complex lipids, fatty acids and related	0.05	0.21	0.09	0.14	0.08	0.15
**Hyocholic acid**	Complex lipids, fatty acids and related	0.81	0.36	0.28	0.83	0.33	0.64
**Hyodeoxycholic acid**	Unknown	0.01	0.01	0.01	0.02	0.01	0.04
**Hyodeoxycholic acid**	Complex lipids, fatty acids and related	0.10	0.05	0.47	0.14	0.12	0.59

**Indole-3-acetic acid**	Amino acids related	**11.09**	3.86	**10.03**	**5.12**	**3.07**	1.38
**isoLCA**	Unknown	0.01	0.01	0.01	0.08	0.28	0.31
**Isopalmitic acid (C16:0)**	Complex lipids, fatty acids and related	0.27	0.34	0.18	0.81	0.62	1.11
**Kynurenic acid**	Unknown	**4.23**	**4.25**	**5.42**	**5.83**	**7.40**	**7.23**
**Linoleic acid (C18:cis[9,12]2)**	Complex lipids, fatty acids and related	**4.66**	**4.63**	**7.29**	**6.33**	**5.57**	**6.28**
**Linolenic acid (C18:cis[9,12,15]3)**	Complex lipids, fatty acids and related	**4.74**	**4.66**	**7.03**	**5.40**	**4.67**	**6.46**
**Lithocholic acid**	Unknown	0.02	0.03	0.02	0.08	0.15	0.15
**Mannose**	Carbohydrates and related	**11.01**	**12.32**	**4.63**	**12.07**	7.01	0.46
**Methionine**	Unknown	0.19	0.23	0.21	0.77	0.76	1.08
**Myristic acid (C14:0)**	Complex lipids, fatty acids and related	0.31	0.20	0.53	0.61	0.31	1.03
**N2-Acetyllysine**	Amino acids related	**4.82**	**5.67**	**4.28**	**6.23**	**3.79**	3.62
**N-Acetyl-aspartic acid**	Unknown	**5.94**	**4.76**	**3.34**	**1.98**	2.37	2.01
**N-Acetylglucosamine**	Unknown	0.12	0.13	0.19	0.20	0.36	0.28
**N-Acetylserine**	Unknown	**4.64**	**11.16**	5.19	4.32	0.99	0.32
**N-Methylglutamate**	Unknown	0.03	0.05	0.02	0.06	0.03	0.07
**Norleucine**	Miscellaneous	0.42	0.42	0.36	0.34	0.36	0.46
**Octadecanol**	Complex lipids, fatty acids and related	0.08	0.37	0.19	0.11	0.09	0.38
**Oleic acid (C18:cis[9]1)**	Complex lipids, fatty acids and related	**2.26**	2.08	**5.19**	**2.82**	**2.17**	**4.55**
**omega-Muricholic acid**	Complex lipids, fatty acids and related	0.51	0.31	0.23	0.88	0.51	0.39
**o-Muricholic Acid**	Unknown	0.19	0.04	0.03	0.60	0.12	0.13
**Palmitic acid (C16:0)**	Complex lipids, fatty acids and related	0.71	0.48	1.27	1.16	0.61	1.38
**Pentadecanol**	Complex lipids, fatty acids and related	0.42	0.45	0.56	0.54	0.50	0.90
**Phosphoenolpyruvate (PEP)**	Energy metabolism and related	**6.07**	**6.48**	**4.24**	**3.52**	**3.75**	**3.47**
**Pipecolic acid**	Unknown	0.40	0.23	0.44	0.74	0.47	1.00
**plausible 18:2-Lyso PC (Na-Addukt)**	Unknown	**3.02**	1.15	**2.62**	**2.48**	**2.98**	1.84
**plausible 2-Hydroxyarachidic acid (2-OH-C20:0) TMS ME**	Unknown	0.25	0.69	0.50	0.60	0.70	0.65
**plausible 2-Hydroxytricosanoic acid (2-OH-C23:0) TMS ME**	Unknown	0.29	0.92	0.59	0.66	0.69	0.77
**plausible Heptadecanol TMS**	Unknown	0.01	0.10	0.02	0.01	0.11	0.43
**plausible SM (d18:1, C16:0) Na-Addukt**	Unknown	**5.91**	**4.22**	**5.33**	**4.99**	**3.63**	1.54
**proposed Sphingomyelin (d18:2,C16:0)**	Unknown	**4.28**	**3.34**	**4.27**	**3.43**	**2.51**	0.98
**putative 12-Methylmyristic acid TMS**	Unknown	0.02	0.11	0.04	0.91	0.91	1.89
**putative Anhydroglucuronate 4TMS**	Unknown	**3.10**	**4.24**	**2.95**	**3.47**	**4.12**	**2.82**
**putative beta-Muricholic acid 3TMS ME (Byprodukt)**	Unknown	**6.13**	**2.99**	2.10	**13.29**	**4.16**	**2.55**
**putative Bis-(glycerol)-phosphate**	Unknown	0.09	0.09	0.13	**3.90**	**3.41**	**4.51**
**putative Choline plasmalogen (C36:2)**	Unknown	**6.22**	1.49	**2.68**	**2.97**	1.25	1.59
**putative Deoxycholylglycine deriv #1**	Unknown	0.02	0.03	0.04	0.10	0.21	0.30

**putative Hydroxy-eicosan OTMS**	Unknown	0.17	0.29	0.40	0.17	0.17	0.33
**putative Hyodeoxycholic acid 3TMS**	Unknown	0.03	0.06	0.08	0.03	0.04	0.11
**putative Lithocholic acid OTMS ME**	Unknown	0.01	0.02	0.03	0.05	0.18	0.19
**putative MeOx impurity (Blank)**	Unknown	4.81	6.24	2.63	3.25	3.08	1.43
**Putrescine**	Miscellaneous	71.60	61.04	5.54	82.41	18.52	0.94
**Pyruvate**	Energy metabolism and related	2.81	5.66	3.19	2.37	2.91	1.65
**ribo-4-Hydroxy-8E-sphingenine**	Complex lipids, fatty acids and related	0.80	3.04	1.64	1.42	2.16	1.77
**Sarcosine**	Amino acids related	0.67	0.80	0.71	0.63	0.53	0.67
**scyllo-Inositol**	Carbohydrates and related	34.05	22.75	7.73	5.29	7.42	1.49
**Serine, lipid fraction**	Miscellaneous	0.62	0.51	0.71	1.31	1.32	2.07
**Serine, lipid fraction**	Miscellaneous	0.69	0.48	0.63	1.33	1.37	2.39
**Shikimic acid**	Miscellaneous	1593.76	1731.27	3.31	1494.67	512.47	1.67
**Stearic acid (C18:0)**	Complex lipids, fatty acids and related	0.68	0.50	0.66	0.83	0.56	0.60
**Stigmasterol, total**	Miscellaneous	2.22	1.85	2.36	2.32	2.26	2.41
**Taurine**	Unknown	15.00	9.83	12.50	27.07	15.95	15.19
**Tauro-b-muricholic Acid**	Unknown	3.51	2.01	3.42	2.14	1.30	1.10
**Taurochenodeoxycholate**	Unknown	2.08	1.43	2.60	1.71	1.56	1.17
**Taurocholic Acid 3-sulfate**	Unknown	185.39	197.22	280.09	40.98	81.49	6.10
**Taurocholic acid sodium salt**	Unknown	4.70	2.04	3.85	3.41	1.87	1.14
**Taurolithocholic Acid**	Unknown	0.04	0.12	0.15	0.41	0.61	0.78
**Tetradecanol**	Complex lipids, fatty acids and related	0.12	0.13	0.07	0.21	0.08	0.10
**Thiamine (Vitamin B1)**	Unknown	0.27	0.13	0.18	0.74	0.54	0.59
**threo-Sphinganine 3TMS**	Unknown	0.13	0.44	0.37	4.20	3.95	6.54
**threo-Sphingosine (d18:1)**	Complex lipids, fatty acids and related	1.86	1.81	1.95	3.26	2.31	2.06
**trans-4-Hydroxyproline**	Amino acids related	3.59	4.56	3.70	3.59	4.59	3.28
**trans-Ferulic acid**	Miscellaneous	8.57	5.33	3.48	5.96	4.77	3.18
**trans-Sinapic acid**	Miscellaneous	41.49	34.45	22.76	10.49	8.45	5.06
**Tryptophan**	Amino acids	16.56	34.35	4.70	3.75	4.53	2.82
**Tyramine**	Amino acids related	26.44	39.24	12.58	6.25	4.51	6.52
**Tyrosine**	Unknown	0.22	0.24	0.32	0.65	0.87	1.05
**Ubichinone-30 (Coenzyme Q6)**	Unknown	0.64	0.66	0.98	0.95	0.91	0.81
**Ubichinone-35 (Coenzyme Q7)**	Unknown	0.63	0.74	0.93	0.89	0.98	0.83
**Ubichinone-45 (Coenzyme Q9)**	Unknown	0.63	0.70	0.94	0.91	0.97	0.84
**Unknown lipid MB (828450473)**	Unknown	4.29	4.01	3.13	3.89	3.12	2.23
**Unknown lipid MB (828451241)**	Unknown	0.10	0.13	0.19	0.30	0.06	0.60
**Unknown lipid MB (828451275)**	Unknown	0.79	0.32	0.30	0.46	0.36	0.26
**Unknown lipid MB (828451312)**	Unknown	0.53	0.31	0.57	1.72	0.37	1.04
**Unknown lipid MB (828451316)**	Unknown	3.45	1.91	3.20	4.35	3.08	1.70
**Unknown lipid MB (828451443)**	Unknown	0.67	0.58	1.01	0.98	0.81	1.09
**Unknown lipid MB (828451445)**	Unknown	0.24	0.23	0.36	0.38	0.33	0.86

**Unknown lipid MB (828451931)**	Unknown	0.00	0.07	0.04	0.01	0.10	0.28
**Unknown lipid MB (828451933)**	Unknown	0.08	0.10	0.12	0.29	0.09	0.52
**Unknown lipid MB (828451940)**	Unknown	0.00	0.34	0.05	0.01	0.25	0.24
**Unknown lipid MB (828452038)**	Unknown	0.01	0.01	0.01	**1.98**	**2.60**	**3.42**
**Unknown lipid MB (828452040)**	Unknown	0.12	0.09	0.32	1.69	1.59	**2.46**
**Unknown lipid MB (828452041)**	Unknown	0.02	0.43	0.11	0.06	0.36	0.30
**Unknown lipid MB (828452042)**	Unknown	0.01	0.02	0.00	0.01	0.01	0.01
**Unknown lipid MB (828452043)**	Unknown	0.02	0.47	0.04	0.02	0.41	0.22
**Unknown lipid MB (828452047)**	Unknown	0.01	0.75	0.09	0.02	0.46	0.34
**Unknown lipid MB (828452058)**	Unknown	0.11	0.20	0.06	0.19	0.42	0.22
**Unknown lipid MB (828452059)**	Unknown	0.34	0.18	0.09	0.62	0.20	0.40
**Unknown lipid MB (828452060)**	Unknown	0.02	0.00	0.02	0.01	0.01	0.11
**Unknown lipid MB ESI neg (858450047)**	Unknown	**3.31**	**1.37**	**2.96**	1.80	**2.05**	2.15
**Unknown lipid MB ESI neg (858450065)**	Unknown	0.06	0.02	0.06	0.08	0.09	0.21
**Unknown lipid MB ESI neg (858450068)**	Unknown	0.46	0.80	0.50	0.47	0.66	0.49
**Unknown lipid MB ESI neg (858450071)**	Unknown	**5.12**	2.20	**3.53**	**4.51**	**3.90**	**3.09**
**Unknown lipid MB ESI pos (848450056)**	Unknown	**5.71**	**2.54**	**3.79**	**3.44**	**2.89**	**3.34**
**Unknown lipid MB ESI pos (848450067)**	Unknown	**2.03**	1.93	**2.38**	**2.17**	1.69	**2.15**
**Unknown lipid MB ESI pos (848450068)**	Unknown	**3.70**	1.43	**2.14**	**3.69**	**2.46**	1.78
**Unknown lipid MB ESI pos (848450069)**	Unknown	**6.06**	1.12	**2.25**	**4.61**	1.25	1.59
**Unknown lipid MB ESI pos (848450083)**	Unknown	**4.73**	**2.65**	**1.80**	**3.59**	**2.41**	0.82
**Unknown lipid MB ESI pos (848450084)**	Unknown	**3.86**	**2.42**	1.46	**2.79**	**2.69**	0.59
**Unknown lipid MB ESI pos (848450092)**	Unknown	0.40	0.17	0.28	0.55	1.14	1.27
**Unknown lipid MB ESI pos (848450093)**	Unknown	0.59	0.46	0.55	0.58	1.38	2.01
**Unknown lipid MB ESI pos (848450094)**	Unknown	**4.11**	**6.98**	1.61	1.98	1.51	1.52
**Unknown lipid MB ESI pos (848450095)**	Unknown	0.58	0.33	0.69	**5.63**	**3.74**	1.42
**Unknown lipid MB ESI pos (848450096)**	Unknown	**3.28**	1.25	**2.19**	**3.50**	**11.63**	**4.24**
**Unknown lipid MB ESI pos (848450097)**	Unknown	**7.77**	**1.87**	**4.11**	**3.27**	**2.61**	**1.58**
**Unknown lipid MB ESI pos (848450098)**	Unknown	**2.55**	**1.36**	1.39	1.58	1.43	1.10
**Unknown lipid MB ESI pos (848450100)**	Unknown	**3.36**	1.06	**2.00**	**4.77**	**2.56**	1.48
**Unknown lipid MB ESI pos (848450102)**	Unknown	**4.87**	1.46	**2.40**	**2.31**	1.74	1.63
**Unknown lipid MB ESI pos (848450103)**	Unknown	**4.91**	**1.90**	**3.72**	**2.68**	**1.61**	1.24
**Unknown lipid MB ESI pos (848450105)**	Unknown	**9.05**	**2.88**	**9.13**	**4.19**	**4.61**	1.57
**Unknown lipid MB ESI pos (848450106)**	Unknown	**8.23**	**2.10**	**3.00**	**5.64**	**2.42**	0.81

**Unknown lipid MB ESI pos (848450107)**	Unknown	**2.97**	1.15	**2.19**	**2.62**	**1.79**	**2.02**
**Unknown lipid MB ESI pos (848450108)**	Unknown	**5.37**	**1.55**	**3.81**	**4.27**	**2.62**	**3.26**
**Unknown lipid MB ESI pos (848450109)**	Unknown	**5.39**	1.56	**3.09**	**3.97**	1.80	1.53
**Unknown lipid MB ESI pos (848450110)**	Unknown	**9.35**	**4.25**	**3.91**	**7.78**	**2.35**	0.97
**Unknown lipid MB ESI pos (848450111)**	Unknown	**3.86**	**2.78**	**3.17**	**4.81**	**3.87**	**3.66**
**Unknown lipid MB ESI pos (848450112)**	Unknown	**3.97**	1.17	**2.89**	**3.92**	1.54	**2.70**
**Unknown lipid MB ESI pos (848450113)**	Unknown	**7.26**	**3.23**	**6.76**	**6.65**	**4.53**	**6.23**
**Unknown lipid MB ESI pos (848450114)**	Unknown	**7.35**	**1.68**	**3.87**	**4.93**	**2.45**	**2.20**
**Unknown lipid MB ESI pos (848450115)**	Unknown	**4.08**	1.26	**2.63**	**2.89**	1.01	0.98
**Unknown lipid MB ESI pos (848450116)**	Unknown	**5.77**	**2.74**	2.07	**4.00**	**2.50**	0.77
**Unknown lipid MB ESI pos (848450120)**	Unknown	**3.94**	1.19	**3.12**	**2.49**	1.37	1.27
**Unknown lipid MB ESI pos (848450123)**	Unknown	**3.05**	1.30	**2.51**	**3.35**	**3.15**	0.97
**Unknown lipid MB ESI pos (848450129)**	Unknown	**3.67**	**2.43**	2.87	**4.01**	**2.35**	0.90
**Unknown lipid MB ESI pos (848450130)**	Unknown	**3.12**	**1.80**	1.89	**3.33**	1.34	0.87
**Unknown lipid MB ESI pos (848450131)**	Unknown	**6.11**	**3.39**	**4.21**	**6.06**	**3.02**	1.17
**Unknown lipid MB ESI pos (848450132)**	Unknown	**5.87**	**3.53**	**3.49**	**5.40**	**3.43**	0.91
**Unknown lipid MB ESI pos (848450133)**	Unknown	**3.47**	**2.03**	1.57	**3.34**	1.74	0.63
**Unknown lipid MB ESI pos (848450135)**	Unknown	**3.71**	**1.73**	1.90	**3.37**	**1.97**	1.15
**Unknown lipid MB ESI pos (848450138)**	Unknown	**4.33**	**3.67**	**2.59**	**4.52**	**3.04**	0.79
**Unknown lipid MB ESI pos (848450144)**	Unknown	**3.91**	**3.68**	**1.96**	**3.71**	**3.00**	1.18
**Unknown lipid MB ESI pos (848450146)**	Unknown	**3.42**	**4.32**	**2.27**	**2.81**	**3.83**	1.18
**Unknown lipid MB ESI pos (848450147)**	Unknown	**1.42**	**2.50**	**1.79**	**1.96**	**1.94**	1.23
**Unknown lipid MB ESI pos (848450148)**	Unknown	**3.74**	**5.67**	**3.07**	**4.58**	**4.31**	1.31
**Unknown lipid MB ESI pos (848450152)**	Unknown	**3.08**	**2.74**	1.83	**3.03**	**2.29**	1.08
**Unknown lipid MB ESI pos (848450153)**	Unknown	**2.24**	**3.00**	1.31	**2.35**	1.69	1.12
**Unknown lipid MB ESI pos (848450156)**	Unknown	**1.41**	**2.78**	0.86	**2.37**	**1.81**	1.18
**Unknown lipid MB ESI pos (848450158)**	Unknown	**2.26**	**3.46**	1.37	**4.46**	**2.40**	1.44
**Unknown lipid MB ESI pos (848450161)**	Unknown	**1.58**	**4.77**	1.71	**2.75**	**3.00**	**2.71**
**Unknown lipid MB ESI pos (848450162)**	Unknown	**1.74**	**3.79**	1.54	2.49	**2.95**	1.66

**Unknown lipid MB ESI pos (848450163)**	Unknown	1.93	**4.61**	**2.15**	**3.16**	**3.66**	**2.83**
**Unknown lipid MB ESI pos (848450168)**	Unknown	**1.67**	**2.89**	**1.64**	**2.78**	**2.18**	**1.44**
**Unknown lipid MB ESI pos (848450169)**	Unknown	1.72	**7.09**	**2.14**	2.69	**3.86**	**1.79**
**Unknown lipid MB ESI pos (848450196)**	Unknown	**12.31**	**14.90**	**9.85**	**13.22**	**15.02**	**11.80**
**Unknown lipid MB ESI pos (848450201)**	Unknown	**9.34**	**11.52**	**8.56**	**10.29**	**15.98**	**10.08**
**Unknown lipid MB ESI pos (848450202)**	Unknown	**4.65**	**4.08**	**4.76**	**4.08**	**4.94**	**4.85**
**Unknown lipid MB ESI pos (848450204)**	Unknown	**12.21**	**16.35**	**9.62**	**14.19**	**20.96**	**12.03**
**Unknown lipid MB ESI pos (848450205)**	Unknown	**12.84**	**10.08**	**8.98**	**12.46**	**13.11**	**9.57**
**Unknown lipid MB ESI pos (848450206)**	Unknown	**14.64**	**25.69**	**13.66**	**15.27**	**32.02**	**16.14**
**Unknown lipid MB ESI pos (848450207)**	Unknown	**11.77**	**18.30**	**13.64**	**14.06**	**27.27**	**15.83**
**Unknown lipid MB ESI pos (848450208)**	Unknown	**11.89**	**16.88**	**10.61**	**13.06**	**21.14**	**11.86**
**Unknown lipid MB ESI pos (848450209)**	Unknown	**8.33**	**8.18**	**7.42**	**8.39**	**9.53**	**7.81**
**Unknown lipid MB ESI pos (848450210)**	Unknown	**11.08**	**9.66**	**8.96**	**11.52**	**14.92**	**11.02**
**Unknown lipid MB ESI pos (848450212)**	Unknown	**8.77**	**13.16**	**7.48**	**8.72**	**13.96**	**8.21**
**Unknown lipid MB ESI pos (848450213)**	Unknown	**11.39**	**13.38**	**9.43**	**12.40**	**14.68**	**10.31**
**Unknown lipid MB ESI pos (848450214)**	Unknown	**9.59**	**14.51**	**8.75**	**9.71**	**15.64**	**9.34**
**Unknown lipid MB ESI pos (848450215)**	Unknown	**12.14**	**15.84**	**9.73**	**10.82**	**23.19**	**11.28**
**Unknown lipid MB ESI pos (848450216)**	Unknown	**9.81**	**10.03**	**8.72**	**10.48**	**11.72**	**9.69**
**Unknown lipid MB ESI pos (848450218)**	Unknown	**12.47**	**13.75**	**10.74**	**11.05**	**16.26**	**11.52**
**Unknown lipid MB ESI pos (848450219)**	Unknown	**9.37**	**11.02**	**9.02**	**9.62**	**15.08**	**10.85**
**Unknown polar MB (838450146)**	Unknown	**4.27**	**5.06**	**2.68**	**1.89**	**1.47**	1.63
**Unknown polar MB (838450618)**	Unknown	**3.08**	**2.01**	**2.39**	**2.73**	**1.37**	1.18
**Unknown polar MB (838450621)**	Unknown	**12.13**	**10.75**	4.98	**9.43**	**3.59**	0.92
**Unknown polar MB (838450692)**	Unknown	0.12	0.25	0.12	0.18	0.13	0.16
**Unknown polar MB (838450761)**	Unknown	**2.55**	1.44	**2.39**	**3.93**	2.15	**1.36**
**Unknown polar MB (838450807)**	Unknown	**2.74**	1.76	**1.89**	**1.89**	**2.42**	**2.33**
**Unknown polar MB (838450816)**	Unknown	0.01	0.01	0.00	0.01	0.00	0.01
**Unknown polar MB (838451614)**	Unknown	0.01	0.02	0.02	0.02	0.01	0.03
**Unknown polar MB (838451982)**	Unknown	**8.38**	**3.67**	**5.01**	**6.72**	**3.44**	1.85
**Unknown polar MB (838452021)**	Unknown	0.42	0.39	0.63	0.58	0.40	0.60
**Unknown polar MB (838452022)**	Unknown	**2.48**	**3.56**	**2.14**	**2.12**	**1.85**	1.49
**Unknown polar MB (838452079)**	Unknown	**3.91**	**3.37**	**2.35**	**2.95**	**2.23**	**3.63**
**Unknown polar MB (838452178)**	Unknown	0.12	0.17	0.59	0.28	0.21	1.07
**Unknown polar MB (838452196)**	Unknown	**2.80**	**3.25**	**2.06**	**1.73**	**1.74**	**1.60**

**Unknown polar MB (838452482)**	Unknown	**313.21**	**115.05**	**253.60**	**156.16**	**16.64**	**15.63**
**Unknown polar MB (838452999)**	Unknown	**53.93**	**56.90**	**37.37**	**8.32**	**20.15**	**4.97**
**Unknown polar MB (838453000)**	Unknown	**7.71**	**3.49**	**4.13**	**4.07**	**2.52**	2.01
**Unknown polar MB (838453005)**	Unknown	0.18	0.22	0.27	0.64	0.37	1.02
**Unknown polar MB (838453006)**	Unknown	0.38	0.35	0.48	0.30	0.14	0.24
**Unknown polar MB (838453007)**	Unknown	0.19	0.21	0.12	0.21	0.09	0.21
**Unknown polar MB (838453009)**	Unknown	0.03	0.05	0.02	0.01	0.04	0.02
**Unknown polar MB (838453011)**	Unknown	0.87	0.40	0.26	0.63	0.17	0.35
**Unknown polar MB (838453012)**	Unknown	**187.01**	**293.71**	**97.92**	**164.94**	**136.84**	**55.31**
**Unknown polar MB (838453015)**	Unknown	0.20	0.20	0.35	0.27	0.15	0.80
**Unknown polar MB (838453017)**	Unknown	0.07	0.19	0.07	0.14	0.08	0.17
**Unknown polar MB (838453018)**	Unknown	0.10	0.20	0.12	0.18	0.10	0.34
**Unknown polar MB (838453019)**	Unknown	0.06	0.08	0.02	0.03	0.06	0.10
**Unknown polar MB (838453026)**	Unknown	0.23	0.14	0.14	0.14	0.04	0.09
**Unknown polar MB (838453028)**	Unknown	0.08	0.08	0.06	0.07	0.08	0.04
**Unknown polar MB (838453029)**	Unknown	0.08	0.11	0.13	0.09	0.11	0.35
**Unknown polar MB (838453030)**	Unknown	0.17	0.18	0.15	0.15	0.05	0.09
**Unknown polar MB (838453031)**	Unknown	0.01	0.02	0.01	0.01	0.01	0.01
**Unknown polar MB (838453033)**	Unknown	0.06	0.07	0.06	0.09	0.05	0.10
**Unknown polar MB (838453035)**	Unknown	0.08	0.03	0.01	0.05	0.03	0.02
**Unknown polar MB (838453036)**	Unknown	0.09	0.12	0.03	0.13	0.09	0.04
**Unknown polar MB (838453039)**	Unknown	0.08	0.05	0.09	0.08	0.12	0.56
**Unknown polar MB (838453040)**	Unknown	0.05	0.08	0.31	0.09	0.23	1.11
**Unknown polar MB (838453041)**	Unknown	0.18	0.21	0.33	0.19	0.25	0.57
**Unknown polar MB (838453043)**	Unknown	0.07	0.06	0.04	0.10	0.05	0.05
**Unknown polar MB (838453044)**	Unknown	0.31	0.28	0.77	0.62	0.65	1.09
**Unknown polar MB (838453051)**	Unknown	0.42	0.24	0.63	0.65	0.57	0.78
**Unknown polar MB (838453052)**	Unknown	0.21	0.12	0.26	0.28	0.21	0.42
**Unknown polar MB (838453053)**	Unknown	0.19	0.19	0.31	0.47	0.48	0.76
**Unknown polar MB (838453056)**	Unknown	0.31	0.15	0.34	0.44	0.36	0.53
**Unknown polar MB (838453057)**	Unknown	0.17	0.12	0.28	0.28	0.22	0.34
**Unknown polar MB (838453058)**	Unknown	0.65	0.07	0.10	1.55	0.19	0.38
**Unknown polar MB (838453059)**	Unknown	**2.95**	1.43	**2.87**	**3.57**	2.14	**2.23**
**Unknown polar MB (838453062)**	Unknown	0.50	0.42	0.66	0.76	0.66	0.80
**Unknown polar MB (838453063)**	Unknown	0.60	0.08	0.12	1.18	0.24	0.28
**Unknown polar MB (838453064)**	Unknown	0.14	0.09	0.17	0.39	0.19	0.30
**Unknown polar MB (838453066)**	Unknown	0.01	0.01	0.02	0.01	0.03	0.82
**Unknown polar MB (838453067)**	Unknown	**3.04**	**3.71**	**2.18**	**1.54**	**1.60**	**2.21**
**Unknown polar MB ESI neg (878450071)**	Unknown	**4.03**	1.87	**2.36**	**3.69**	**3.05**	**2.52**
**Unknown polar MB ESI pos (868450018)**	Unknown	0.20	0.19	0.40	0.27	0.16	0.63
**Unknown polar MB ESI pos (868450034)**	Unknown	0.34	0.37	0.72	0.39	0.39	0.63
**Uracil**	Nucleobases and related	0.17	0.29	0.24	0.30	0.32	0.34
**Xanthurenic acid**	Amino acids related	0.07	0.12	0.16	0.13	0.10	0.12

Table S2: Meropenem-induced feces metabolite fold changes in male (m) Wistar rats (N=5 per group) dosed with 100 (LD) and 300 (HD) mg/kg bw/day for 7, 14 and 28 days (m7, m14 and m28). Statistically significant changes (Welch-t-test; p-value < 0.05) are shown in bold numbers where red boxes mean a significant increase in the respective fecal metabolites and yellow a significant reduction compared to control levels.

		Meropenem trihydrate HD			Meropenem trihydrate LD		
**Metabolite**	**Class**	**m7**	**m14**	**m28**	**m7**	**m14**	**m28**
**1,25-Dihydroxy-vitamin D3**	Unknown	0.87	0.52	0.61	0.80	0.88	0.92
**1,2-Anhydroribose**	Carbohydrates and related	**4.14**	**5.01**	**5.10**	**4.93**	**4.12**	**3.49**
**1,4-Hydroquinone**	Miscellaneous	**2.89**	**3.03**	**2.05**	**1.57**	1.39	1.26
**14-Methylhexadecanoic acid**	Complex lipids, fatty acids and related	0.33	0.25	0.35	0.65	0.65	1.06
**15-Methylhexadecanoic acid**	Complex lipids, fatty acids and related	0.32	0.28	0.36	0.39	0.51	0.69
**2-Hydroxybehenic acid (C22:0)**	Complex lipids, fatty acids and related	0.42	0.38	0.50	0.55	0.71	0.98
**2-Hydroxycerotic acid (C26:0)**	Complex lipids, fatty acids and related	0.36	0.36	0.54	0.46	0.62	0.84
**2-Hydroxynervonic acid (C24:1)**	Complex lipids, fatty acids and related	0.35	0.37	0.47	0.45	0.72	0.91
**2-Hydroxypalmitic acid (C16:0)**	Complex lipids, fatty acids and related	0.54	0.41	0.56	0.67	0.71	0.82
**2-Hydroxypentacosanoic acid (C25:0)**	Complex lipids, fatty acids and related	0.39	0.40	0.45	0.51	0.59	0.73
**2-Methylserine**	Unknown	0.46	0.50	0.69	1.08	1.85	1.40
**3-Hydroxybutyrate**	Energy metabolism and related	**7.52**	**14.74**	**8.54**	**4.14**	**7.90**	**3.21**
**3-Hydroxyphenylacetic acid**	Amino acids related	0.09	0.13	0.06	0.05	0.06	0.03
**4-Hydroxyphenylacetic acid**	Amino acids related	**33.77**	**13.92**	**5.58**	**24.99**	4.27	4.17
**4-Hydroxysphinganine (t18:0, Phytosphingosine), total**	Complex lipids, fatty acids and related	**4.25**	**3.63**	**3.08**	**6.77**	**5.97**	**2.68**
**5-Oxoproline**	Amino acids related	**13.96**	**16.44**	**19.00**	**10.82**	**11.95**	**9.98**
**5-Oxoproline, lipid fraction**	Miscellaneous	**1.79**	**1.27**	**1.70**	1.60	1.48	**2.44**
**6-Hydroxynicotinic acid**	Vitamins, cofactors and related	**5.81**	**6.57**	**7.39**	**7.42**	**14.84**	**20.77**
**Adenine**	Nucleobases and related	0.20	0.29	0.38	0.26	0.25	0.37
**Allantoin**	Nucleobases and related	**239.76**	**134.81**	**291.73**	**373.34**	**23.50**	**63.32**
**alpha-Tocopherol**	Vitamins, cofactors and related	0.39	0.54	0.80	0.56	0.77	1.42
**alpha-Tocopherol**	Unknown	0.04	0.11	0.14	0.05	0.19	0.43
**alpha-Tocotrienol**	Unknown	0.07	0.22	0.20	0.08	0.49	0.58
**a-Muricholic Acid**	Unknown	**23.88**	2.53	**2.28**	**18.70**	**5.85**	**2.13**
**Arabinose**	Carbohydrates and related	**2.00**	2.87	**2.39**	**2.51**	4.44	**4.28**
**Arginine**	Amino acids	**3.81**	**1.78**	**2.48**	**3.48**	5.21	**3.18**
**Azelaic acid (Dicarboxylic acid; C9:0)**	Complex lipids, fatty acids and related	**2.12**	**2.59**	**2.03**	1.99	1.34	1.66
**Behenic acid (C22:0)**	Complex lipids, fatty acids and related	0.63	0.66	0.70	0.83	0.87	0.96
**beta-/gamma-Tocotrienol**	Unknown	0.48	0.61	0.82	0.53	0.80	1.28
**beta-/gamma-Tocotrienol**	Unknown	0.51	0.53	0.81	0.51	0.64	1.20

beta-Alanine	Amino acids related	4.59	4.80	18.76	19.75	10.88	31.31
beta-Muricholic acid	Complex lipids, fatty acids and related	38.79	8.83	13.25	23.11	3.08	15.92
beta-Sitosterol, total	Miscellaneous	3.22	3.78	3.30	3.13	3.78	2.08
Bile acid No 01	Complex lipids, fatty acids and related	23.07	2.97	4.41	16.88	2.88	4.34
Biliverdin	Unknown	0.16	0.08	0.21	0.38	0.13	0.22
bis-Glyceryl phosphate, polar fraction	Miscellaneous	3.48	4.54	2.00	2.40	1.82	1.34
b-Muricholic Acid	Unknown	8.16	2.43	2.13	5.03	1.73	3.48
Campesterol, total	Miscellaneous	3.15	3.33	2.91	3.04	3.20	1.85
Catechol	Miscellaneous	1.09	0.53	0.39	0.92	0.67	1.06
Cerebronic acid (2-OH-C24:0)	Complex lipids, fatty acids and related	0.44	0.41	0.54	0.57	0.72	0.86
Cholesterol, total	Complex lipids, fatty acids and related	2.65	3.03	2.36	3.15	3.64	1.26
Cholic acid	Unknown	13.78	22.75	33.78	4.61	5.80	7.61
Coenzyme Q10	Unknown	0.66	0.40	0.55	0.69	0.71	0.93
conjugated Linoleic acid (C18:trans[9,11]2)	Complex lipids, fatty acids and related	0.38	0.50	0.67	0.64	0.62	1.23
Cortisol (lipid)	Unknown	0.51	0.49	0.51	0.60	0.62	0.66
Creatinine	Amino acids related	102.63	101.53	103.74	104.49	84.80	123.58
Creatinine	Unknown	280.14	184.57	228.47	151.10	213.64	338.16
Cysteine	Amino acids	6.54	8.76	6.74	7.44	7.34	8.67
Cytosine	Nucleobases and related	3.38	6.81	4.32	2.18	1.62	1.52
Dehydroalanine	Amino acids related	7.23	7.84	5.47	7.48	5.68	4.24
Deoxycholic acid	Complex lipids, fatty acids and related	0.13	0.20	0.17	0.47	0.67	0.44
Deoxycholic acid	Unknown	0.01	0.01	0.00	0.20	0.21	0.24
Eicosadienoic acid (C20:2) No 01	Complex lipids, fatty acids and related	0.32	0.30	0.25	0.43	0.48	0.49
Eicosadienoic acid (C20:2) No 02	Complex lipids, fatty acids and related	0.30	0.22	0.28	0.51	0.42	0.43
Eicosaenoic acid (C20:1) No 02	Complex lipids, fatty acids and related	0.66	0.46	0.56	0.99	0.87	0.66
Eicosanoic acid (C20:0)	Complex lipids, fatty acids and related	0.67	0.57	0.63	0.68	0.70	0.87
epsilon-Acetyllysine	Unknown	6.71	6.62	5.43	8.39	10.46	9.97
erythro-Dihydrosphingosine (d18:0)	Complex lipids, fatty acids and related	0.22	0.20	0.30	0.29	0.49	0.50
Fucose	Carbohydrates and related	21.56	6.28	3.41	4.56	2.05	2.73
Fucosterol, total	Miscellaneous	2.71	2.28	2.65	2.77	2.06	1.91
Fumarate	Energy metabolism and related	4.26	6.04	4.69	3.15	3.07	2.93
Galactose	Carbohydrates and related	6.15	4.66	1.67	5.00	2.63	4.56
Galactose, lipid fraction	Complex lipids, fatty acids and related	3.18	1.79	2.27	2.72	1.98	2.23
gamma-Aminobutyrate (GABA)	Hormones, signal substances and related	5.00	4.37	5.37	3.52	2.32	3.76

**gamma-Tocopherol**	Vitamins, cofactors and related	0.50	0.57	0.79	0.63	0.67	0.94
**gamma-Tocopherol**	Unknown	0.59	0.81	1.32	0.76	0.81	1.65
**Glucose, lipid fraction**	Complex lipids, fatty acids and related	**5.24**	**2.03**	1.91	**3.58**	**3.35**	**2.13**
**Glucose-1-phosphate**	Carbohydrates and related	**6.74**	**6.31**	**3.17**	**3.47**	**3.50**	**2.73**
**Glucuronic acid**	Carbohydrates and related	**11.67**	**5.94**	**4.98**	**2.63**	2.60	**2.57**
**Glutamate**	Amino acids	**9.18**	**10.92**	**18.45**	**7.76**	**12.61**	**9.88**
**Glutamate**	Unknown	2.50	**3.76**	**3.40**	3.57	**5.97**	**4.82**
**Glutamic acid 2TMS ME (lipid)**	Unknown	**3.60**	**2.67**	**2.77**	**2.96**	**2.23**	**4.55**
**Glutamine**	Amino acids	**6.52**	**5.98**	**5.34**	**6.15**	4.63	**5.14**
**Glutarate**	Amino acids related	0.40	0.34	0.19	0.38	0.47	0.43
**Glycerol, lipid fraction**	Complex lipids, fatty acids and related	1.44	**2.68**	**2.27**	1.81	**3.15**	**3.01**
**Glycine, lipid fraction**	Miscellaneous	0.91	0.68	0.72	1.02	0.81	1.15
**Glycine, lipid fraction**	Miscellaneous	0.69	0.53	0.63	0.62	0.76	1.07
**Glycolate**	Miscellaneous	**3.90**	**3.38**	**2.73**	**3.12**	2.17	1.53
**Guanine**	Nucleobases and related	**2.55**	**1.48**	**1.80**	1.21	1.60	1.74
**Heneicosanoic acid (C21:0)**	Complex lipids, fatty acids and related	0.51	0.44	0.42	0.66	0.58	0.68
**Heptacosanoic acid (C27:0)**	Complex lipids, fatty acids and related	0.75	0.53	0.85	0.83	0.82	1.30
**Heptadecanoic acid (C17:0)**	Complex lipids, fatty acids and related	0.38	0.28	0.41	0.41	0.42	0.61
**Hexadecanol**	Complex lipids, fatty acids and related	0.06	0.09	0.10	0.06	0.08	0.38
**Hyodeoxycholic acid**	Unknown	0.02	0.00	0.00	0.01	0.00	0.01
**Hyodeoxycholic acid**	Complex lipids, fatty acids and related	0.32	0.09	0.26	0.09	0.04	0.22
**Hypoxanthine**	Nucleobases and related	**3.22**	**3.24**	2.47	**4.14**	2.76	1.27
**Indole-3-acetic acid**	Amino acids related	**15.87**	**28.71**	**46.83**	**7.58**	**6.44**	**32.59**
**Inosine**	Nucleobases and related	**6.02**	**8.05**	**4.53**	**5.43**	**11.36**	**7.05**
**isoLCA**	Unknown	0.01	0.01	0.01	0.14	0.19	0.14
**Isopalmitic acid (C16:0)**	Complex lipids, fatty acids and related	0.36	0.25	0.25	0.60	0.56	0.80
**Kynurenic acid**	Unknown	**6.60**	**2.56**	**5.58**	**6.67**	**2.95**	**5.91**
**Lignoceric acid (C24:0)**	Complex lipids, fatty acids and related	0.70	0.71	0.75	1.08	1.04	0.95
**Linoleic acid (C18:cis[9,12]2)**	Complex lipids, fatty acids and related	**3.60**	**5.01**	**5.28**	**3.29**	**3.50**	**8.33**
**Linolenic acid (C18:cis[9,12,15]3)**	Complex lipids, fatty acids and related	**4.15**	**6.57**	**5.73**	**5.24**	**4.81**	**9.50**
**Lithocholic acid**	Unknown	0.02	0.03	0.02	0.13	0.10	0.08
**Mannose**	Carbohydrates and related	**4.22**	**7.08**	**4.32**	**4.64**	4.11	**4.40**
**Methionine**	Unknown	0.51	0.11	0.31	0.51	0.66	0.96
**myo-Inositol**	Carbohydrates and related	**4.01**	**8.10**	**4.93**	**9.68**	2.90	0.97
**Myristic acid (C14:0)**	Complex lipids, fatty acids and related	0.40	0.31	0.36	0.44	0.63	1.02
**N-Acetyl-aspartic acid**	Unknown	**5.97**	**9.55**	**14.46**	**4.18**	2.49	2.88

**N-Acetylglucosamine**	Unknown	0.33	0.21	0.12	0.45	0.48	0.79
**N-Acetyl-leucine**	Unknown	**3.50**	**6.69**	3.32	1.25	0.66	1.42
**N-Acetylserine**	Unknown	**5.94**	**4.24**	1.62	**4.23**	**5.55**	1.55
**Nervonic acid (C24:cis[15]1)**	Complex lipids, fatty acids and related	0.41	0.39	0.64	0.71	0.61	0.94
**Niacinamide (Nicotinamide)**	Unknown	0.72	0.80	0.63	1.06	1.48	1.03
**Nicotinic acid**	Vitamins, cofactors and related	**2.29**	**3.44**	2.24	1.30	1.29	1.47
**Norleucine**	Miscellaneous	0.82	0.15	0.49	0.60	0.23	0.61
**Octadecanol**	Complex lipids, fatty acids and related	0.08	0.09	0.17	0.10	0.13	0.52
**Oleic acid (C18:cis[9]1)**	Complex lipids, fatty acids and related	0.14	0.07	0.22	0.30	0.30	0.55
**o-Muricholic Acid**	Unknown	0.35	0.05	0.05	0.14	0.15	0.07
**Pentacosanoic acid (C25:0)**	Complex lipids, fatty acids and related	0.62	0.53	0.74	0.82	0.79	0.85
**Pentadecanol**	Complex lipids, fatty acids and related	0.49	0.43	0.55	0.55	0.73	0.80
**Phosphate, lipid fraction**	Complex lipids, fatty acids and related	1.35	0.45	0.70	0.70	0.72	1.21
**Phosphoenolpyruvate (PEP)**	Energy metabolism and related	**5.39**	**6.03**	**4.69**	**3.62**	**3.11**	**2.42**
**Pipecolic acid**	Unknown	0.46	0.37	0.48	0.79	0.76	0.61
**plausible 18:2-Lyso PC (Na-Addukt)**	Unknown	**2.52**	**4.43**	**4.56**	**2.29**	2.69	2.89
**plausible 2-Hydroxyarachidic acid (2-OH-C20:0) TMS ME**	Unknown	0.37	0.31	0.42	0.55	0.60	0.85
**plausible 2-Hydroxytricosanoic acid (2-OH-C23:0) TMS ME**	Unknown	0.47	0.33	0.50	0.61	0.71	0.98
**plausible Heptadecanol TMS**	Unknown	0.01	0.02	0.03	0.01	0.20	0.70
**plausible SM (d18:1, C16:0) Na-Addukt**	Unknown	**4.69**	**2.72**	**3.21**	**4.47**	**2.30**	1.89
**Proline betaine**	Unknown	**2.12**	0.91	**2.40**	1.01	1.13	1.25
**putative 12-Methylmyristic acid TMS**	Unknown	0.03	0.06	0.04	0.04	0.26	0.33
**putative Anhydroglucuronate 4TMS**	Unknown	**4.49**	**4.72**	**2.32**	**2.49**	**3.03**	**2.23**
**putative beta-Muricholic acid 3TMS ME (Byprodukt)**	Unknown	**23.53**	**8.29**	**6.11**	**16.73**	7.59	**8.37**
**putative Bis-(glycerol)-phosphate**	Unknown	0.31	0.21	0.34	0.30	0.79	1.24
**putative C16:1 ME**	Unknown	1.14	**1.67**	**1.86**	**1.51**	**2.71**	**3.10**
**putative Choline plasmalogen (C36:2)**	Unknown	**3.14**	**3.20**	**3.83**	**3.80**	1.61	1.64
**putative Deoxycholylglycine deriv #1**	Unknown	0.01	0.01	0.01	0.22	0.40	0.24
**putative Hydroxy-eicosan OTMS**	Unknown	0.28	0.26	0.49	0.35	0.38	0.64
**putative Hyodeoxycholic acid 3TMS**	Unknown	0.02	0.01	0.08	0.02	0.01	0.10
**putative Lithocholic acid OTMS ME**	Unknown	0.05	0.04	0.04	0.19	0.25	0.07
**putative MeOx impurity (Blank)**	Unknown	**3.15**	**2.53**	**3.11**	**2.81**	2.35	**3.76**
**putative Tetracosenoic acid ME**	Unknown	0.72	0.36	0.54	1.21	1.08	0.70
**Putrescine**	Miscellaneous	**69.43**	**9.90**	**10.03**	**54.78**	15.85	**12.00**
**Pyruvate**	Energy metabolism and related	2.68	**3.79**	**3.01**	**1.64**	**2.10**	**1.82**
**scyllo-Inositol**	Carbohydrates and related	**18.20**	**17.92**	**25.01**	**16.03**	6.98	3.70
**Serine, lipid fraction**	Miscellaneous	0.74	0.68	0.68	0.96	0.62	0.86
**Shikimic acid**	Miscellaneous	**1068.12**	**939.60**	**1052.99**	**1018.27**	5.83	2.43

**Stearic acid (C18:0)**	Complex lipids, fatty acids and related	0.43	0.38	0.60	0.45	0.46	0.66
**Stigmastanol, total**	Miscellaneous	0.65	0.76	0.74	0.62	0.80	0.84
**Stigmasterol, total**	Miscellaneous	2.67	2.24	2.22	2.31	2.15	1.93
**Taurine**	Unknown	29.16	28.19	21.63	42.26	42.48	23.26
**Tauro-b-muricholic Acid**	Unknown	3.50	26.50	12.70	10.01	12.34	2.03
**Taurocholic acid**	Unknown	13.14	7.95	8.36	22.60	9.78	5.10
**Taurocholic Acid 3-sulfate**	Unknown	15.24	921.65	113.19	83.21	759.51	0.13
**Taurocholic acid sodium salt**	Unknown	4.49	110.41	12.63	8.93	19.40	0.33
**Tetradecanol**	Complex lipids, fatty acids and related	0.18	0.08	0.13	0.20	0.15	0.40
**Thiamine (Vitamin B1)**	Unknown	0.18	0.17	0.22	0.22	0.28	0.29
**threo-Sphingosine (d18:1)**	Complex lipids, fatty acids and related	1.74	1.48	2.17	2.41	2.82	2.54
**trans-4-Hydroxyproline**	Amino acids related	3.96	3.72	3.51	4.77	4.90	2.63
**trans-Ferulic acid**	Miscellaneous	6.02	9.98	10.35	2.58	2.80	3.50
**trans-Sinapic acid**	Miscellaneous	26.17	60.98	43.96	14.85	12.97	8.36
**Tricosanoic acid (C23:0)**	Complex lipids, fatty acids and related	0.57	0.49	0.56	0.79	0.67	0.87
**Tryptophan**	Amino acids	51.60	40.29	30.61	2.58	3.42	4.78
**Tyramine**	Amino acids related	34.30	15.97	21.26	8.00	8.15	6.30
**Tyrosine**	Unknown	0.32	0.16	0.24	0.65	0.56	0.41
**Ubichinone-30 (Coenzyme Q6)**	Unknown	0.69	0.45	0.70	0.74	0.78	0.87
**Ubichinone-35 (Coenzyme Q7)**	Unknown	0.71	0.39	0.60	0.78	0.67	0.80
**Ubichinone-45 (Coenzyme Q9)**	Unknown	0.84	0.43	0.66	0.80	0.76	0.80
**Unknown lipid MB (828450473)**	Unknown	5.14	2.96	2.15	2.74	2.81	3.30
**Unknown lipid MB (828451241)**	Unknown	0.10	0.04	0.15	0.11	0.13	0.40
**Unknown lipid MB (828451312)**	Unknown	0.20	0.49	0.15	0.75	1.09	0.44
**Unknown lipid MB (828451316)**	Unknown	2.28	3.75	2.36	2.11	1.94	3.67
**Unknown lipid MB (828451380)**	Unknown	0.75	0.39	0.50	1.09	0.94	0.95
**Unknown lipid MB (828451443)**	Unknown	0.70	0.64	0.68	0.74	0.95	1.02
**Unknown lipid MB (828451444)**	Unknown	0.68	0.44	0.57	1.44	1.04	0.81
**Unknown lipid MB (828451445)**	Unknown	0.32	0.31	0.41	0.38	0.44	0.63
**Unknown lipid MB (828451931)**	Unknown	0.01	0.01	0.04	0.01	0.32	1.60
**Unknown lipid MB (828451933)**	Unknown	0.10	0.05	0.17	0.09	0.13	0.31
**Unknown lipid MB (828451937)**	Unknown	0.00	0.00	0.02	0.01	0.18	0.55
**Unknown lipid MB (828451940)**	Unknown	0.00	0.00	0.02	0.00	0.16	0.51
**Unknown lipid MB (828452038)**	Unknown	0.02	0.02	0.02	0.03	0.16	0.93
**Unknown lipid MB (828452040)**	Unknown	0.57	0.19	0.25	0.25	0.31	0.55
**Unknown lipid MB (828452041)**	Unknown	0.02	0.03	0.09	0.01	0.19	0.70
**Unknown lipid MB (828452042)**	Unknown	0.00	0.00	0.00	NA	0.01	0.01
**Unknown lipid MB (828452043)**	Unknown	0.00	0.01	0.05	0.00	0.19	0.60
**Unknown lipid MB (828452044)**	Unknown	0.71	0.46	0.48	0.97	1.00	1.28
**Unknown lipid MB (828452047)**	Unknown	0.01	0.01	0.06	0.01	0.21	0.53

**Unknown lipid MB (828452050)**	Unknown	0.03	0.05	0.05	NA	0.19	0.52
**Unknown lipid MB (828452059)**	Unknown	0.37	0.07	0.04	0.38	0.11	0.04
**Unknown lipid MB ESI neg (858450034)**	Unknown	0.49	0.47	0.39	0.59	0.55	0.53
**Unknown lipid MB ESI neg (858450047)**	Unknown	**57.21**	**26.21**	**21.38**	**98.40**	**23.37**	**4.49**
**Unknown lipid MB ESI neg (858450068)**	Unknown	0.48	0.48	0.57	0.58	0.72	0.61
**Unknown lipid MB ESI neg (858450071)**	Unknown	**4.73**	**6.17**	**4.29**	**3.81**	**3.32**	**5.15**
**Unknown lipid MB ESI pos (848450056)**	Unknown	**4.17**	**8.14**	**5.98**	**5.54**	**5.29**	**6.06**
**Unknown lipid MB ESI pos (848450065)**	Unknown	**2.49**	**3.20**	3.68	**2.54**	1.46	2.16
**Unknown lipid MB ESI pos (848450067)**	Unknown	**4.18**	**3.90**	**2.31**	**2.17**	**1.74**	1.62
**Unknown lipid MB ESI pos (848450068)**	Unknown	**2.86**	**6.16**	**4.11**	**2.82**	2.68	4.19
**Unknown lipid MB ESI pos (848450069)**	Unknown	**2.90**	**5.19**	**5.29**	**3.19**	3.54	5.48
**Unknown lipid MB ESI pos (848450083)**	Unknown	**4.11**	**3.76**	1.89	**7.86**	**3.74**	1.47
**Unknown lipid MB ESI pos (848450084)**	Unknown	**2.54**	**3.06**	**2.54**	**3.81**	**3.69**	2.20
**Unknown lipid MB ESI pos (848450091)**	Unknown	0.44	0.44	1.37	0.62	0.53	0.69
**Unknown lipid MB ESI pos (848450092)**	Unknown	0.37	0.36	0.27	0.31	0.51	0.35
**Unknown lipid MB ESI pos (848450094)**	Unknown	**4.35**	**2.99**	**6.29**	**2.61**	1.37	1.55
**Unknown lipid MB ESI pos (848450096)**	Unknown	**2.72**	**3.72**	**2.70**	**1.85**	**2.54**	**2.93**
**Unknown lipid MB ESI pos (848450097)**	Unknown	**9.25**	**9.08**	**6.92**	**4.24**	**1.92**	**2.00**
**Unknown lipid MB ESI pos (848450100)**	Unknown	**4.32**	**5.37**	**3.63**	**3.01**	1.64	1.82
**Unknown lipid MB ESI pos (848450102)**	Unknown	**4.06**	**3.13**	**2.96**	**4.82**	0.75	1.26
**Unknown lipid MB ESI pos (848450103)**	Unknown	**4.12**	**4.85**	**3.26**	**4.88**	1.52	1.37
**Unknown lipid MB ESI pos (848450105)**	Unknown	**11.97**	**15.26**	**5.77**	**9.49**	**2.50**	**2.56**
**Unknown lipid MB ESI pos (848450106)**	Unknown	**7.04**	**3.90**	**3.07**	**4.84**	**3.14**	1.24
**Unknown lipid MB ESI pos (848450107)**	Unknown	1.80	**2.79**	**2.07**	**2.39**	2.31	**1.64**
**Unknown lipid MB ESI pos (848450108)**	Unknown	**3.91**	**4.07**	**3.33**	**4.48**	**3.14**	**2.23**
**Unknown lipid MB ESI pos (848450109)**	Unknown	**2.41**	**2.46**	**2.65**	**3.39**	**1.86**	1.80
**Unknown lipid MB ESI pos (848450110)**	Unknown	**11.76**	**6.96**	**5.32**	**6.78**	**4.08**	**2.65**
**Unknown lipid MB ESI pos (848450111)**	Unknown	**2.47**	**5.61**	**3.61**	**1.67**	**3.22**	**3.87**
**Unknown lipid MB ESI pos (848450112)**	Unknown	**2.93**	**2.96**	2.99	**3.03**	**2.42**	3.00
**Unknown lipid MB ESI pos (848450113)**	Unknown	**3.95**	**7.50**	**5.33**	**3.71**	**5.00**	**6.14**

**Unknown lipid MB ESI pos (848450114)**	Unknown	**4.58**	**4.96**	**4.71**	**5.12**	**3.08**	**2.21**
**Unknown lipid MB ESI pos (848450115)**	Unknown	**2.41**	**2.39**	1.90	**2.55**	1.56	1.05
**Unknown lipid MB ESI pos (848450116)**	Unknown	**6.69**	**6.06**	**4.23**	**6.23**	**6.30**	2.15
**Unknown lipid MB ESI pos (848450117)**	Unknown	**3.60**	**2.38**	2.27	**2.05**	0.94	0.88
**Unknown lipid MB ESI pos (848450119)**	Unknown	**2.39**	**2.37**	1.59	**1.72**	0.89	0.87
**Unknown lipid MB ESI pos (848450120)**	Unknown	**2.16**	**2.24**	2.34	**2.41**	1.38	1.29
**Unknown lipid MB ESI pos (848450129)**	Unknown	**2.42**	**2.86**	**2.55**	**3.43**	**2.53**	1.96
**Unknown lipid MB ESI pos (848450131)**	Unknown	**4.19**	**5.11**	**3.70**	**7.98**	**3.95**	**2.09**
**Unknown lipid MB ESI pos (848450132)**	Unknown	**3.89**	**3.33**	**2.94**	**5.29**	**2.85**	2.28
**Unknown lipid MB ESI pos (848450138)**	Unknown	**4.18**	**3.72**	**3.77**	**4.65**	**2.80**	**2.01**
**Unknown lipid MB ESI pos (848450139)**	Unknown	**5.07**	**8.69**	**4.45**	**6.92**	2.24	0.86
**Unknown lipid MB ESI pos (848450142)**	Unknown	**7.33**	**8.84**	**5.28**	**7.29**	3.16	1.39
**Unknown lipid MB ESI pos (848450144)**	Unknown	**3.93**	**6.92**	**5.65**	**4.72**	**5.39**	**3.22**
**Unknown lipid MB ESI pos (848450146)**	Unknown	**4.06**	**4.74**	**3.57**	**3.63**	**3.61**	**2.25**
**Unknown lipid MB ESI pos (848450147)**	Unknown	1.70	**2.23**	**2.43**	**1.96**	**1.87**	**1.68**
**Unknown lipid MB ESI pos (848450148)**	Unknown	**6.10**	**6.93**	**5.04**	**5.57**	**6.39**	**2.88**
**Unknown lipid MB ESI pos (848450170)**	Unknown	**2.03**	**1.30**	**1.50**	**2.68**	**2.36**	**2.15**
**Unknown lipid MB ESI pos (848450196)**	Unknown	**9.25**	**11.95**	**11.50**	**5.64**	**13.15**	**13.02**
**Unknown lipid MB ESI pos (848450201)**	Unknown	**8.90**	**8.63**	**7.76**	**6.68**	**10.57**	**10.63**
**Unknown lipid MB ESI pos (848450202)**	Unknown	**3.56**	**2.98**	**3.65**	**2.90**	**2.98**	**5.25**
**Unknown lipid MB ESI pos (848450204)**	Unknown	**11.65**	**11.73**	**10.89**	**8.19**	**12.97**	**15.65**
**Unknown lipid MB ESI pos (848450205)**	Unknown	**11.02**	**10.05**	**9.95**	**7.30**	**12.51**	**11.84**
**Unknown lipid MB ESI pos (848450206)**	Unknown	**11.32**	**18.01**	**14.71**	**9.45**	**20.31**	**18.86**
**Unknown lipid MB ESI pos (848450207)**	Unknown	**12.55**	**15.29**	**11.81**	**10.28**	**14.15**	**15.43**
**Unknown lipid MB ESI pos (848450208)**	Unknown	**10.39**	**11.59**	**9.44**	**7.81**	**9.48**	**13.32**
**Unknown lipid MB ESI pos (848450209)**	Unknown	**8.03**	**8.21**	**5.99**	**6.48**	**7.95**	**7.37**
**Unknown lipid MB ESI pos (848450210)**	Unknown	**8.10**	**8.90**	**9.38**	**5.61**	**7.56**	**13.21**
**Unknown lipid MB ESI pos (848450212)**	Unknown	**7.65**	**8.92**	**10.09**	**7.13**	**9.75**	**10.53**
**Unknown lipid MB ESI pos (848450213)**	Unknown	**9.43**	**10.98**	**11.18**	**8.34**	**12.68**	**13.78**

**Unknown lipid MB ESI pos (848450214)**	Unknown	8.04	8.99	10.15	8.08	11.04	12.10
**Unknown lipid MB ESI pos (848450215)**	Unknown	8.97	11.85	10.56	7.88	11.29	15.26
**Unknown lipid MB ESI pos (848450216)**	Unknown	8.52	10.11	9.01	7.81	10.34	10.38
**Unknown lipid MB ESI pos (848450218)**	Unknown	9.06	9.49	10.72	8.20	8.92	11.99
**Unknown lipid MB ESI pos (848450219)**	Unknown	7.96	8.99	9.37	6.47	9.22	10.36
**Unknown polar MB (838450146)**	Unknown	3.71	2.96	1.90	1.78	2.07	1.33
**Unknown polar MB (838450379)**	Unknown	0.83	0.70	0.65	1.00	0.94	0.98
**Unknown polar MB (838450566)**	Unknown	1.81	3.38	1.67	2.81	1.15	1.06
**Unknown polar MB (838450590)**	Unknown	0.64	0.14	0.40	0.39	0.27	0.58
**Unknown polar MB (838450618)**	Unknown	2.55	2.41	1.94	1.67	1.66	0.97
**Unknown polar MB (838450621)**	Unknown	13.33	10.16	12.53	5.95	3.91	1.97
**Unknown polar MB (838450692)**	Unknown	0.16	0.19	0.23	0.17	0.34	0.23
**Unknown polar MB (838450761)**	Unknown	4.27	2.81	2.59	3.94	2.53	1.09
**Unknown polar MB (838450807)**	Unknown	2.07	2.19	1.88	2.13	2.14	2.41
**Unknown polar MB (838450816)**	Unknown	0.01	0.00	0.00	0.01	0.00	0.01
**Unknown polar MB (838450990)**	Unknown	1.38	1.42	1.33	1.07	0.90	0.91
**Unknown polar MB (838451614)**	Unknown	0.02	0.10	0.03	0.02	0.05	0.02
**Unknown polar MB (838452021)**	Unknown	0.41	0.53	0.55	0.50	0.60	0.62
**Unknown polar MB (838452022)**	Unknown	2.74	2.81	2.47	2.24	2.18	1.98
**Unknown polar MB (838452079)**	Unknown	2.13	2.19	2.06	3.22	1.90	2.25
**Unknown polar MB (838452178)**	Unknown	0.12	0.09	0.27	0.17	0.29	0.53
**Unknown polar MB (838452196)**	Unknown	3.25	2.15	2.01	2.39	1.55	1.21
**Unknown polar MB (838452482)**	Unknown	380.41	161.12	245.70	397.56	27.10	85.99
**Unknown polar MB (838452999)**	Unknown	11.51	43.16	22.59	14.18	18.98	6.97
**Unknown polar MB (838453005)**	Unknown	0.23	0.19	0.17	0.13	0.24	0.23
**Unknown polar MB (838453006)**	Unknown	0.21	0.23	0.32	0.15	0.17	0.29
**Unknown polar MB (838453009)**	Unknown	0.06	0.03	0.02	0.04	0.02	0.03
**Unknown polar MB (838453012)**	Unknown	130.85	71.03	52.54	217.83	77.71	78.91
**Unknown polar MB (838453015)**	Unknown	0.18	0.07	0.27	0.20	0.22	0.32
**Unknown polar MB (838453017)**	Unknown	0.12	0.09	0.07	0.05	0.07	0.19
**Unknown polar MB (838453018)**	Unknown	0.10	0.09	0.15	0.07	0.23	0.39
**Unknown polar MB (838453019)**	Unknown	0.06	0.04	0.03	0.14	0.08	0.02
**Unknown polar MB (838453020)**	Unknown	1.86	1.62	2.09	1.58	2.79	1.48
**Unknown polar MB (838453026)**	Unknown	0.13	0.17	0.21	0.17	0.12	0.19
**Unknown polar MB (838453028)**	Unknown	0.11	0.06	0.04	0.07	0.04	0.05
**Unknown polar MB (838453033)**	Unknown	0.13	0.05	0.03	0.05	0.04	0.06
**Unknown polar MB (838453036)**	Unknown	0.03	0.02	0.03	0.05	0.06	0.04
**Unknown polar MB (838453040)**	Unknown	0.05	0.03	0.03	0.05	0.14	0.11
**Unknown polar MB (838453041)**	Unknown	0.12	0.10	0.12	0.14	0.11	0.24
**Unknown polar MB (838453044)**	Unknown	0.46	0.29	0.37	0.48	0.58	0.81
**Unknown polar MB (838453047)**	Unknown	0.20	0.08	0.15	0.25	0.10	0.18
**Unknown polar MB (838453048)**	Unknown	0.15	0.11	0.06	0.31	0.16	0.12
**Unknown polar MB (838453051)**	Unknown	0.41	0.48	0.53	0.71	0.71	0.71

**Unknown polar MB (838453052)**	Unknown	0.41	0.19	0.15	0.37	0.61	0.31
**Unknown polar MB (838453053)**	Unknown	0.28	0.22	0.19	0.48	1.88	0.66
**Unknown polar MB (838453055)**	Unknown	0.16	0.08	0.03	0.07	0.09	0.02
**Unknown polar MB (838453056)**	Unknown	0.51	0.41	0.19	0.48	0.32	0.32
**Unknown polar MB (838453057)**	Unknown	0.47	0.37	0.24	0.31	0.21	0.32
**Unknown polar MB (838453058)**	Unknown	0.58	0.07	0.05	0.43	0.08	0.23
**Unknown polar MB (838453059)**	Unknown	**3.50**	**3.10**	**2.86**	**3.09**	**3.14**	1.63
**Unknown polar MB (838453061)**	Unknown	0.83	0.71	0.66	0.64	0.75	0.68
**Unknown polar MB (838453062)**	Unknown	0.42	0.55	0.57	0.58	0.84	0.75
**Unknown polar MB (838453063)**	Unknown	0.55	0.03	0.05	0.20	0.05	0.09
**Unknown polar MB (838453066)**	Unknown	0.01	0.00	0.01	0.01	0.00	0.04
**Unknown polar MB (838453067)**	Unknown	**5.07**	**5.05**	**2.26**	**2.17**	**1.92**	**1.56**
**Unknown polar MB ESI neg (878450071)**	Unknown	**2.74**	**3.70**	**3.55**	**2.80**	2.44	**3.36**
**Uracil**	Nucleobases and related	0.17	0.06	0.17	0.25	0.14	0.33
**Xanthurenic acid**	Amino acids related	0.19	0.10	0.10	0.09	0.09	0.08
**Xylose**	Carbohydrates and related	**5.98**	**11.63**	**2.78**	1.64	1.64	1.43

Table S3: Doripenem-induced feces metabolite fold changes in female (f) Wistar rats (N=5 per group) dosed with 100 (LD) and 300 (HD) mg/kg bw/day for 7, 14 and 28 days (f7, f14 and f28). Statistically significant changes (Welch-t-test; p-value < 0.05) are shown in bold numbers where red boxes mean a significant increase in the respective fecal metabolites and yellow a significant reduction compared to control levels.

		Doripenem HD			Doripenem LD		
**Metabolite**	**Class**	**f7**	**f14**	**f28**	**f7**	**f14**	**f28**
**1,25-Dihydroxy-vitamin D3**	Unknown	0.74	0.78	0.59	1.06	1.31	1.24
**1,2-Anhydroribose**	Carbohydrates and related	**6.30**	**4.09**	**3.44**	**3.55**	**2.59**	**2.18**
**1,4-Hydroquinone**	Miscellaneous	**2.27**	**1.79**	**1.65**	**1.47**	0.96	1.15
**14-Methylhexadecanoic acid**	Complex lipids, fatty acids and related	0.30	0.16	0.09	0.17	0.42	1.07
**15-Methylhexadecanoic acid**	Complex lipids, fatty acids and related	0.27	0.26	0.24	0.30	0.30	1.02
**16-Methylheptadecanoic acid**	Complex lipids, fatty acids and related	0.68	0.33	0.28	0.66	0.91	1.09
**17-Methyloctadecanoic acid**	Complex lipids, fatty acids and related	0.61	0.44	0.28	0.51	0.78	0.98
**22-Hydroxydocosanoic acid (C22:0)**	Complex lipids, fatty acids and related	0.64	0.58	0.63	1.01	0.96	1.10
**2-Aminoadipic acid**	Amino acids related	**2.67**	**4.58**	**2.26**	**1.46**	**2.79**	**2.97**
**2-Hydroxybehenic acid (C22:0)**	Complex lipids, fatty acids and related	0.27	0.28	0.26	0.58	0.78	0.96
**2-Hydroxycerotic acid (C26:0)**	Complex lipids, fatty acids and related	0.27	0.31	0.26	0.52	0.71	0.79
**2-Hydroxynervonic acid (C24:1)**	Complex lipids, fatty acids and related	0.22	0.27	0.28	0.48	0.91	0.81
**2-Hydroxypalmitic acid (C16:0)**	Complex lipids, fatty acids and related	0.25	0.21	0.16	0.44	0.66	0.63
**2-Hydroxypentacosanoic acid (C25:0)**	Complex lipids, fatty acids and related	0.29	0.31	0.28	0.51	0.70	0.84
**3-Hydroxybutyrate**	Energy metabolism and related	**24.71**	**10.25**	**9.09**	1.60	1.11	**3.20**
**3-Hydroxyphenylacetic acid**	Amino acids related	0.07	0.01	0.09	0.07	0.04	0.11

**4-Hydroxyphenylacetic acid**	Amino acids related	6.56	0.39	0.14	**30.02**	**22.12**	**11.68**
**4-Hydroxysphinganine (t18:0, Phytosphingosine), total**	Complex lipids, fatty acids and related	**3.66**	**2.42**	**1.72**	**7.71**	**3.76**	**3.11**
**5-Oxoproline**	Amino acids related	**12.19**	**13.25**	**4.83**	**8.43**	**13.13**	**11.16**
**6-Hydroxynicotinic acid**	Vitamins, cofactors and related	**1.95**	**2.42**	**4.16**	**6.18**	**4.12**	**22.90**
**Adenine**	Nucleobases and related	0.41	0.25	0.37	0.25	0.21	0.32
**Adenosine**	Nucleobases and related	0.42	0.20	0.47	0.44	0.45	0.57
**Alanine**	Amino acids	0.39	**7.97**	**3.68**	0.47	0.57	1.07
**Allantoin**	Nucleobases and related	**87.29**	**59.67**	**67.06**	**222.29**	**291.94**	**69.80**
**alpha-Amyrin TMS**	Unknown	0.47	0.42	0.43	0.61	0.67	0.64
**alpha-Tocopherol**	Vitamins, cofactors and related	0.42	0.67	0.65	0.92	1.06	1.45
**alpha-Tocopherol**	Unknown	0.04	0.12	0.10	0.35	0.04	0.06
**alpha-Tocotrienol**	Unknown	0.08	0.21	0.18	0.44	0.05	0.09
**Arabinose**	Carbohydrates and related	**10.44**	**2.94**	**2.22**	1.67	**1.71**	**2.54**
**Arginine**	Amino acids	1.40	**3.87**	**4.73**	1.53	1.42	**2.71**
**Arginine**	Amino acids	**2.04**	**5.16**	**3.06**	0.72	**2.15**	**2.38**
**Behenic acid (C22:0)**	Complex lipids, fatty acids and related	0.55	0.60	0.52	0.70	0.90	0.93
**beta-/gamma-Tocotrienol**	Unknown	0.46	0.68	0.66	0.71	0.76	1.12
**beta-/gamma-Tocotrienol**	Unknown	0.44	0.72	0.58	0.66	0.72	0.93
**beta-Amyrin TMS**	Unknown	0.50	0.45	0.53	0.65	0.63	0.81
**beta-Sitosterol, total**	Miscellaneous	**2.65**	**4.31**	**3.90**	**3.57**	**3.12**	**3.29**
**Bile acid No 01**	Complex lipids, fatty acids and related	0.50	0.55	0.10	**2.76**	1.46	0.59
**Bile acid No 07**	Complex lipids, fatty acids and related	0.37	0.17	0.18	0.56	0.45	0.49
**Campesterol, total**	Miscellaneous	**2.67**	**3.68**	**3.04**	**3.66**	**3.06**	**2.64**
**Catechol**	Miscellaneous	0.66	0.56	0.42	0.98	0.62	0.91
**Cerebronic acid (2-OH-C24:0)**	Complex lipids, fatty acids and related	0.30	0.35	0.28	0.58	0.86	0.92
**Cerotic acid (C26:0)**	Complex lipids, fatty acids and related	0.46	0.48	0.58	0.54	0.79	1.02
**Cholestenol No 02**	Complex lipids, fatty acids and related	0.30	0.31	0.34	0.64	0.69	0.89
**Cholesterol, total**	Complex lipids, fatty acids and related	**3.22**	**2.51**	1.54	**5.21**	**2.44**	**2.15**
**Cholic acid**	Unknown	**3.44**	**2.81**	**5.60**	**15.50**	**15.76**	**15.61**
**Citrulline**	Unknown	0.58	**4.80**	**6.89**	0.37	0.37	0.44
**Coenzyme Q10**	Unknown	0.47	0.42	0.43	0.83	0.72	0.98
**conjugated Linoleic acid (C18:trans[9,11]2)**	Complex lipids, fatty acids and related	0.36	0.41	0.36	0.54	0.95	1.65
**Cortisol (lipid)**	Unknown	0.31	0.27	0.24	0.52	0.51	0.72
**Creatinine**	Amino acids related	**41.91**	**133.99**	**109.17**	**124.25**	**223.10**	**135.03**
**Creatinine**	Unknown	**80.68**	**155.80**	**90.00**	**49.47**	**191.42**	**109.27**
**Cysteine**	Amino acids	**4.35**	**4.37**	**4.22**	**4.35**	**5.20**	**7.60**

**Cytosine**	Nucleobases and related	**9.15**	**8.87**	**7.63**	**4.94**	**5.05**	**2.05**
**Dehydroalanine**	Amino acids related	**6.02**	**4.74**	**3.60**	**3.88**	**4.28**	**5.56**
**Deoxycholic acid**	Complex lipids, fatty acids and related	0.17	0.07	0.10	0.19	0.20	0.33
**Deoxycholic acid**	Unknown	0.00	0.00	0.00	0.01	0.00	0.03
**dihomo-gamma-Linolenic acid (C20:cis[8,11,14]3)**	Complex lipids, fatty acids and related	0.43	0.25	0.20	0.58	1.04	**2.76**
**Dihydrocholesterol**	Complex lipids, fatty acids and related	0.97	**1.45**	**1.57**	0.96	**1.16**	**2.76**
**Eicoasenoic acid (C20:cis[11]1)**	Complex lipids, fatty acids and related	0.52	0.58	1.00	0.76	0.70	1.37
**Eicosadienoic acid (C20:2) No 01**	Complex lipids, fatty acids and related	0.30	0.28	0.25	0.41	0.43	0.45
**Eicosadienoic acid (C20:2) No 02**	Complex lipids, fatty acids and related	0.30	0.29	0.24	0.35	0.45	0.51
**Eicosaenoic acid (C20:1) No 02**	Complex lipids, fatty acids and related	0.32	0.16	0.18	0.28	0.48	0.44
**Eicosanoic acid (C20:0)**	Complex lipids, fatty acids and related	0.54	0.53	0.51	0.64	0.82	0.92
**epsilon-Acetyllysine**	Unknown	**4.17**	**4.33**	**5.55**	**5.29**	**5.29**	**8.96**
**erythro-Dihydrosphingosine (d18:0)**	Complex lipids, fatty acids and related	0.12	0.17	0.16	0.21	0.32	1.14
**Fucose**	Carbohydrates and related	**36.76**	**8.26**	**4.32**	5.22	2.51	2.05
**Fucosterol, total**	Miscellaneous	**1.96**	**3.09**	**2.65**	**2.87**	**2.60**	**2.61**
**Fumarate**	Energy metabolism and related	**2.95**	**2.89**	1.74	3.44	**3.29**	**2.36**
**Fumarate**	Unknown	0.37	0.54	0.60	0.42	0.42	0.73
**Galactose**	Carbohydrates and related	**7.98**	**2.32**	0.85	**2.82**	**2.53**	1.45
**Galactose, lipid fraction**	Complex lipids, fatty acids and related	1.42	**1.85**	**1.88**	**2.59**	**1.62**	**1.90**
**Galactose, lipid fraction**	Complex lipids, fatty acids and related	0.59	0.76	0.78	1.01	0.66	0.96
**gamma-Tocopherol**	Vitamins, cofactors and related	0.56	0.47	0.32	0.62	0.64	0.75
**Glucose-1-phosphate**	Carbohydrates and related	**15.46**	**8.15**	**2.99**	**5.27**	**6.59**	**3.28**
**Glucuronic acid**	Carbohydrates and related	**470.20**	**7.79**	**4.44**	**7.12**	1.58	1.49
**Glutamate**	Amino acids	**6.50**	**10.04**	**3.50**	**5.46**	**9.99**	**8.79**
**Glutamic acid 2TMS ME (lipid)**	Unknown	**1.97**	**1.76**	**1.55**	**1.64**	**1.82**	1.88
**Glutamine**	Amino acids	**13.58**	**16.96**	1.83	**3.82**	**3.61**	**4.11**
**Glutarate**	Amino acids related	0.34	0.20	0.32	0.46	0.45	0.56
**Glycerol phosphate, lipid fraction**	Complex lipids, fatty acids and related	0.30	0.36	0.24	0.70	0.47	0.76
**Glycine**	Amino acids	2.15	**24.22**	**6.24**	1.59	2.12	1.62
**Glycine, lipid fraction**	Miscellaneous	0.45	0.53	0.60	0.75	0.67	**1.26**
**Glycine, lipid fraction**	Miscellaneous	0.35	0.38	0.44	0.61	0.62	1.16
**Glycodeoxycholate**	Unknown	0.46	0.33	0.35	0.45	0.26	0.39
**Glycolithocholic Acid**	Unknown	0.20	0.13	0.06	0.56	0.09	0.29
**Heneicosanoic acid (C21:0)**	Complex lipids, fatty acids and related	0.33	0.34	0.31	0.41	0.50	0.77

**Heptacosanoic acid (C27:0)**	Complex lipids, fatty acids and related	0.61	0.42	0.44	0.76	0.98	0.94
**Heptadecanoic acid (C17:0)**	Complex lipids, fatty acids and related	0.18	0.17	0.18	0.23	0.30	0.64
**Hexadecanol**	Complex lipids, fatty acids and related	0.06	0.03	0.09	0.08	0.12	0.31
**Homoserine**	Amino acids related	0.77	**3.28**	**3.07**	0.55	0.38	0.59
**Hyocholic acid**	Complex lipids, fatty acids and related	0.42	0.41	0.07	0.70	0.54	0.36
**Hyodeoxycholic acid**	Unknown	0.00	0.00	0.00	0.03	0.00	0.01
**Hyodeoxycholic acid**	Complex lipids, fatty acids and related	0.11	0.08	0.25	0.10	0.28	0.92
**Hypoxanthine**	Nucleobases and related	**26.53**	**21.09**	**9.06**	**2.27**	1.29	1.45
**Indole-3-acetic acid**	Amino acids related	3.06	0.11	0.28	**12.33**	**5.43**	**10.64**
**isoLCA**	Unknown	0.00	0.00	0.01	0.01	0.00	0.02
**Isoleucine**	Amino acids	0.53	**51.30**	**43.33**	0.64	0.80	0.73
**Isoluecine + Leucine**	Unknown	0.31	**27.46**	**26.92**	0.34	0.40	0.61
**Isopalmitic acid (C16:0)**	Complex lipids, fatty acids and related	0.27	0.14	0.07	0.16	0.36	0.91
**Kynurenic acid**	Unknown	**2.18**	**1.74**	**2.52**	**5.53**	**4.14**	**7.20**
**Leucine**	Amino acids	0.43	**58.67**	**34.65**	0.47	0.66	0.57
**Lignoceric acid (C24:0)**	Complex lipids, fatty acids and related	0.51	0.41	0.41	0.65	0.68	0.91
**Linoleic acid (C18:cis[9,12]2)**	Complex lipids, fatty acids and related	**4.61**	**6.28**	**5.81**	**5.27**	**6.96**	**6.75**
**Linolenic acid (C18:cis[9,12,15]3)**	Complex lipids, fatty acids and related	**4.49**	**5.88**	**5.29**	**5.33**	**6.28**	**4.97**
**Lithocholic acid**	Unknown	0.01	0.01	0.01	0.02	0.01	0.03
**Lysine**	Amino acids	**17.78**	**23.19**	**12.50**	**6.07**	**4.28**	**20.50**
**Lyxose**	Carbohydrates and related	**8.47**	2.07	**1.44**	1.33	1.20	**1.79**
**Mannose**	Carbohydrates and related	**8.25**	**8.00**	**6.59**	**9.29**	**4.84**	0.30
**myo-Inositol**	Carbohydrates and related	**61.66**	**144.72**	**57.91**	**6.94**	**3.44**	0.97
**Myristic acid (C14:0)**	Complex lipids, fatty acids and related	0.30	0.16	0.23	0.26	0.21	0.98
**N2-Acetylhistidine**	Unknown	**4.57**	**10.54**	**6.29**	0.87	2.16	2.26
**N2-Acetyllysine**	Amino acids related	**8.85**	**6.33**	**4.23**	**7.06**	**4.18**	**20.09**
**N-Acetylglucosamine**	Unknown	0.08	0.07	0.05	0.26	0.52	0.32
**N-Acetyl-leucine**	Unknown	**4.15**	**2.81**	**9.62**	2.13	0.68	1.15
**Nervonic acid (C24:cis[15]1)**	Complex lipids, fatty acids and related	0.40	0.31	0.36	0.66	0.63	1.02
**Niacinamide (Nicotinamide)**	Unknown	0.25	0.49	0.25	0.85	1.64	0.90
**N-Methylglutamate**	Unknown	0.03	0.02	0.05	0.11	0.10	0.17
**Norleucine**	Miscellaneous	0.21	0.30	0.30	0.27	0.18	0.27
**Octadecadienoic acid No 01 (C18:2)**	Complex lipids, fatty acids and related	0.49	0.55	0.58	0.65	1.36	1.40

**Octadecanol**	Complex lipids, fatty acids and related	0.06	0.04	0.13	0.11	0.23	0.31
**Oleic acid (C18:cis[9]1)**	Complex lipids, fatty acids and related	**2.31**	**2.78**	**3.69**	**2.73**	**2.95**	**4.65**
**omega-Muricholic acid**	Complex lipids, fatty acids and related	0.28	0.17	0.08	0.62	0.53	0.34
**o-Muricholic Acid**	Unknown	0.01	0.00	0.01	0.28	0.08	0.08
**Ornithine**	Amino acids related	**8.75**	**40.87**	**25.09**	1.35	1.34	1.57
**Palmitic acid (C16:0)**	Complex lipids, fatty acids and related	0.71	0.55	0.82	1.04	0.58	1.32
**Pentacosanoic acid (C25:0)**	Complex lipids, fatty acids and related	0.44	0.44	0.29	0.55	0.80	0.78
**Pentadecanol**	Complex lipids, fatty acids and related	0.32	0.34	0.37	0.40	0.38	0.80
**Phenylalanine**	Amino acids	0.76	**73.34**	**45.48**	1.12	1.46	1.38
**Phenylalanine**	Unknown	2.34	**57.50**	**27.35**	1.16	1.41	1.22
**Phenylalanine**	Unknown	0.39	**44.07**	**28.21**	0.79	0.79	0.95
**Phenylalanine**	Unknown	0.41	**45.46**	**33.25**	0.66	0.85	1.22
**Phosphate, lipid fraction**	Complex lipids, fatty acids and related	0.40	0.30	0.38	0.55	0.53	0.74
**Phosphoenolpyruvate (PEP)**	Energy metabolism and related	**4.12**	**3.99**	**2.66**	**3.02**	**3.15**	**3.08**
**p-Hydroxybenzoic acid**	Vitamins, cofactors and related	0.20	0.32	0.24	0.29	0.70	0.41
**Pipecolic acid**	Unknown	0.14	0.12	0.07	0.96	0.63	1.29
**plausible 18:2-Lyso PC (Na-Addukt)**	Unknown	**2.22**	**2.80**	**3.19**	**5.08**	1.97	**1.55**
**plausible 2-Hydroxyarachidic acid (2-OH-C20:0) TMS ME**	Unknown	0.20	0.21	0.24	0.45	0.67	0.98
**plausible 2-Hydroxytricosanoic acid (2-OH-C23:0) TMS ME**	Unknown	0.27	0.28	0.27	0.56	0.77	0.85
**plausible Heptadecanol TMS**	Unknown	0.01	0.01	0.03	0.01	0.30	0.29
**plausible SalzCluster (C7H10O8Na3(35)Cl(37)Cl)**	Unknown	0.61	0.51	0.50	0.76	0.76	1.01
**plausible SM (d18:1, C16:0) Na-Addukt**	Unknown	**6.19**	**2.96**	2.44	**7.05**	**3.76**	**3.72**
**proposed Sphingomyelin (d18:2,C16:0)**	Unknown	**4.06**	**2.95**	**2.93**	**5.79**	**2.87**	**3.11**
**putative 12-Methylmyristic acid TMS**	Unknown	0.01	0.02	0.02	0.02	0.03	0.71
**putative 2-Oxobutanedioic acid hexoside 6TMS**	Unknown	0.31	0.27	0.15	2.14	**2.01**	1.06
**putative Anhydroglucuronate 4TMS**	Unknown	**12.74**	**7.39**	2.62	**4.17**	**5.94**	**3.24**
**putative Bis-(glycerol)-phosphate**	Unknown	0.08	0.10	0.06	0.08	0.15	2.19
**putative Choline plasmalogen (C36:2)**	Unknown	**7.41**	**2.35**	**2.78**	**6.63**	**1.63**	**2.22**
**putative Deoxycholylglycine deriv #1**	Unknown	0.01	0.02	0.01	0.03	0.02	0.10
**putative Hydroxy-eicosan OTMS**	Unknown	0.15	0.11	0.12	0.15	0.27	0.32
**putative Hyodeoxycholic acid 3TMS**	Unknown	0.03	0.04	0.08	0.05	0.03	0.08
**putative Lithocholic acid OTMS ME**	Unknown	0.01	0.01	0.01	0.02	0.01	0.05
**putative Tetracosenoic acid ME**	Unknown	0.39	0.28	0.20	0.40	0.83	0.71
**Putrescine**	Miscellaneous	**11.68**	**13.36**	3.91	**43.96**	**39.65**	**18.12**

**Pyruvate**	Energy metabolism and related	3.88	3.33	3.19	2.45	2.05	2.20
**ribo-4-Hydroxy-8E-sphingenine**	Complex lipids, fatty acids and related	0.53	0.59	0.82	1.06	1.04	2.01
**Sarcosine**	Amino acids related	0.59	0.76	0.61	0.89	0.78	0.91
**scyllo-Inositol**	Carbohydrates and related	14.34	29.21	33.55	10.30	8.54	2.19
**Serine, lipid fraction**	Miscellaneous	0.56	0.53	0.50	0.66	0.59	1.08
**Serine, lipid fraction**	Miscellaneous	0.56	0.52	0.43	0.67	0.51	1.03
**Shikimic acid**	Miscellaneous	911.27	1238.06	1542.15	2535.91	937.46	3.07
**Stearic acid (C18:0)**	Complex lipids, fatty acids and related	0.59	0.44	0.44	0.74	0.55	0.72
**Stigmasterol, total**	Miscellaneous	1.82	2.79	2.89	2.67	2.38	2.92
**Taurine**	Unknown	3.84	8.87	8.09	10.20	12.97	21.63
**Tauro-b-muricholic Acid**	Unknown	6.80	4.47	2.27	2.02	1.59	1.73
**Taurochenodeoxycholate**	Unknown	1.65	1.94	1.95	1.63	1.18	1.87
**Taurocholic acid**	Unknown	2.99	3.73	3.64	1.68	1.35	2.43
**Taurocholic Acid 3-sulfate**	Unknown	395.06	302.06	28.52	139.94	156.93	16.82
**Taurocholic acid sodium salt**	Unknown	8.10	6.65	5.78	3.83	2.12	2.46
**Taurodeoxycholate**	Unknown	0.54	0.46	0.49	0.55	0.48	0.46
**Taurolithocholic Acid**	Unknown	0.03	0.02	0.01	0.20	0.03	0.40
**Tetradecanol**	Complex lipids, fatty acids and related	0.12	0.07	0.07	0.17	0.12	0.23
**Thiamine (Vitamin B1)**	Unknown	0.17	0.17	0.17	0.20	0.20	0.84
**threo-Sphinganine 3TMS**	Unknown	0.09	0.13	0.08	0.30	0.29	2.80
**trans-4-Hydroxyproline**	Amino acids related	1.88	16.09	43.62	2.92	2.56	3.23
**trans-Ferulic acid**	Miscellaneous	11.47	6.67	9.87	6.69	2.46	3.59
**trans-Ferulic acid, lipid fraction**		0.68	0.44	0.88	0.78	0.70	1.04
**trans-Sinapic acid**	Miscellaneous	29.91	24.05	23.51	17.57	8.25	4.22
**Tricosanoic acid (C23:0)**	Complex lipids, fatty acids and related	0.38	0.44	0.37	0.63	0.82	0.92
**Tryptophan**	Amino acids	59.51	90.26	59.46	2.53	1.56	2.57
**Tyramine**	Amino acids related	150.42	340.94	198.95	7.30	7.00	11.89
**Tyrosine**	Unknown	0.21	0.60	0.43	0.25	0.29	0.61
**Ubichinone-30 (Coenzyme Q6)**	Unknown	0.59	0.60	0.51	0.96	0.79	0.96
**Ubichinone-35 (Coenzyme Q7)**	Unknown	0.51	0.58	0.53	0.94	0.76	1.14
**Ubichinone-45 (Coenzyme Q9)**	Unknown	0.57	0.59	0.53	0.97	0.75	0.96
**Unknown lipid MB (828450473)**	Unknown	3.39	3.22	2.84	4.77	3.01	2.71
**Unknown lipid MB (828450676)**	Unknown	0.70	0.82	0.77	0.79	0.87	0.81
**Unknown lipid MB (828450834)**	Unknown	0.27	0.23	0.17	0.53	0.71	0.88
**Unknown lipid MB (828451241)**	Unknown	0.06	0.13	0.16	0.08	0.11	0.86
**Unknown lipid MB (828451259)**	Unknown	0.48	0.23	0.25	0.72	0.95	0.91
**Unknown lipid MB (828451275)**	Unknown	0.36	0.32	0.45	0.40	0.33	0.24
**Unknown lipid MB (828451312)**	Unknown	0.31	0.30	0.39	1.46	0.35	1.07
**Unknown lipid MB (828451316)**	Unknown	0.35	0.58	0.49	3.93	3.60	1.48

**Unknown lipid MB (828451380)**	Unknown	0.55	0.44	0.28	0.60	1.05	0.93
**Unknown lipid MB (828451443)**	Unknown	0.48	0.63	0.72	0.93	0.78	0.98
**Unknown lipid MB (828451444)**	Unknown	0.59	0.39	0.26	0.72	1.04	1.10
**Unknown lipid MB (828451445)**	Unknown	0.20	0.15	0.21	0.28	0.28	0.71
**Unknown lipid MB (828451931)**	Unknown	0.00	0.00	0.01	0.01	0.60	0.36
**Unknown lipid MB (828451933)**	Unknown	0.06	0.14	0.13	0.15	0.11	0.68
**Unknown lipid MB (828451937)**	Unknown	0.00	0.00	0.02	0.02	0.44	0.31
**Unknown lipid MB (828451940)**	Unknown	0.00	0.00	0.01	0.01	0.25	0.27
**Unknown lipid MB (828452038)**	Unknown	0.01	0.01	0.01	0.01	0.01	1.53
**Unknown lipid MB (828452040)**	Unknown	0.02	0.09	0.06	0.13	0.27	1.10
**Unknown lipid MB (828452041)**	Unknown	0.03	0.04	0.05	0.03	0.31	0.31
**Unknown lipid MB (828452042)**	Unknown	0.01	0.01	0.00	0.01	0.01	0.01
**Unknown lipid MB (828452043)**	Unknown	0.01	0.01	0.01	0.02	0.34	0.13
**Unknown lipid MB (828452047)**	Unknown	0.00	0.00	0.02	0.02	0.45	0.32
**Unknown lipid MB (828452048)**	Unknown	0.68	0.54	0.38	0.76	0.92	0.81
**Unknown lipid MB (828452050)**	Unknown	0.02	0.05	0.04	0.04	0.42	0.55
**Unknown lipid MB (828452058)**	Unknown	0.08	0.07	0.07	0.08	0.11	0.21
**Unknown lipid MB (828452059)**	Unknown	0.17	0.10	0.05	0.58	0.16	0.16
**Unknown lipid MB (828452060)**	Unknown	0.01	0.00	0.00	0.01	0.00	0.04
**Unknown lipid MB ESI neg (858450013)**	Unknown	0.64	**12.6 2**	**9.52**	1.03	1.03	0.86
**Unknown lipid MB ESI neg (858450034)**	Unknown	0.22	0.54	0.06	0.48	0.85	0.17
**Unknown lipid MB ESI neg (858450042)**	Unknown	0.30	0.24	0.23	**1.97**	2.11	**3.03**
**Unknown lipid MB ESI neg (858450047)**	Unknown	**6.89**	**9.37**	**4.62**	**2.24**	1.50	1.54
**Unknown lipid MB ESI neg (858450065)**	Unknown	0.02	0.03	0.01	0.09	0.07	0.07
**Unknown lipid MB ESI neg (858450068)**	Unknown	0.25	0.33	0.25	0.37	0.68	0.62
**Unknown lipid MB ESI neg (858450071)**	Unknown	**4.39**	**4.98**	**3.30**	**4.29**	**2.68**	**2.86**
**Unknown lipid MB ESI pos (848450056)**	Unknown	**5.12**	**3.81**	**4.99**	**5.45**	**3.54**	**2.30**
**Unknown lipid MB ESI pos (848450067)**	Unknown	**2.29**	**2.61**	**3.03**	**3.38**	**2.32**	**2.26**
**Unknown lipid MB ESI pos (848450068)**	Unknown	**3.71**	**2.67**	**2.23**	**4.74**	**1.72**	**1.46**
**Unknown lipid MB ESI pos (848450069)**	Unknown	**5.82**	**1.92**	**2.50**	**5.53**	1.46	1.49
**Unknown lipid MB ESI pos (848450083)**	Unknown	**5.80**	**3.39**	1.25	**6.72**	**2.75**	1.58
**Unknown lipid MB ESI pos (848450092)**	Unknown	0.28	0.26	0.37	0.55	0.38	0.48
**Unknown lipid MB ESI pos (848450093)**	Unknown	0.21	0.22	0.48	0.57	0.68	0.88
**Unknown lipid MB ESI pos (848450095)**	Unknown	0.48	0.43	0.79	0.74	0.50	0.77
**Unknown lipid MB ESI pos (848450096)**	Unknown	**2.04**	**1.66**	**2.21**	**3.90**	**1.94**	**3.16**
**Unknown lipid MB ESI pos (848450097)**	Unknown	**6.50**	**6.70**	**5.20**	**7.12**	**3.98**	**3.18**
**Unknown lipid MB ESI pos (848450100)**	Unknown	**4.46**	**1.85**	1.32	**4.19**	**1.49**	1.48
**Unknown lipid MB ESI pos (848450102)**	Unknown	**4.76**	**2.89**	**2.78**	**6.85**	**2.33**	**1.73**
**Unknown lipid MB ESI pos (848450103)**	Unknown	**5.03**	**3.00**	**2.21**	**6.47**	**2.24**	**1.64**
**Unknown lipid MB ESI pos (848450105)**	Unknown	**11.7 2**	**6.54**	**6.28**	**12.6 9**	**4.98**	**3.71**
**Unknown lipid MB ESI pos (848450106)**	Unknown	**7.14**	**3.23**	1.72	**6.65**	**2.40**	1.57
**Unknown lipid MB ESI pos (848450107)**	Unknown	**2.94**	**2.05**	**2.36**	**3.59**	**1.76**	**1.86**
**Unknown lipid MB ESI pos (848450108)**	Unknown	**6.62**	**3.63**	**4.70**	**6.99**	**2.38**	**3.25**
**Unknown lipid MB ESI pos (848450109)**	Unknown	**5.68**	**1.74**	2.56	**6.35**	**1.97**	**2.71**
**Unknown lipid MB ESI pos (848450110)**	Unknown	**10.5 6**	**3.15**	**2.06**	**9.35**	**2.82**	**1.92**
**Unknown lipid MB ESI pos (848450111)**	Unknown	**2.91**	**2.92**	**3.14**	**3.70**	**3.33**	**3.74**
**Unknown lipid MB ESI pos (848450112)**	Unknown	**4.88**	**2.55**	**2.62**	**5.41**	1.38	**2.54**

**Unknown lipid MB ESI pos (848450113)**	Unknown	5.68	5.03	5.22	7.79	4.49	6.13
**Unknown lipid MB ESI pos (848450114)**	Unknown	8.85	4.78	4.39	10.51	2.13	3.53
**Unknown lipid MB ESI pos (848450123)**	Unknown	2.76	1.62	1.38	5.98	1.89	1.29
**Unknown lipid MB ESI pos (848450129)**	Unknown	3.09	2.43	1.56	4.66	2.73	2.96
**Unknown lipid MB ESI pos (848450131)**	Unknown	5.98	4.07	2.97	9.41	4.28	3.37
**Unknown lipid MB ESI pos (848450132)**	Unknown	5.35	2.86	1.66	8.51	3.52	2.74
**Unknown lipid MB ESI pos (848450138)**	Unknown	4.60	2.89	1.63	6.34	3.26	2.09
**Unknown lipid MB ESI pos (848450139)**	Unknown	7.32	6.01	1.91	5.91	2.13	1.20
**Unknown lipid MB ESI pos (848450142)**	Unknown	8.25	8.05	2.30	7.06	2.69	1.11
**Unknown lipid MB ESI pos (848450144)**	Unknown	4.24	3.20	1.80	5.35	3.16	2.48
**Unknown lipid MB ESI pos (848450146)**	Unknown	4.02	4.08	2.14	6.14	3.92	2.38
**Unknown lipid MB ESI pos (848450147)**	Unknown	2.09	1.66	1.34	2.68	2.61	1.63
**Unknown lipid MB ESI pos (848450148)**	Unknown	5.52	4.00	2.62	7.65	4.98	3.14
**Unknown lipid MB ESI pos (848450152)**	Unknown	2.77	2.35	1.08	4.72	2.75	2.07
**Unknown lipid MB ESI pos (848450154)**	Unknown	0.45	0.30	0.14	1.08	1.64	0.55
**Unknown lipid MB ESI pos (848450155)**	Unknown	0.58	0.41	0.25	1.82	1.59	1.85
**Unknown lipid MB ESI pos (848450159)**	Unknown	0.58	0.39	0.37	0.93	1.96	2.07
**Unknown lipid MB ESI pos (848450196)**	Unknown	14.95	16.93	10.97	11.66	14.33	9.58
**Unknown lipid MB ESI pos (848450201)**	Unknown	10.79	14.69	9.71	8.49	13.68	8.42
**Unknown lipid MB ESI pos (848450202)**	Unknown	3.83	4.57	4.69	3.68	4.88	4.15
**Unknown lipid MB ESI pos (848450204)**	Unknown	14.24	20.73	10.37	13.05	17.51	9.98
**Unknown lipid MB ESI pos (848450205)**	Unknown	13.63	13.70	10.10	11.78	11.57	8.38
**Unknown lipid MB ESI pos (848450206)**	Unknown	15.50	31.04	15.47	13.35	27.52	14.38
**Unknown lipid MB ESI pos (848450207)**	Unknown	15.96	24.50	14.33	12.73	20.62	12.64
**Unknown lipid MB ESI pos (848450208)**	Unknown	13.27	20.24	12.47	12.23	20.91	9.40
**Unknown lipid MB ESI pos (848450209)**	Unknown	8.46	9.92	8.19	7.93	9.65	7.07
**Unknown lipid MB ESI pos (848450210)**	Unknown	10.77	13.49	11.69	10.26	11.47	9.68
**Unknown lipid MB ESI pos (848450212)**	Unknown	8.59	15.80	7.01	8.58	13.86	7.07
**Unknown lipid MB ESI pos (848450213)**	Unknown	12.13	16.30	9.95	11.24	13.62	8.62
**Unknown lipid MB ESI pos (848450214)**	Unknown	9.87	16.94	7.73	9.64	17.00	7.56
**Unknown lipid MB ESI pos (848450215)**	Unknown	13.24	24.24	11.38	11.63	19.18	8.54
**Unknown lipid MB ESI pos (848450216)**	Unknown	10.80	13.62	8.49	9.93	11.41	7.88
**Unknown lipid MB ESI pos (848450218)**	Unknown	12.74	18.00	11.01	11.89	16.73	7.93
**Unknown lipid MB ESI pos (848450219)**	Unknown	9.23	14.20	10.45	9.33	11.38	8.03
**Unknown polar MB (838450146)**	Unknown	7.98	2.66	2.19	2.18	2.43	1.27
**Unknown polar MB (838450566)**	Unknown	0.39	0.32	0.38	1.26	0.87	1.18
**Unknown polar MB (838450590)**	Unknown	0.85	0.26	0.36	1.25	0.54	26.53

**Unknown polar MB (838450621)**	Unknown	**5.66**	**6.59**	**5.59**	**7.14**	**6.17**	1.54
**Unknown polar MB (838450677)**	Unknown	0.89	0.40	0.50	0.75	0.66	0.63
**Unknown polar MB (838450692)**	Unknown	0.14	0.08	0.06	0.17	0.24	0.23
**Unknown polar MB (838450807)**	Unknown	**2.21**	0.98	**1.42**	2.15	1.29	**2.66**
**Unknown polar MB (838450816)**	Unknown	0.01	0.01	0.00	0.02	0.01	0.01
**Unknown polar MB (838451614)**	Unknown	0.02	0.01	0.01	0.02	0.01	0.01
**Unknown polar MB (838452021)**	Unknown	0.37	0.34	0.34	0.47	0.60	0.64
**Unknown polar MB (838452022)**	Unknown	**2.07**	**1.60**	1.44	**1.59**	**1.61**	1.34
**Unknown polar MB (838452079)**	Unknown	**4.98**	**2.38**	**4.03**	**3.05**	**1.61**	**3.23**
**Unknown polar MB (838452178)**	Unknown	0.10	0.07	0.14	0.10	0.22	0.77
**Unknown polar MB (838452196)**	Unknown	**2.85**	**3.16**	**2.68**	**2.22**	**1.83**	**1.60**
**Unknown polar MB (838452482)**	Unknown	**36.89**	**25.06**	**38.26**	**208.70**	**241.92**	**118.91**
**Unknown polar MB (838452804)**	Unknown	**4.65**	**2.01**	2.10	1.16	0.46	**1.94**
**Unknown polar MB (838452999)**	Unknown	1.35	**6.79**	**13.59**	**34.86**	**41.30**	**10.01**
**Unknown polar MB (838453005)**	Unknown	0.26	0.09	0.15	0.07	0.11	0.32
**Unknown polar MB (838453006)**	Unknown	0.30	0.14	0.22	0.09	0.15	0.30
**Unknown polar MB (838453007)**	Unknown	0.10	0.16	0.14	0.17	0.31	0.25
**Unknown polar MB (838453008)**	Unknown	0.34	0.68	0.51	1.19	0.95	**1.44**
**Unknown polar MB (838453009)**	Unknown	0.02	0.03	0.01	0.03	0.03	0.02
**Unknown polar MB (838453011)**	Unknown	1.13	0.27	0.23	1.29	0.43	0.69
**Unknown polar MB (838453012)**	Unknown	**34.43**	**96.71**	**33.33**	**143.00**	**120.51**	**66.61**
**Unknown polar MB (838453014)**	Unknown	0.51	0.53	0.39	0.77	0.80	**1.44**
**Unknown polar MB (838453015)**	Unknown	0.13	0.19	0.24	0.22	0.27	0.85
**Unknown polar MB (838453017)**	Unknown	0.11	0.03	0.07	0.19	0.23	0.31
**Unknown polar MB (838453018)**	Unknown	0.07	0.04	0.05	0.12	0.36	0.33
**Unknown polar MB (838453019)**	Unknown	0.05	0.05	0.02	0.08	0.06	0.03
**Unknown polar MB (838453026)**	Unknown	0.18	0.07	0.05	0.29	0.12	0.14
**Unknown polar MB (838453028)**	Unknown	0.13	0.05	0.03	0.08	0.08	0.44
**Unknown polar MB (838453029)**	Unknown	0.10	0.05	0.08	0.10	0.09	0.35
**Unknown polar MB (838453030)**	Unknown	0.36	0.07	0.04	0.36	0.16	0.30
**Unknown polar MB (838453031)**	Unknown	0.00	0.00	0.01	0.01	0.01	0.24
**Unknown polar MB (838453033)**	Unknown	0.07	0.04	0.02	0.09	0.06	0.23
**Unknown polar MB (838453035)**	Unknown	0.19	0.03	0.01	0.10	0.05	0.07
**Unknown polar MB (838453036)**	Unknown	0.26	0.08	0.02	0.11	0.09	0.07
**Unknown polar MB (838453039)**	Unknown	0.03	0.02	0.02	0.10	0.04	0.07
**Unknown polar MB (838453040)**	Unknown	0.08	0.03	0.03	0.20	0.07	0.20
**Unknown polar MB (838453041)**	Unknown	0.13	0.12	0.18	0.19	0.15	0.61
**Unknown polar MB (838453043)**	Unknown	0.06	0.02	0.06	0.06	0.05	0.08
**Unknown polar MB (838453044)**	Unknown	0.19	0.13	0.18	0.25	0.35	0.86
**Unknown polar MB (838453045)**	Unknown	0.28	0.12	0.04	0.46	0.35	0.19
**Unknown polar MB (838453047)**	Unknown	0.13	0.08	0.10	0.18	0.14	0.72
**Unknown polar MB (838453050)**	Unknown	1.00	0.16	0.05	1.15	0.82	0.56
**Unknown polar MB (838453051)**	Unknown	0.29	0.37	0.36	0.37	0.58	0.67
**Unknown polar MB (838453052)**	Unknown	0.22	0.07	0.12	0.23	0.26	0.37
**Unknown polar MB (838453053)**	Unknown	0.25	0.10	0.13	0.31	0.24	0.44
**Unknown polar MB (838453056)**	Unknown	0.34	0.10	0.13	0.36	0.35	0.35
**Unknown polar MB (838453057)**	Unknown	0.35	0.11	0.18	0.28	0.31	0.58

**Unknown polar MB (838453058)**	Unknown	0.31	0.10	0.07	0.78	0.26	0.18
**Unknown polar MB (838453059)**	Unknown	**2.60**	**2.50**	**2.69**	**3.02**	2.30	**2.86**
**Unknown polar MB (838453062)**	Unknown	0.41	0.45	0.51	0.50	0.75	0.81
**Unknown polar MB (838453063)**	Unknown	0.27	0.04	0.02	0.60	0.22	0.22
**Unknown polar MB (838453064)**	Unknown	0.18	0.07	0.05	0.21	0.11	0.11
**Unknown polar MB (838453066)**	Unknown	0.01	0.01	0.01	0.01	0.01	0.68
**Unknown polar MB (838453067)**	Unknown	**2.20**	**2.27**	**2.64**	**3.08**	**2.23**	1.83
**Unknown polar MB ESI neg (878450071)**	Unknown	**3.16**	2.62	**3.26**	**3.48**	1.78	**1.85**
**Unknown polar MB ESI pos (868450018)**	Unknown	0.35	0.35	0.45	0.16	0.27	0.45
**Unknown polar MB ESI pos (868450034)**	Unknown	0.33	**1.98**	**3.07**	0.33	0.38	0.38
**Unknown polar MB ESI pos (868450040)**	Unknown	**4.89**	**9.87**	**5.61**	1.54	2.06	1.85
**Uric acid**	Nucleobases and related	**11.79**	**4.80**	2.76	0.55	1.04	2.38
**Valine**	Amino acids	0.48	**45.65**	**44.76**	0.64	0.83	1.01
**Valine TMS ME**	Unknown	0.61	**2.58**	**2.48**	0.84	0.86	0.99
**Valine, lipid fraction**	Miscellaneous	0.56	**2.13**	**1.90**	0.82	0.83	0.86
**Xanthine**	Nucleobases and related	**63.94**	**37.27**	**21.17**	0.29	0.42	0.74
**Xanthurenic acid**	Amino acids related	0.08	0.06	0.11	0.08	0.19	0.26
**Xylose**	Carbohydrates and related	**13.58**	**10.49**	**13.42**	**2.44**	1.74	2.34

Table S4: Doripenem-induced feces metabolite fold changes in male (m) Wistar rats (N=5 per group) dosed with 100 (LD) and 300 (HD) mg/kg bw/day for 7, 14 and 28 days (m7, m14 and m28). Statistically significant changes (Welch-t-test; p-value < 0.05) are shown in bold numbers where red boxes mean a significant increase in the respective fecal metabolites and yellow a significant reduction compared to control levels.

		Doripenem HD			Doripenem LD		
**Metabolite**	**Class**	**m7**	**m14**	**m28**	**m7**	**m14**	**m28**
**1,25-Dihydroxy-vitamin D3**	Unknown	0.74	0.32	0.37	0.71	0.78	0.91
**1,2-Anhydroribose**	Carbohydrates and related	**7.45**	**3.90**	**7.11**	**5.46**	**4.35**	**6.14**
**1,4-Hydroquinone**	Miscellaneous	**3.06**	**2.62**	**1.93**	**1.53**	1.27	1.20
**14-Methylhexadecanoic acid**	Complex lipids, fatty acids and related	0.34	0.15	0.30	0.31	0.34	0.36
**15-Methylhexadecanoic acid**	Complex lipids, fatty acids and related	0.33	0.25	0.33	0.24	0.31	0.28
**16-Methylheptadecanoic acid**	Complex lipids, fatty acids and related	0.69	0.28	0.43	0.49	0.59	0.50
**17-Methyloctadecanoic acid**	Complex lipids, fatty acids and related	0.67	0.37	0.52	0.61	0.62	0.61
**22-Hydroxydocosanoic acid (C22:0)**	Complex lipids, fatty acids and related	0.29	0.51	0.81	0.58	0.84	0.66
**2-Hydroxybehenic acid (C22:0)**	Complex lipids, fatty acids and related	0.40	0.26	0.34	0.36	0.56	0.46
**2-Hydroxycerotic acid (C26:0)**	Complex lipids, fatty acids and related	0.33	0.22	0.35	0.34	0.42	0.42
**2-Hydroxynervonic acid (C24:1)**	Complex lipids, fatty acids and related	0.36	0.25	0.36	0.27	0.51	0.43

**2-Hydroxypalmitic acid (C16:0)**	Complex lipids, fatty acids and related	0.43	0.30	0.39	0.42	0.71	0.61
**2-Hydroxypentacosanoic acid (C25:0)**	Complex lipids, fatty acids and related	0.33	0.28	0.33	0.32	0.41	0.35
**3-Hydroxybutyrate**	Energy metabolism and related	**22.45**	**21.35**	**12.66**	**2.67**	**2.51**	2.90
**3-Hydroxyphenylacetic acid**	Amino acids related	0.06	0.08	0.09	0.06	0.07	0.04
**3-Methylxanthine**	Miscellaneous	0.52	0.67	0.67	1.13	1.24	**1.70**
**4-Hydroxyphenylacetic acid**	Amino acids related	1.51	0.14	0.13	6.12	**10.37**	**9.62**
**4-Hydroxysphinganine (t18:0, Phytosphingosine), total**	Complex lipids, fatty acids and related	**4.36**	**2.70**	**1.64**	**3.75**	**6.81**	**3.11**
**5-Oxoproline**	Amino acids related	**19.63**	**14.11**	**12.26**	**10.45**	**12.85**	**14.77**
**6-Hydroxynicotinic acid**	Vitamins, cofactors and related	**2.05**	**3.13**	**3.85**	**5.03**	**5.05**	**6.95**
**Adenine**	Nucleobases and related	0.65	0.25	0.33	0.27	0.22	0.37
**Alanine**	Amino acids	4.58	**9.59**	**23.66**	0.91	0.40	1.24
**Allantoin**	Nucleobases and related	**126.77**	**20.82**	**36.90**	**295.35**	**235.87**	**660.66**
**alpha-Tocopherol**	Vitamins, cofactors and related	0.34	0.38	0.52	0.73	0.61	0.84
**alpha-Tocopherol**	Unknown	0.06	0.04	0.08	0.03	0.03	0.11
**alpha-Tocotrienol**	Unknown	0.06	0.09	0.15	0.05	0.05	0.13
**Arabinose**	Carbohydrates and related	**36.59**	4.70	**4.64**	**3.37**	2.78	**2.28**
**Arginine**	Amino acids	**11.49**	**8.33**	**10.43**	**2.53**	1.10	**2.03**
**Arginine**	Amino acids	**7.55**	**12.85**	**10.38**	1.93	1.42	**2.15**
**Behenic acid (C22:0)**	Complex lipids, fatty acids and related	0.62	0.44	0.53	0.74	0.63	0.60
**beta-/gamma-Tocotrienol**	Unknown	0.30	0.40	0.51	0.63	0.58	0.76
**beta-/gamma-Tocotrienol**	Unknown	0.29	0.38	0.48	0.67	0.51	0.63
**beta-Amyrin TMS**	Unknown	0.69	0.63	0.68	0.80	0.92	0.70
**beta-Sitosterol, total**	Miscellaneous	**2.30**	**2.69**	**2.62**	**2.88**	**3.21**	**2.75**
**Biliverdin**	Unknown	0.38	0.08	0.40	0.37	0.17	0.26
**bis-Glyceryl phosphate, polar fraction**	Miscellaneous	**2.72**	**2.21**	1.26	2.92	1.80	1.74
**Campesterol, total**	Miscellaneous	**2.36**	**2.02**	**2.62**	**2.69**	**2.86**	**2.34**
**Cerebronic acid (2-OH-C24:0)**	Complex lipids, fatty acids and related	0.41	0.33	0.37	0.39	0.53	0.44
**Cerotic acid (C26:0)**	Complex lipids, fatty acids and related	0.71	0.50	0.59	0.78	0.74	0.52
**Cholestenol No 02**	Complex lipids, fatty acids and related	0.53	0.39	0.48	0.69	0.55	0.64
**Cholic acid**	Unknown	**14.31**	**26.92**	**5.47**	**15.34**	**20.97**	14.38
**Coenzyme Q10**	Unknown	0.55	0.44	0.36	0.51	0.79	0.52
**conjugated Linoleic acid (C18:trans[9,11]2)**	Complex lipids, fatty acids and related	0.42	0.31	0.44	0.35	0.59	0.61
**Cortisol (lipid)**	Unknown	0.40	0.30	0.33	0.65	0.49	0.68
**Creatinine**	Amino acids related	**159.09**	**92.16**	**129.29**	**132.71**	**104.82**	**94.54**

**Creatinine**	Unknown	124.16	192.72	363.87	230.67	168.08	281.58
**Cysteine**	Amino acids	7.78	5.87	6.38	10.93	8.46	12.04
**Cytosine**	Nucleobases and related	8.35	7.28	10.43	3.71	3.95	4.19
**Dehydroalanine**	Amino acids related	8.23	5.28	4.64	7.97	5.45	6.31
**Deoxycholic acid**	Complex lipids, fatty acids and related	0.21	0.14	0.25	0.10	0.12	0.11
**Deoxycholic acid**	Unknown	0.01	0.00	0.01	0.01	0.00	0.00
**Eicoasenoic acid (C20:cis[11]1)**	Complex lipids, fatty acids and related	0.44	0.44	0.60	0.63	0.53	0.69
**Eicosadienoic acid (C20:2) No 01**	Complex lipids, fatty acids and related	0.22	0.23	0.20	0.21	0.30	0.26
**Eicosadienoic acid (C20:2) No 02**	Complex lipids, fatty acids and related	0.20	0.23	0.20	0.25	0.18	0.23
**Eicosaenoic acid (C20:1) No 02**	Complex lipids, fatty acids and related	0.47	0.23	0.30	0.48	0.63	0.34
**Eicosanoic acid (C20:0)**	Complex lipids, fatty acids and related	0.58	0.40	0.55	0.77	0.55	0.56
**epsilon-Acetyllysine**	Unknown	4.65	6.17	5.44	11.16	7.59	8.06
**erythro-Dihydrosphingosine (d18:0)**	Complex lipids, fatty acids and related	0.21	0.11	0.15	0.15	0.24	0.21
**Ethanolamine**	Miscellaneous	1.09	0.55	0.57	0.80	0.59	0.61
**Fucose**	Carbohydrates and related	31.75	35.39	15.54	4.27	5.43	3.51
**Fucosterol, total**	Miscellaneous	2.02	1.53	2.11	2.74	2.27	2.25
**Fumarate**	Energy metabolism and related	4.74	5.10	4.74	4.29	3.18	4.18
**Galactose, lipid fraction**	Complex lipids, fatty acids and related	1.73	1.70	1.19	1.43	1.81	1.71
**gamma-Tocopherol**	Vitamins, cofactors and related	0.34	0.30	0.45	0.44	0.50	0.46
**gamma-Tocopherol**	Unknown	0.47	0.64	1.01	0.82	0.65	0.91
**Glucose-1-phosphate**	Carbohydrates and related	23.01	4.62	12.54	3.93	6.00	5.07
**Glucuronic acid**	Carbohydrates and related	1788.81	23.41	296.33	4.38	5.14	4.37
**Glutamate**	Amino acids	10.10	8.07	7.04	7.07	7.42	16.06
**Glutamate**	Unknown	2.79	4.57	3.54	3.71	2.13	3.38
**Glutamic acid 2TMS ME (lipid)**	Unknown	2.65	2.17	2.25	1.80	1.64	2.64
**Glutamine**	Amino acids	57.36	41.04	38.02	5.82	3.35	4.46
**Glutarate**	Amino acids related	0.24	0.25	0.20	0.53	0.51	0.53
**Glycerol phosphate, lipid fraction**	Complex lipids, fatty acids and related	0.46	0.47	0.23	0.49	0.49	0.48
**Glycerol, polar fraction**	Complex lipids, fatty acids and related	3.04	7.56	5.14	1.07	0.93	1.65
**Glycine**	Amino acids	29.03	39.86	48.01	6.97	1.80	3.26
**Glycine, lipid fraction**	Miscellaneous	0.71	0.63	0.56	0.80	0.67	0.68
**Glycine, lipid fraction**	Miscellaneous	0.47	0.55	0.53	0.52	0.57	0.56
**Heneicosanoic acid (C21:0)**	Complex lipids, fatty acids and related	0.46	0.35	0.42	0.46	0.47	0.42

**Heptacosanoic acid (C27:0)**	Complex lipids, fatty acids and related	0.62	0.44	0.73	0.75	0.66	0.56
**Heptadecanoic acid (C17:0)**	Complex lipids, fatty acids and related	0.33	0.20	0.28	0.32	0.24	0.34
**Hexadecanol**	Complex lipids, fatty acids and related	0.06	0.05	0.12	0.04	0.23	0.15
**Homoserine**	Amino acids related	1.73	**2.76**	**4.43**	0.57	0.82	0.72
**Hyodeoxycholic acid**	Unknown	0.01	0.00	0.01	0.01	0.00	0.01
**Hyodeoxycholic acid**	Complex lipids, fatty acids and related	0.11	0.10	0.26	0.09	0.06	0.08
**Hypoxanthine**	Nucleobases and related	**51.96**	**39.24**	**21.50**	**3.36**	**2.51**	**3.04**
**Indole-3-acetic acid**	Amino acids related	0.17	0.21	0.32	**6.64**	**12.50**	**30.25**
**Inosine**	Nucleobases and related	3.23	**3.81**	**3.13**	**4.10**	**7.69**	**8.54**
**isoLCA**	Unknown	0.01	0.00	0.00	0.00	0.00	0.01
**Isoleucine**	Amino acids	72.14	**64.82**	**127.40**	0.68	0.46	0.83
**Isopalmitic acid (C16:0)**	Complex lipids, fatty acids and related	0.26	0.18	0.21	0.23	0.31	0.23
**Kynurenic acid**	Unknown	**3.05**	0.90	**2.15**	**5.06**	**4.06**	**6.87**
**Lactose**	Carbohydrates and related	**9.76**	**23.18**	3.01	1.09	0.75	1.49
**Leucine**	Amino acids	63.31	**58.79**	**121.57**	0.43	0.31	0.63
**Lignoceric acid (C24:0)**	Complex lipids, fatty acids and related	0.84	0.42	0.55	0.67	0.63	0.60
**Linoleic acid (C18:cis[9,12]2)**	Complex lipids, fatty acids and related	**2.62**	**2.94**	**3.85**	**3.50**	**4.09**	**3.57**
**Linolenic acid (C18:cis[9,12,15]3)**	Complex lipids, fatty acids and related	**2.89**	**4.09**	**4.18**	**3.77**	**6.02**	**3.99**
**Lithocholic acid**	Unknown	0.02	0.02	0.02	0.02	0.02	0.02
**Lysine**	Amino acids	**37.03**	**33.35**	**19.88**	**12.48**	3.31	**23.11**
**Lyxose**	Carbohydrates and related	**34.50**	5.17	**4.50**	2.59	2.08	0.89
**Mannose**	Carbohydrates and related	**25.43**	**4.69**	**7.05**	**4.44**	**4.51**	**6.15**
**myo-Inositol**	Carbohydrates and related	**299.98**	**350.58**	**152.31**	**3.96**	**7.51**	4.38
**Myristic acid (C14:0)**	Complex lipids, fatty acids and related	0.22	0.22	0.23	0.34	0.32	0.38
**N2-Acetylhistidine**	Unknown	**4.06**	**5.24**	**4.73**	**1.94**	0.89	1.33
**N-Acetyl-leucine**	Unknown	**4.32**	**9.27**	**16.52**	**4.43**	**3.05**	**8.96**
**Nervonic acid (C24:cis[15]1)**	Complex lipids, fatty acids and related	0.44	0.30	0.47	0.51	0.39	0.59
**Niacinamide (Nicotinamide)**	Unknown	0.34	0.26	0.16	0.61	0.76	0.40
**Norleucine**	Miscellaneous	0.44	0.19	0.51	0.44	0.15	0.38
**Octadecadienoic acid No 01 (C18:2)**	Complex lipids, fatty acids and related	0.49	0.38	0.40	0.41	0.70	0.67
**Octadecanol**	Complex lipids, fatty acids and related	0.06	0.06	0.15	0.08	0.53	0.23
**Oleic acid (C18:cis[9]1)**	Complex lipids, fatty acids and related	0.10	0.06	0.14	0.09	0.12	0.34
**o-Muricholic Acid**	Unknown	0.08	0.00	0.00	0.14	0.03	0.02

**Ornithine**	Amino acids related	**42.22**	**49.12**	**32.08**	3.16	1.15	1.73
**Palmitic acid (C16:0)**	Complex lipids, fatty acids and related	0.46	0.52	0.63	0.54	0.68	0.64
**Pentacosanoic acid (C25:0)**	Complex lipids, fatty acids and related	0.60	0.34	0.57	0.58	0.55	0.51
**Pentadecanol**	Complex lipids, fatty acids and related	0.40	0.37	0.45	0.49	0.44	0.42
**Phenylalanine**	Amino acids	**81.12**	**87.32**	**96.48**	1.74	0.91	1.30
**Phenylalanine**	Unknown	**59.92**	**45.66**	**52.36**	2.08	0.93	1.92
**Phenylalanine**	Unknown	**79.31**	**46.39**	**68.31**	1.25	0.83	1.15
**Phenylalanine**	Unknown	**76.92**	**39.36**	**63.46**	0.95	0.56	1.24
**Phenylalanine, lipid fraction**	Miscellaneous	2.04	**1.75**	**1.99**	0.78	0.67	0.56
**Phosphate, lipid fraction**	Complex lipids, fatty acids and related	0.46	0.33	0.34	0.42	0.47	0.55
**Phosphoenolpyruvate (PEP)**	Energy metabolism and related	**4.85**	**4.04**	**3.90**	**5.55**	**4.19**	**3.12**
**Pipecolic acid**	Unknown	0.23	0.16	0.08	0.69	0.63	0.61
**plausible 2-Hydroxyarachidic acid (2-OH-C20:0) TMS ME**	Unknown	0.31	0.22	0.30	0.35	0.54	0.39
**plausible 2-Hydroxytricosanoic acid (2-OH-C23:0) TMS ME**	Unknown	0.52	0.28	0.35	0.40	0.54	0.45
**plausible Heptadecanol TMS**	Unknown	0.01	0.01	0.02	0.01	0.04	0.04
**plausible SM (d18:1, C16:0) Na-Addukt**	Unknown	**4.05**	**3.35**	**2.15**	**2.99**	**4.18**	**3.15**
**Proline**	Amino acids	1.64	**12.01**	**34.01**	1.62	0.92	1.87
**Proline betaine**	Unknown	0.65	0.58	0.69	1.74	0.72	1.30
**putative 12-Methylmyristic acid TMS**	Unknown	0.04	0.02	0.03	0.03	NA	0.03
**putative Anhydroglucuronate 4TMS**	Unknown	**14.76**	**7.28**	**10.73**	**3.37**	**3.94**	**3.32**
**putative Bis-(glycerol)-phosphate**	Unknown	0.28	0.19	0.25	0.24	0.25	0.22
**putative Choline plasmalogen (C36:2)**	Unknown	**5.36**	**2.95**	**3.16**	2.97	**2.55**	2.06
**putative Deoxycholylglycine deriv #1**	Unknown	0.02	0.01	0.01	0.02	0.00	0.02
**putative Hydroxy-eicosan OTMS**	Unknown	0.26	0.17	0.28	0.30	0.40	0.30
**putative Hyodeoxycholic acid 3TMS**	Unknown	0.01	0.01	0.08	0.02	0.01	0.05
**putative Lithocholic acid OTMS ME**	Unknown	0.05	0.02	0.02	0.04	0.03	0.03
**putative Tetracosenoic acid ME**	Unknown	0.57	0.22	0.38	0.45	0.47	0.41
**Putrescine**	Miscellaneous	**11.27**	3.64	**6.43**	**65.37**	**13.15**	**26.08**
**Pyruvate**	Energy metabolism and related	**3.69**	**2.98**	**3.57**	**2.93**	**2.30**	**2.59**
**scyllo-Inositol**	Carbohydrates and related	**16.33**	**23.87**	**43.04**	**16.95**	**13.18**	**34.72**
**Serine**	Amino acids	47.19	**23.98**	**49.95**	1.25	0.82	1.07
**Serine, lipid fraction**	Miscellaneous	1.16	0.53	0.50	0.96	0.59	0.51
**Serine, lipid fraction**	Miscellaneous	1.06	0.51	0.59	0.82	0.59	0.52
**Shikimic acid**	Miscellaneous	**738.89**	**504.54**	**1196.46**	**1255.66**	**829.12**	**1575.29**
**Stearic acid (C18:0)**	Complex lipids, fatty acids and related	0.35	0.30	0.44	0.37	0.35	0.46

**Stigmastanol, total**	Miscellaneous	0.50	0.56	0.67	0.57	0.66	0.68
**Stigmasterol, total**	Miscellaneous	**1.69**	**1.42**	**2.09**	**2.02**	**1.71**	**1.87**
**Taurine**	Unknown	**24.18**	**16.93**	**10.51**	**21.25**	**19.93**	**23.17**
**Tauro-b-muricholic Acid**	Unknown	**20.19**	**148.81**	**40.80**	**4.03**	**6.14**	2.65
**Taurocholic acid**	Unknown	**74.24**	**81.28**	**34.24**	**6.05**	**11.30**	**5.20**
**Taurocholic Acid 3-sulfate**	Unknown	142.02	**1738.70**	**129.59**	**20.83**	85.11	43.38
**Taurocholic acid sodium salt**	Unknown	**16.62**	**73.02**	**11.51**	**6.14**	**15.13**	4.85
**Tetradecanol**	Complex lipids, fatty acids and related	0.17	0.09	0.11	0.17	0.15	0.12
**Thiamine (Vitamin B1)**	Unknown	0.30	0.09	0.42	0.30	0.15	0.21
**Threonine**	Amino acids	45.82	**50.83**	**87.07**	**2.42**	1.19	2.67
**trans-4-Hydroxyproline**	Amino acids related	**22.30**	**112.85**	**134.93**	**3.42**	2.43	1.78
**trans-Ferulic acid**	Miscellaneous	**6.56**	**7.27**	**9.78**	**8.31**	**7.20**	**7.57**
**trans-Sinapic acid**	Miscellaneous	**18.52**	**32.13**	**22.55**	**33.76**	**34.50**	**27.86**
**Tricosanoic acid (C23:0)**	Complex lipids, fatty acids and related	0.55	0.34	0.44	0.54	0.54	0.50
**Tryptophan**	Amino acids	**86.05**	**120.14**	**92.36**	**25.01**	2.39	8.98
**Tyramine**	Amino acids related	**401.80**	**132.62**	**317.71**	**180.74**	**9.83**	**11.80**
**Tyrosine**	Unknown	3.56	0.30	0.52	0.21	0.18	0.27
**Ubichinone-30 (Coenzyme Q6)**	Unknown	0.50	0.54	0.41	0.49	0.71	0.59
**Ubichinone-35 (Coenzyme Q7)**	Unknown	0.53	0.42	0.40	0.53	0.65	0.59
**Ubichinone-45 (Coenzyme Q9)**	Unknown	0.61	0.42	0.43	0.55	0.70	0.57
**Unknown lipid MB (828450473)**	Unknown	**4.78**	**2.90**	**2.80**	**3.19**	**3.02**	**2.22**
**Unknown lipid MB (828450676)**	Unknown	0.92	0.80	0.87	0.96	0.75	0.90
**Unknown lipid MB (828450834)**	Unknown	0.45	0.29	0.33	0.49	0.73	0.73
**Unknown lipid MB (828451241)**	Unknown	0.08	0.06	0.13	0.13	0.05	0.10
**Unknown lipid MB (828451316)**	Unknown	0.38	0.34	0.46	**2.01**	**2.47**	2.47
**Unknown lipid MB (828451380)**	Unknown	0.71	0.27	0.51	0.55	0.51	0.41
**Unknown lipid MB (828451443)**	Unknown	0.60	0.43	0.55	0.76	0.67	0.74
**Unknown lipid MB (828451444)**	Unknown	0.76	0.31	0.49	0.54	0.53	0.43
**Unknown lipid MB (828451445)**	Unknown	0.26	0.22	0.27	0.30	0.29	0.35
**Unknown lipid MB (828451931)**	Unknown	0.01	0.00	0.01	0.01	0.00	0.02
**Unknown lipid MB (828451933)**	Unknown	0.06	0.06	0.10	0.14	0.06	0.09
**Unknown lipid MB (828451937)**	Unknown	0.00	0.00	0.00	0.00	0.02	0.01
**Unknown lipid MB (828451940)**	Unknown	0.00	0.00	0.00	0.00	0.01	0.01
**Unknown lipid MB (828452038)**	Unknown	0.03	0.01	0.02	0.02	0.01	0.02
**Unknown lipid MB (828452040)**	Unknown	0.37	0.17	0.43	0.14	0.17	0.21
**Unknown lipid MB (828452041)**	Unknown	0.04	0.07	0.05	0.03	0.08	0.04
**Unknown lipid MB (828452042)**	Unknown	0.01	0.00	0.00	0.00	0.00	0.00
**Unknown lipid MB (828452043)**	Unknown	0.01	0.02	0.01	0.01	0.02	0.00
**Unknown lipid MB (828452044)**	Unknown	0.61	0.33	0.44	0.53	0.59	0.44
**Unknown lipid MB (828452047)**	Unknown	0.03	0.00	0.00	0.02	0.09	0.02
**Unknown lipid MB (828452048)**	Unknown	1.09	0.42	0.55	0.72	0.88	0.49

**Unknown lipid MB (828452053)**	Unknown	0.58	0.51	0.69	0.67	0.64	0.64
**Unknown lipid MB (828452059)**	Unknown	0.21	0.06	0.03	0.40	0.07	0.03
**Unknown lipid MB (828452060)**	Unknown	0.01	0.00	0.00	0.01	0.00	0.00
**Unknown lipid MB ESI neg (858450013)**	Unknown	**16.62**	**14.05**	**37.52**	0.74	0.39	1.14
**Unknown lipid MB ESI neg (858450034)**	Unknown	0.34	0.16	0.17	1.01	0.52	0.89
**Unknown lipid MB ESI neg (858450047)**	Unknown	**73.06**	**58.06**	**56.50**	**54.25**	**26.79**	**6.91**
**Unknown lipid MB ESI neg (858450065)**	Unknown	0.31	0.16	0.28	1.39	0.86	1.54
**Unknown lipid MB ESI neg (858450068)**	Unknown	0.42	0.36	0.31	0.48	0.60	0.77
**Unknown lipid MB ESI neg (858450071)**	Unknown	**2.45**	2.07	**2.75**	**3.71**	**3.22**	**2.94**
**Unknown lipid MB ESI pos (848450056)**	Unknown	**5.57**	**3.80**	**4.60**	**5.63**	**4.95**	**4.79**
**Unknown lipid MB ESI pos (848450083)**	Unknown	**4.20**	**2.83**	1.64	**3.02**	**3.41**	1.62
**Unknown lipid MB ESI pos (848450084)**	Unknown	**2.51**	**3.10**	2.14	**2.04**	**2.99**	**2.46**
**Unknown lipid MB ESI pos (848450091)**	Unknown	0.13	0.23	0.39	0.58	0.67	**2.08**
**Unknown lipid MB ESI pos (848450092)**	Unknown	0.33	0.18	0.25	0.34	0.27	0.28
**Unknown lipid MB ESI pos (848450093)**	Unknown	0.36	0.27	0.26	0.81	0.79	0.84
**Unknown lipid MB ESI pos (848450096)**	Unknown	**2.12**	**2.16**	**2.13**	**2.95**	**3.05**	**2.15**
**Unknown lipid MB ESI pos (848450097)**	Unknown	**7.77**	**5.44**	**6.12**	**8.39**	**6.77**	**5.69**
**Unknown lipid MB ESI pos (848450100)**	Unknown	**4.45**	**3.95**	**3.29**	**3.66**	**5.95**	**3.82**
**Unknown lipid MB ESI pos (848450102)**	Unknown	**7.50**	**2.41**	**3.35**	**5.42**	**3.26**	**2.48**
**Unknown lipid MB ESI pos (848450103)**	Unknown	**6.23**	**3.76**	**3.00**	**5.19**	**3.47**	**2.37**
**Unknown lipid MB ESI pos (848450105)**	Unknown	**12.98**	**10.29**	**6.82**	**15.00**	**13.23**	**7.67**
**Unknown lipid MB ESI pos (848450106)**	Unknown	**6.12**	**2.96**	2.44	**3.13**	**3.79**	**2.63**
**Unknown lipid MB ESI pos (848450107)**	Unknown	**2.30**	1.48	**1.75**	1.35	**2.12**	**1.72**
**Unknown lipid MB ESI pos (848450108)**	Unknown	**5.08**	**2.74**	**2.99**	**2.58**	**2.86**	**2.64**
**Unknown lipid MB ESI pos (848450109)**	Unknown	**3.15**	**2.05**	**2.63**	1.98	**2.46**	1.99
**Unknown lipid MB ESI pos (848450110)**	Unknown	**7.61**	**5.39**	**4.38**	**5.29**	**5.97**	**8.43**
**Unknown lipid MB ESI pos (848450111)**	Unknown	**1.50**	**2.75**	**2.58**	**2.09**	**3.08**	**3.30**
**Unknown lipid MB ESI pos (848450113)**	Unknown	**3.44**	**3.72**	**5.06**	**3.20**	**4.44**	**5.06**
**Unknown lipid MB ESI pos (848450114)**	Unknown	**6.47**	**3.27**	**3.99**	**3.27**	**3.72**	**3.48**
**Unknown lipid MB ESI pos (848450116)**	Unknown	**4.56**	**3.33**	2.16	**3.56**	**6.39**	**4.97**

**Unknown lipid MB ESI pos (848450129)**	Unknown	**2.20**	**2.33**	1.92	**1.55**	**2.99**	**2.57**
**Unknown lipid MB ESI pos (848450131)**	Unknown	**4.68**	**3.56**	**3.12**	**3.13**	**6.07**	**3.01**
**Unknown lipid MB ESI pos (848450132)**	Unknown	**3.91**	**3.02**	**2.49**	**2.64**	**4.43**	**3.27**
**Unknown lipid MB ESI pos (848450138)**	Unknown	**2.98**	**2.43**	1.83	**2.38**	**4.26**	**3.24**
**Unknown lipid MB ESI pos (848450139)**	Unknown	**8.65**	**6.11**	**4.71**	**3.82**	4.64	2.80
**Unknown lipid MB ESI pos (848450142)**	Unknown	**11.91**	**6.51**	**4.84**	**5.92**	**4.02**	**3.44**
**Unknown lipid MB ESI pos (848450144)**	Unknown	**3.66**	**3.97**	**2.90**	**3.06**	**5.24**	**5.07**
**Unknown lipid MB ESI pos (848450146)**	Unknown	**3.20**	**2.65**	**1.89**	**2.57**	**3.73**	**2.89**
**Unknown lipid MB ESI pos (848450148)**	Unknown	**4.50**	**3.55**	**2.68**	**3.22**	**5.94**	**4.59**
**Unknown lipid MB ESI pos (848450152)**	Unknown	**1.97**	**2.88**	1.61	**1.76**	**4.76**	**3.04**
**Unknown lipid MB ESI pos (848450154)**	Unknown	0.55	0.43	0.31	0.40	0.97	0.50
**Unknown lipid MB ESI pos (848450155)**	Unknown	0.70	0.33	0.28	0.63	0.76	0.54
**Unknown lipid MB ESI pos (848450196)**	Unknown	**5.29**	**9.54**	**8.18**	**8.93**	**14.31**	**7.54**
**Unknown lipid MB ESI pos (848450201)**	Unknown	**5.57**	**7.29**	**6.98**	**9.37**	**10.26**	**6.95**
**Unknown lipid MB ESI pos (848450202)**	Unknown	**2.09**	**2.74**	**2.70**	2.96	**3.52**	**3.20**
**Unknown lipid MB ESI pos (848450204)**	Unknown	**7.45**	**10.29**	**8.78**	**12.73**	**13.86**	**9.12**
**Unknown lipid MB ESI pos (848450205)**	Unknown	**7.03**	**9.67**	**8.09**	**11.64**	**11.30**	**6.86**
**Unknown lipid MB ESI pos (848450206)**	Unknown	**8.01**	**13.99**	**13.73**	**11.48**	**20.22**	**11.64**
**Unknown lipid MB ESI pos (848450207)**	Unknown	**7.69**	**11.50**	**10.10**	**13.03**	**17.72**	**8.94**
**Unknown lipid MB ESI pos (848450208)**	Unknown	**7.05**	**9.59**	**8.78**	**9.85**	**11.25**	**7.56**
**Unknown lipid MB ESI pos (848450209)**	Unknown	**5.28**	**6.39**	**5.23**	**8.77**	**8.16**	**5.73**
**Unknown lipid MB ESI pos (848450210)**	Unknown	**3.96**	**6.09**	**6.68**	**8.06**	**10.12**	**7.77**
**Unknown lipid MB ESI pos (848450212)**	Unknown	**5.89**	**8.29**	**8.97**	**7.91**	**10.11**	**8.73**
**Unknown lipid MB ESI pos (848450213)**	Unknown	**7.01**	**9.67**	**9.44**	**11.47**	**11.65**	**8.07**
**Unknown lipid MB ESI pos (848450214)**	Unknown	**5.94**	**9.46**	**10.38**	**9.18**	**10.13**	**8.52**
**Unknown lipid MB ESI pos (848450215)**	Unknown	**6.71**	**11.15**	**8.18**	**9.93**	**11.66**	**8.99**
**Unknown lipid MB ESI pos (848450216)**	Unknown	**5.58**	**9.70**	**8.47**	**9.45**	**10.69**	**7.24**
**Unknown lipid MB ESI pos (848450218)**	Unknown	**5.65**	**7.70**	**8.38**	**9.63**	**10.05**	**7.73**
**Unknown lipid MB ESI pos (848450219)**	Unknown	**4.62**	**6.93**	**6.71**	**7.88**	**8.79**	**7.04**

**Unknown polar MB (838450146)**	Unknown	**5.09**	**3.02**	**2.11**	**2.07**	1.69	1.09
**Unknown polar MB (838450528)**	Unknown	0.60	0.25	0.13	1.00	1.33	1.21
**Unknown polar MB (838450590)**	Unknown	0.46	0.19	0.71	0.52	0.16	0.42
**Unknown polar MB (838450621)**	Unknown	**6.68**	**7.46**	**7.28**	**12.07**	**8.90**	**12.61**
**Unknown polar MB (838450692)**	Unknown	0.19	0.24	0.18	0.11	0.23	0.20
**Unknown polar MB (838450761)**	Unknown	**3.89**	**2.30**	**1.75**	**1.92**	**2.61**	**2.23**
**Unknown polar MB (838450816)**	Unknown	0.01	0.00	0.01	0.01	0.00	0.01
**Unknown polar MB (838450990)**	Unknown	1.53	**1.47**	**1.51**	1.16	1.05	1.05
**Unknown polar MB (838451614)**	Unknown	0.04	0.08	0.03	0.02	0.16	0.04
**Unknown polar MB (838452021)**	Unknown	0.59	0.38	0.49	0.41	0.46	0.52
**Unknown polar MB (838452022)**	Unknown	**2.60**	**2.14**	**1.75**	2.24	**1.62**	**2.06**
**Unknown polar MB (838452079)**	Unknown	**7.37**	**1.97**	**2.83**	**2.98**	**1.95**	**2.13**
**Unknown polar MB (838452178)**	Unknown	0.07	0.10	0.18	0.07	0.16	0.34
**Unknown polar MB (838452196)**	Unknown	**3.83**	**1.85**	**2.34**	**2.32**	1.44	1.90
**Unknown polar MB (838452804)**	Unknown	**10.48**	2.37	**4.70**	2.91	2.49	1.31
**Unknown polar MB (838452997)**	Unknown	0.32	0.55	0.40	0.34	0.59	0.42
**Unknown polar MB (838452998)**	Unknown	1.86	0.20	0.19	1.04	0.40	0.80
**Unknown polar MB (838453005)**	Unknown	0.20	0.27	0.26	0.09	0.09	0.09
**Unknown polar MB (838453006)**	Unknown	0.17	0.24	0.21	0.09	0.30	0.24
**Unknown polar MB (838453008)**	Unknown	0.18	0.32	0.26	1.19	0.98	1.43
**Unknown polar MB (838453009)**	Unknown	0.02	0.03	0.02	0.03	0.04	0.02
**Unknown polar MB (838453011)**	Unknown	0.51	0.69	0.36	0.74	1.06	0.65
**Unknown polar MB (838453012)**	Unknown	**26.42**	**24.33**	**11.72**	**215.16**	**83.27**	**83.04**
**Unknown polar MB (838453014)**	Unknown	0.21	0.32	0.61	0.76	0.67	1.06
**Unknown polar MB (838453015)**	Unknown	0.18	0.11	0.18	0.19	0.12	0.17
**Unknown polar MB (838453016)**	Unknown	0.33	0.54	0.53	1.27	**1.23**	2.00
**Unknown polar MB (838453017)**	Unknown	0.11	0.05	0.10	0.08	0.14	0.09
**Unknown polar MB (838453018)**	Unknown	0.07	0.11	0.17	0.07	0.17	0.12
**Unknown polar MB (838453019)**	Unknown	0.10	0.06	0.03	0.08	0.08	0.02
**Unknown polar MB (838453026)**	Unknown	0.24	0.09	0.17	0.19	0.28	0.40
**Unknown polar MB (838453028)**	Unknown	0.18	0.08	0.07	0.11	0.07	0.06
**Unknown polar MB (838453033)**	Unknown	0.14	0.06	0.06	0.05	0.07	0.04
**Unknown polar MB (838453036)**	Unknown	0.07	0.03	0.02	0.06	0.06	0.04
**Unknown polar MB (838453040)**	Unknown	0.05	0.03	0.03	0.03	0.06	0.03
**Unknown polar MB (838453041)**	Unknown	0.07	0.11	0.11	0.10	0.08	0.18
**Unknown polar MB (838453044)**	Unknown	0.36	0.30	0.36	0.43	0.39	0.40
**Unknown polar MB (838453047)**	Unknown	0.20	0.04	0.04	0.11	0.10	0.05
**Unknown polar MB (838453048)**	Unknown	0.12	0.11	0.07	0.15	0.09	0.11
**Unknown polar MB (838453051)**	Unknown	0.28	0.51	0.50	0.34	0.65	0.75
**Unknown polar MB (838453052)**	Unknown	0.28	0.15	0.25	0.17	0.17	0.12
**Unknown polar MB (838453053)**	Unknown	0.32	0.16	0.33	0.17	0.14	0.13
**Unknown polar MB (838453055)**	Unknown	0.04	0.03	0.02	0.03	0.05	0.02
**Unknown polar MB (838453056)**	Unknown	0.38	0.29	0.26	0.27	0.19	0.28
**Unknown polar MB (838453057)**	Unknown	0.44	0.25	0.38	0.25	0.20	0.16
**Unknown polar MB (838453058)**	Unknown	0.56	0.17	0.21	0.37	0.11	0.12
**Unknown polar MB (838453059)**	Unknown	**2.66**	**2.95**	**3.19**	**2.35**	**3.17**	**2.57**
**Unknown polar MB (838453062)**	Unknown	0.30	0.43	0.49	0.43	0.62	0.57

**Unknown polar MB (838453063)**	Unknown	0.30	0.03	0.03	0.13	0.03	0.05
**Unknown polar MB (838453066)**	Unknown	0.01	0.01	0.01	0.01	0.00	0.01
**Unknown polar MB (838453067)**	Unknown	**3.19**	**2.27**	**3.10**	**4.49**	**2.27**	**3.68**
**Unknown polar MB ESI pos (868450034)**	Unknown	**3.53**	**4.44**	**3.47**	0.52	0.88	0.68
**Unknown polar MB ESI pos (868450040)**	Unknown	**3.29**	4.00	**4.83**	**1.97**	0.95	1.49
**Uric acid**	Nucleobases and related	**10.52**	8.98	**7.47**	0.35	1.23	0.72
**Valine**	Amino acids	50.71	**53.51**	**93.37**	0.87	0.44	0.82
**Valine TMS ME**	Unknown	2.68	**3.55**	**4.64**	0.65	0.70	0.69
**Valine, lipid fraction**	Miscellaneous	3.25	**3.07**	**3.89**	0.94	0.67	0.62
**Xanthine**	Nucleobases and related	**88.44**	**92.29**	**74.95**	0.66	0.55	2.40
**Xanthurenic acid**	Amino acids related	0.08	0.12	0.05	0.05	0.07	0.11
**Xylose**	Carbohydrates and related	**11.50**	**23.67**	**4.83**	**4.22**	3.29	**2.14**

Table S5: Summarized table indicating the trend of significant alterations in plasma biomarkers including hippuric acid and indole derivatives in samples from animals treated with antibiotics (HD group only) belonging to different classes. Yellow boxes indicate a significant increase and red box indicates a significant decrease in the fold change levels of respective plasma metabolites at p-value<0.1.

Plasma biomarkers	Aminoglycosides			Lincosamides		Fluoroquinolone			Carbapenems	
	Tobramycin	Gentamycin	Neomycin	Clindamycin	Lincomycin	Sparfloxacin	Moxifloxacin	Levofloxacin	Meropenem	Doripenem
3-Indoxylsulfate (3IS)	↓↓	NS	NS	↓↓	↓	NS	↓↓	NS	↓↓	↓↓
Hippuric acid (HA)	↓↓	↓↓	↓↓	↓↓	↓	NS	↓↓	↓↓	↓↓	↓↓
Indole-3-acetic acid (IAA)	↓↓	NS	↓	NS	NS	↓↓	↓↓	↓↓	↑	↓
Indole-3-propionic acid (IPA)	↓↓	NA	↓↓	NA	NA	NA	NS	NS	NS	↓

*NS, non-significant; NA, not applicable
